# Resuscitation fluid types in sepsis, surgical, and trauma patients: a systematic review and sequential network meta-analyses

**DOI:** 10.1186/s13054-020-03419-y

**Published:** 2020-12-14

**Authors:** Chien-Hua Tseng, Tzu-Tao Chen, Mei-Yi Wu, Ming-Cheng Chan, Ming-Chieh Shih, Yu-Kang Tu

**Affiliations:** 1 Institute of Epidemiology and Preventive Medicine, National Taiwan University, Room 539, No. 17, Xu-Zhou Rd., Taipei, 10055 Taiwan; 2 Division of Pulmonary Medicine, Department of Internal Medicine, School of Medicine, College of Medicine, Taipei Medical University, Taipei, Taiwan; 3 Division of Critical Care Medicine, Department of Emergency and Critical Care Medicine, Shuang Ho Hospital, Taipei Medical University, New Taipei City, Taiwan; 4 Division of Pulmonary Medicine, Department of Internal Medicine, Shuang Ho Hospital, Taipei Medical University, New Taipei City, Taiwan; 5 Division of Nephrology, Department of Internal Medicine, Shuang Ho Hospital, Taipei Medical University, New Taipei City, Taiwan; 6 Division of Nephrology, Department of Internal Medicine, School of Medicine, College of Medicine, Taipei Medical University, Taipei, Taiwan; 7 Division of Critical Care and Respiratory Therapy, Department of Internal Medicine, Taichung Veterans General Hospital, Taichung, Taiwan; 8 College of Science, Tunghai University, Taichung, Taiwan; 9 Department of Dentistry, National Taiwan University Hospital, National Taiwan University, Taipei, Taiwan; 10 Research Center of Big Data and Meta-Analysis, Wan Fang Hospital, Taipei Medical University, Taipei, Taiwan

**Keywords:** Fluid therapy, Intensive care, Resuscitation, Colloids, Crystalloids

## Abstract

**Background:**

Crystalloids and different component colloids, used for volume resuscitation, are sometimes associated with various adverse effects. Clinical trial findings for such fluid types in different patients’ conditions are conflicting. Whether the mortality benefit of balanced crystalloid than saline can be inferred from sepsis to other patient group is uncertain, and adverse effect profile is not comprehensive. This study aims to compare the survival benefits and adverse effects of seven fluid types with network meta-analysis in sepsis, surgical, trauma, and traumatic brain injury patients.

**Methods:**

Searched databases (PubMed, EMBASE, and Cochrane CENTRAL) and reference lists of relevant articles occurred from inception until January 2020. Studies on critically ill adults requiring fluid resuscitation were included. Intervention studies reported on balanced crystalloid, saline, iso-oncotic albumin, hyperoncotic albumin, low molecular weight hydroxyethyl starch (L-HES), high molecular weight HES, and gelatin. Network meta-analyses were conducted using random-effects model to calculate odds ratio (OR) and mean difference. Risk of Bias tool 2.0 was used to assess bias. Confidence in Network Meta-Analysis (CINeMA) web application was used to rate confidence in synthetic evidence.

**Results:**

Fifty-eight trials (*n* = 26,351 patients) were identified. Seven fluid types were evaluated. Among patients with sepsis and surgery, balanced crystalloids and albumin achieved better survival, fewer acute kidney injury, and smaller blood transfusion volumes than saline and L-HES. In those with sepsis, balanced crystalloids significantly reduced mortality more than saline (OR 0.84; 95% CI 0.74–0.95) and L-HES (OR 0.81; 95% CI 0.69–0.95) and reduced acute kidney injury more than L-HES (OR 0.80; 95% CI 0.65–0.99). However, they required the greatest resuscitation volume among all fluid types, especially in trauma patients. In patients with traumatic brain injury, saline and L-HES achieved lower mortality than albumin and balanced crystalloids; especially saline was significantly superior to iso-oncotic albumin (OR 0.55; 95% CI 0.35–0.87).

**Conclusions:**

Our network meta-analysis found that balanced crystalloids and albumin decreased mortality more than L-HES and saline in sepsis patients; however, saline or L-HES was better than iso-oncotic albumin or balanced crystalloids in traumatic brain injury patients.

***Trial registration*:**

PROSPERO website, registration number: CRD42018115641).

## Introduction

Fluid resuscitation is one of the most common and important methods in managing critically hypotensive patients. Crystalloids, mineral salts, or other water-soluble molecule solutions have been used for more than 100 years for fluid resuscitation [1, 2]. In the past decades, several colloids, larger insoluble molecular solutions, have been developed to improve intravascular volume more effectively. However, since the integrity of the endothelial glycocalyx layer might be interrupted under inflammatory conditions, such as sepsis, surgery, trauma, or traumatic brain injury, evaluation of the efficacy and safety of colloids in such patients is challenging [3, 4].

Insoluble molecules in colloids include starch, bovine protein (gelatin), and human protein (albumin). Hydroxyethyl starch (HES) of higher molecular weight has a longer half-life in plasma, but it reduces plasma coagulation factors more than HES of lower molecular weight [5] and albumin [6]. Starch macromolecule accumulation also impairs glomerular filtration and is associated with a higher risk of acute renal failure than gelatin [7]; however, gelatin is associated with a higher incidence of anaphylactic shock [8, 9]. Compared to iso-oncotic albumin, hyperoncotic albumin leads to a higher osmotic pressure, which may alter intraglomerular oncotic force and osmotic nephrosis, and is associated with worse kidney damage [10]. Chemical components, molecular weights, and colloid concentration might expose the human body to different levels of hazards [11]. Among crystalloids, saline worsens acidosis and bleeding tendency compared to balanced crystalloids [12]. Consequently, classifying resuscitation fluids into either colloids or crystalloids was no longer enough.

From 2012 to 2018, of 15 meta-analyses published on fluid resuscitation in critically ill patients (Additional file 1: appendix pp. 5–7), 12 (80%) grouped high and low chloride crystalloids or colloids of different components into a single type of treatment, and 5 (33.3%) grouped sepsis, surgical, and trauma patients into one meta-analysis. Furthermore, no meta-analyses compared the required fluid volumes for the resuscitation target. This study aimed to compare the survival benefit and any potential adverse effects of seven fluid types using network meta-analysis (NMA) in sepsis, surgical, trauma, and traumatic brain injury patients, and investigated the trend in treatment difference using sequential NMA.

## Methods

### Data sources and searches

We registered our systematic review process on the PROSPERO website [13] (registration number: CRD42018115641). This NMA followed the preferred reporting items for systematic reviews and meta-analyses (PRISMA) extension guideline which incorporated NMA for healthcare interventions (Additional file 1: appendix pp. 8–13) [14]. Electronic databases, including PubMed, EMBASE, and Cochrane CENTRAL, were searched from their inception until January 2020. The search strategies combined terms for patients’ conditions, clinical outcomes, and fluid types (Additional file 1: appendix pp. 14–15).

### Study selection

We included randomized controlled trials (RCTs) on critically ill adult patients (more than 18 years old) who presented with systemic hypoperfusion and required fluid resuscitation. We excluded trials on children with dengue fever, those on burn injury patients, or those on mixed populations without reporting subgroup data (Additional file 1: appendix pp. 17–20).

### Data extraction and quality assessment

We divided patients requiring fluid resuscitation into the following groups for extraction of data and separate analyses: sepsis, surgical, trauma, and traumatic brain injury. The 7 interventions included 2 crystalloids [balanced crystalloids, including lactated Ringer’s, Ringer acetate or PlasmaLytes and saline (0.9% sodium chloride)], and 5 colloids [iso-oncotic albumin (4%, 5%); hyperoncotic albumin (20%, or 25%); HES with molecular weight ≦ 130 k (L-HES); HES with molecular weight ≧ 200 k (H-HES); and gelatin]. The outcomes included:

1. All-cause mortality rate. If a study reported outcomes at multiple time points, we chose the longest observation.
2. Fluid resuscitation volume. The resuscitation target is the reversal of organ hypoperfusion.
3. Acute kidney injury, referring to the degree of renal dysfunction, based on a 5-level scoring system to evaluate risk, injury, failure, loss, and end-stage renal failure (RIFLE).
4. Transfusion volume.
5. Allergic reaction rate.

Two authors (CH Tseng and TT Chen) screened the studies on RCTs independently, extracted data, and assessed the risk of bias of studies using the revised Cochrane risk of bias tool (RoB 2 tool) at study level [15]. A third reviewer (YK Tu) was consulted to resolve any disagreement in data extraction or assessment.

### Data synthesis and analysis

Transitivity assumption was assessed by checking the distribution of potential confounding variables across studies grouped by interventions. The variables examined included age, male percentage, disease severity scores, source of sepsis from the lung, and publication year. We first used the “network” suite of STATA version 14.0 [16] (StataCorp, Texas, USA) statistical software, which implements a frequentist approach to the contrast-based model meta-analyses [16], to undertake a random-effect NMA [17]. We then used network map to illustrate the distribution of the direct and indirect evidence between all treatment comparisons. The size of the nodes in the map was proportional to the number of patients who received this intervention in the network, and the width of the edges was proportional to the number of trials that compared the two treatments. Certainty of the evidence was assessed using CINeMA (Confidence in Network Meta-Analysis) web application, which allows for confidence in the results to be graded as high, moderate, low, and very low. This approach was based on a methodology developed by the Grading of Recommendations Assessment, Development and Evaluation Working Group for pairwise meta-analyses [18].

Surface under the cumulative ranking (SUCRA) probabilities is the ratio of the area under the cumulative ranking curve to the entire area in the plot. The more quickly the cumulative ranking curve approaches one, the closer to unity this ratio is. SUCRA values may be seen as the percentage of safety a treatment achieves in relation to an imaginary treatment that is always the best without any uncertainty [19]. To adjust for the multiplicity of statistical testing, we further conducted sequential NMA, proposed by Nikolakopoulor et al., who extended the rationales of sequential meta-analyses for defining sample-path, efficacy boundaries, futility boundaries, and information size in meta-analyses [20]. In sequential NMA, we undertook a series of NMA, providing a path of estimates for each pairwise comparison, by including studies incrementally into the analysis according to their publication years [20]. When the path crossed the efficacy boundaries, defined by the α-spending function derived from the O’Brien–Fleming method [21], the difference between the two treatments exceeded the threshold for statistical significance. In contrast, when the path fell within the futility area defined by the β-spending functions [22], the two interventions showed no difference in their effects. We used the R software package “sequentialnma” to undertake sequential NMA [23]. Results from these additional analyses were then compared to the results from the NMA.

## Results

**Fig. 1:**
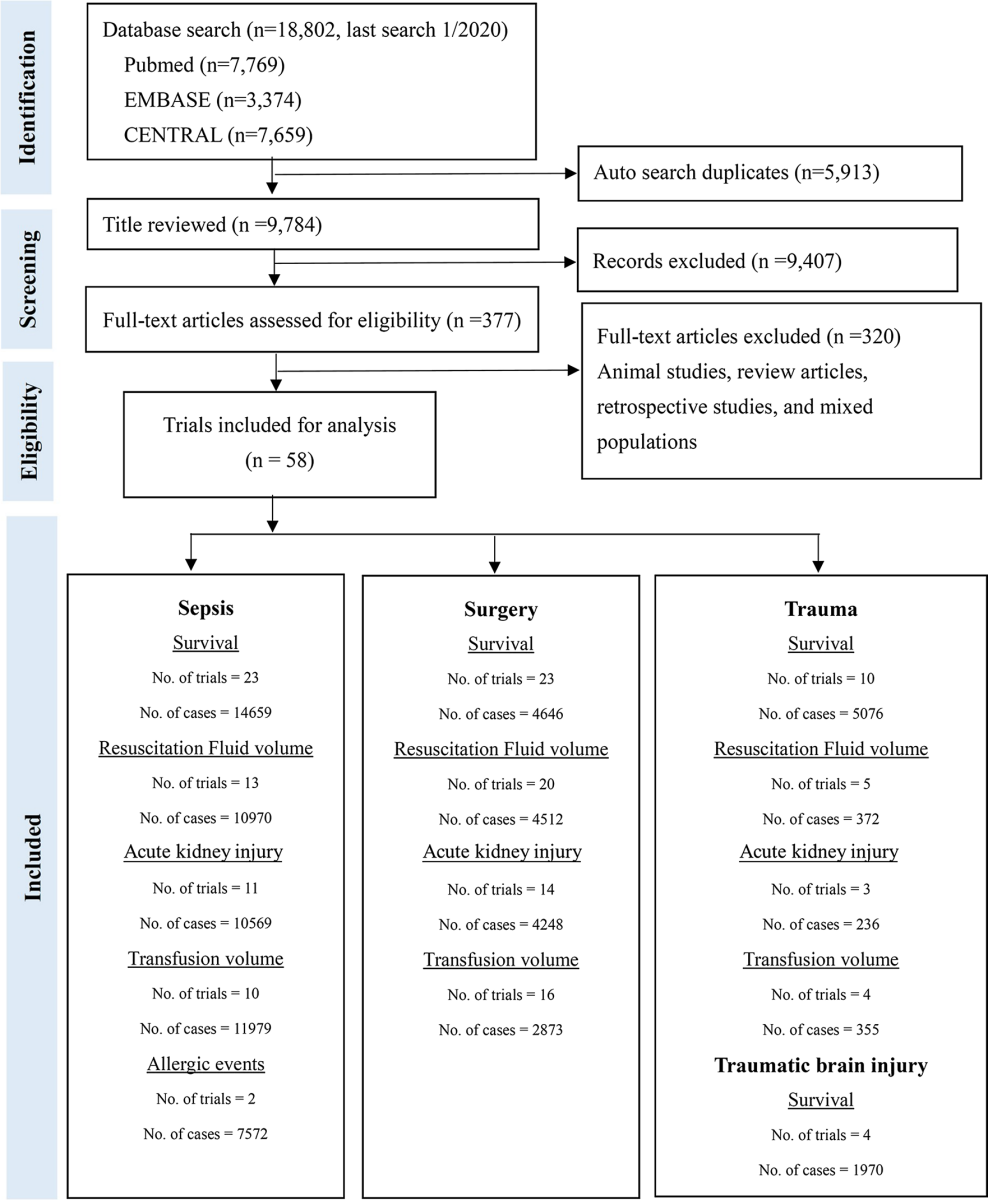
Summary of evidence search and selection

The literature search identified 18,802 citations, and 377 full-text articles were assessed for eligibility. Of 58 RCTs which included 26,351 patients in the analysis, 5 large RCTs included more than one condition—sepsis, surgery, trauma, and traumatic brain injury. Thus, we extracted the subgroup data of patients with different conditions. As a result, 23 RCTs on sepsis patients, 24 on surgical patients, 10 on trauma patients, and 4 on traumatic brain injury patients were included for further analysis (Fig. 1, Additional file 1: appendix pp. 17–48). We present the risk of bias assessment for each included study in Additional file 1: appendix 7 (appendix pp. 61–70); eFigure 7.1 shows the overall risk of bias in five domains in sepsis trials, eFigure 7.2 shows the risk of bias for the individual studies, and eFigure 7.3 explains the reasons for upgrading or downgrading in every study (Additional file 1: appendix pp. 60–63, 64–66, 67–69). The reasons to downgrade are mostly inadequate randomization process, open-labeled design, or no detailed information. No significant differences in baseline variables between interventions were observed within our NMA (Additional file 1: appendix pp. 49–60).

### Sepsis patients

Most RCTs used the 2001 International Sepsis Definitions Conference sepsis definition [24] and included sepsis patients with shock status or those who had evidence of tissue or organ hypoperfusion (Additional file 1: eTable 5.2, appendix pp. 23–26). The timing for fluid resuscitation is when the patient meets the enrollment criteria: systemic hypoperfusion defined by low blood pressure, low central venous pressure or wedge pressure and elevated lactate level. We compared the mean arterial pressure among interventions at baseline (Additional file 1: appendix pp. 56), ranging from 69.0 to 73.9 mmHg, and found no statistically significant differences among seven fluid types. Besides, in Additional file 1: eTable 5.4 (appendix pp. 29–31), and Additional file 1: eTable 5.6 (appendix pp. 35–36), we compared resuscitation targets among included trials. The resuscitation goals are generally to maintain wedge pressure around 15–18 mmHg or central venous pressure around 8–12 mmHg. The average mean study fluid volume was 2397.4 mL ± 1019.1 mL in each arm, and the total resuscitation fluid volume was 7615.6 mL ± 1729.7 mL (Additional file 1: appendix pp. 22–31, 61–64). In Additional file 1: eTable 5.3 (appendix pp. 26–27), we presented the baseline characteristics, including age, severity of illness, mean arterial pressure, and lactate level.

#### Sepsis patients—mortality

**Fig. 2:**
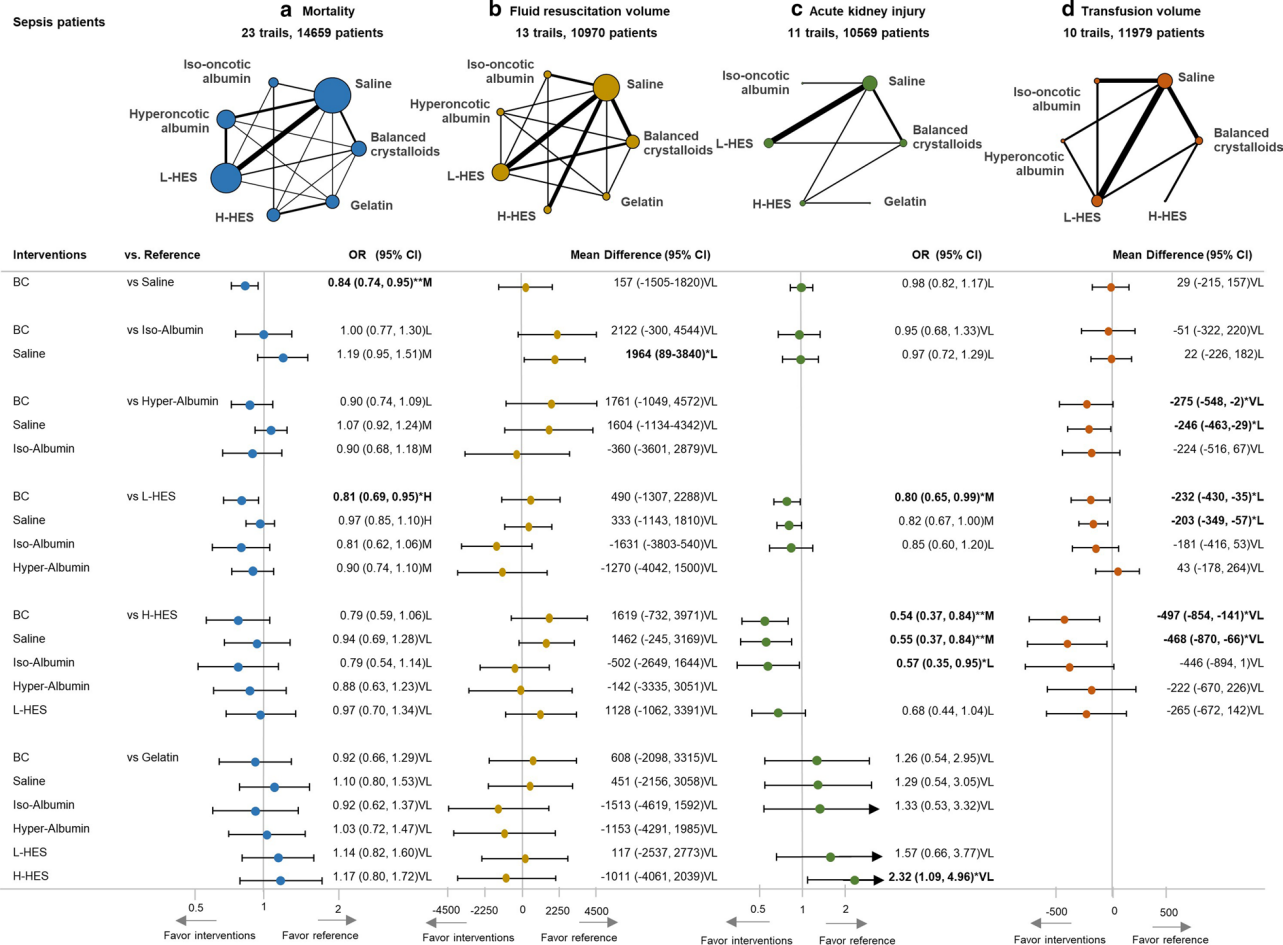
Network geometry and forest plot in sepsis patients with four outcomes. **a** Mortality, **b** fluid resuscitation amount, **c** acute kidney injury, **d** transfusion amount. The difference among each comparison is visualized with forest plot, and the effect size and evidence rating are labeled on the right-hand side. The bold characters are to emphasize significant contrasts. The 95% confidence intervals in the forest plot are clipped to arrows, when they exceed the limit of x-axis. Abbreviations: *OR* odds ratio; *p < 0.0.5; **p < 0.01; *H* high confidence rating, *M* moderate confident rating, *L* low confidence rating, *VL* very low confidence rating, *BC* balanced crystalloids, *Iso-albumin* iso-oncotic albumin, *Hyper-albumin* hyperoncotic albumin, *L-HES* low molecular weight hydroxyethyl starch, *H-HES* high molecular weight hydroxyethyl starch

**Fig. 3:**
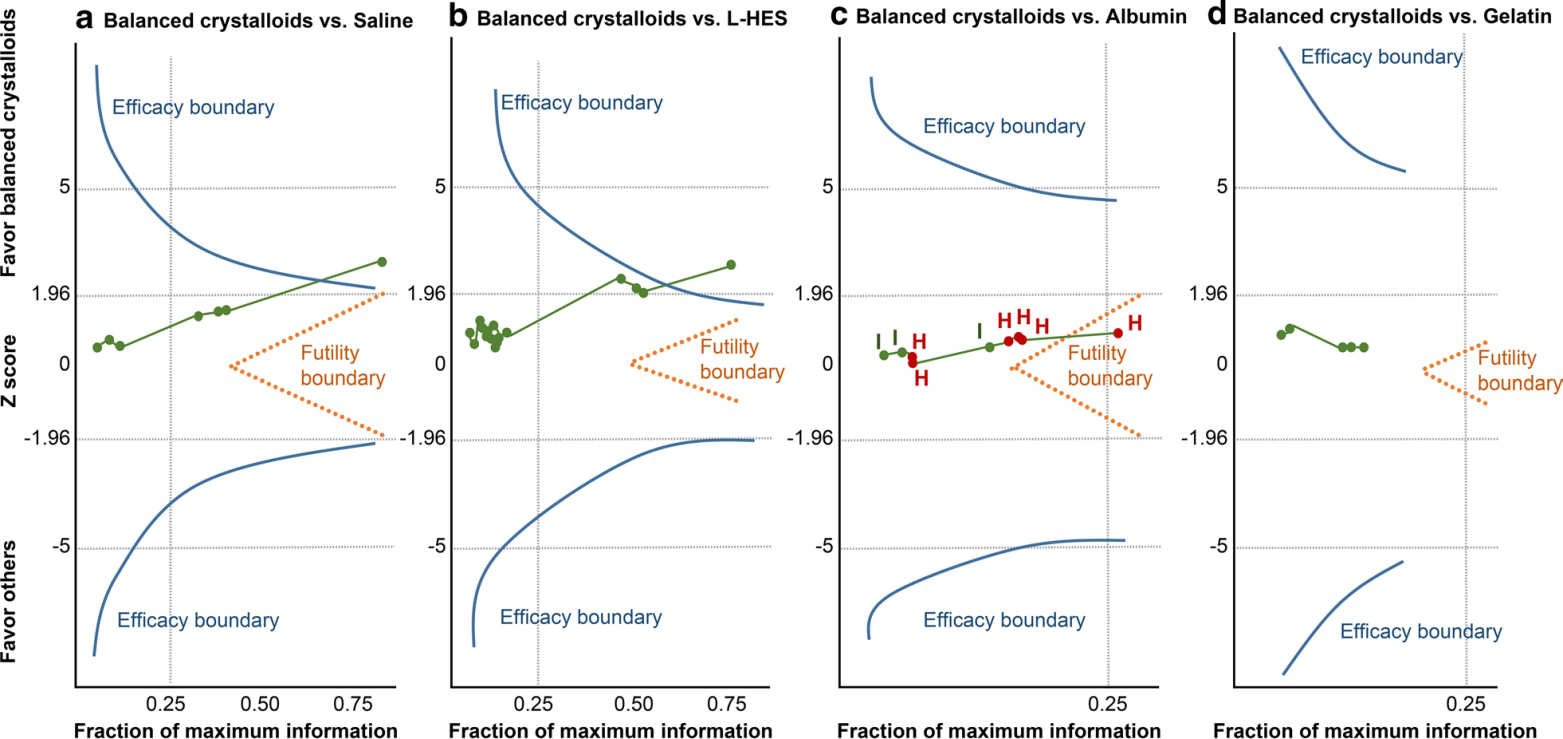
Sequential network meta-analyses (SNMA) over sepsis patient mortality analysis among **a** balanced crystalloids versus saline, **b** balanced crystalloids versus low molecular weight hydroxyethyl starch (L-HES), **c** balanced crystalloids versus albumin, and **d** balanced crystalloids versus gelatin. Y-axis represent the z scores for effect sizes, and green dots (trials) and green line along the X-axis show the trend of cumulating evidence toward achieving maximal information. The blue line represents the SNMA efficacy boundary, and orange line represents the futility boundary. The green dots and green line start in the middle; when they pass the blue line, this indicates that a significant difference in the outcome between the two treatments has been attained. When they pass the orange line, this suggests no difference in the outcome between the two treatments. *I* iso-oncotic albumin, *H* hyperoncotic albumin

**Fig. 4:**
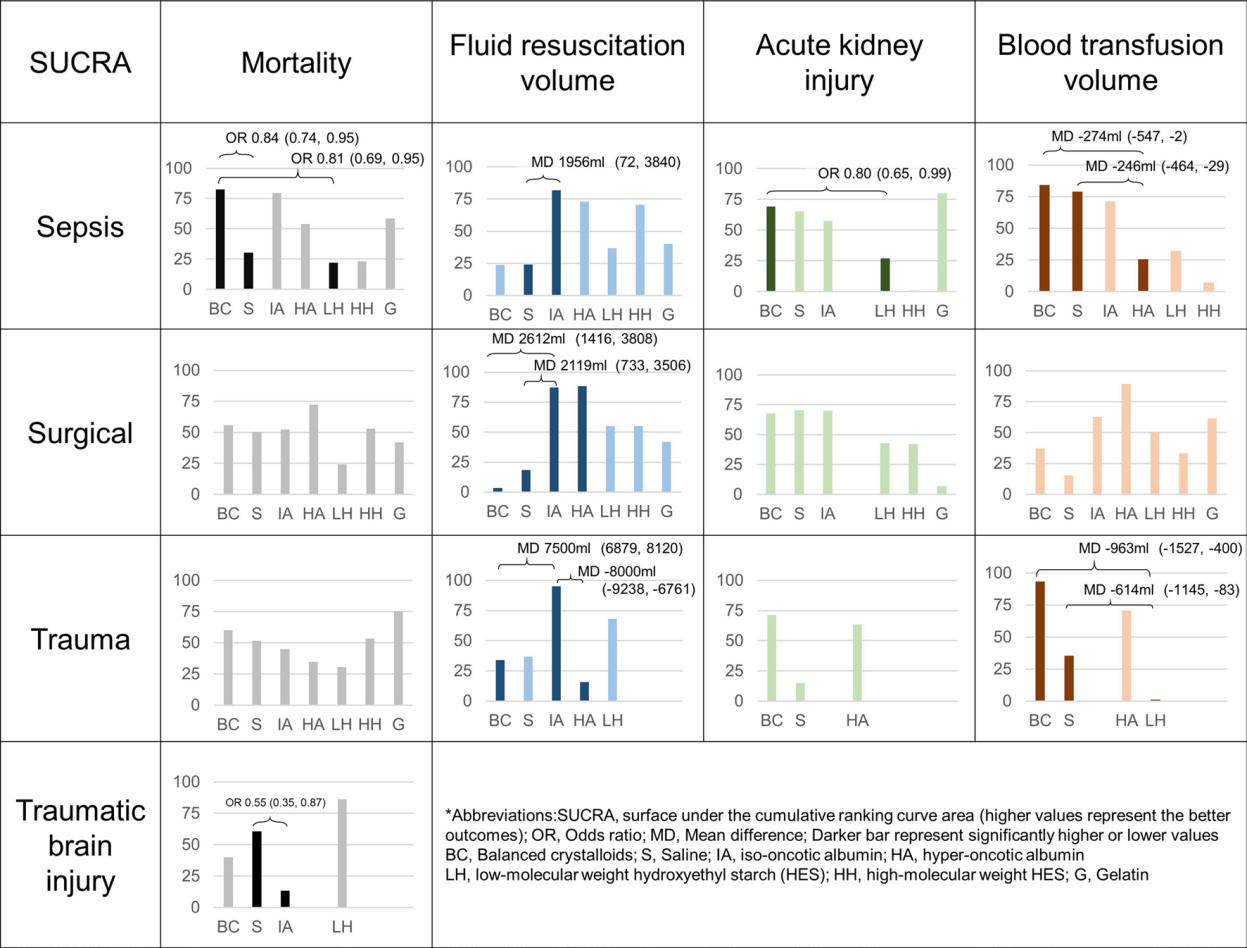
Surface under the cumulative ranking curve area (SUCRA) for mortality, fluid resuscitation volume, acute kidney injury, and blood transfusion volume among sepsis, surgical, trauma, and traumatic brain injury patients. Dark color bar represents significantly better or worse interventions, and the differences between fluid types are shown above the bars

Between 1983 and 2018, 23 RCTs with 14,659 participants presented with usable results on mortality. In Additional file 1: appendix eTable 5.1 (Additional file 1: appendix pp. 21), we provided the details of mortality outcome used in our analysis, including in-hospital mortality, 30 day-mortality, and 90-day mortality. If multiple time points were reported in a study, we chose the longest observation period for mortality analysis. Balanced crystalloids reduced mortality more than saline and L-HES with odds ratios (OR) of 0.84 (95% CI 0.74–0.95) and 0.81 (95% CI 0.69–0.95), respectively (Fig. 2a). Sequential NMA further supported the difference in mortality rate between balanced crystalloids versus saline and L-HES by demonstrating that the trend in cumulative evidence exceeded the efficacy boundary. The cumulative evidence exceeded the futility boundary in the comparison between balanced crystalloids and albumin, but fell between efficacy and futility boundary in the comparison between balanced crystalloids and gelatin (Fig. 3). According to SUCRA, balanced crystalloid appeared to be the best option; however, saline, L-HES, and H-HES were not favored (Fig. 4).

#### Sepsis patients—fluid resuscitation volume

Thirteen trials with 10,970 participants reported usable results for fluid resuscitation volume in sepsis patients. Balanced crystalloids and saline required more fluid volume than iso-oncotic albumin with mean differences (MD) of 2122 mL (95% CI − 300 to 4544 mL) and 1964 mL (95% CI 89–3840 mL), respectively (Fig. 2b). SUCRA revealed that the colloids were associated with less resuscitation fluid volume than crystalloids (Fig. 4).

#### Sepsis patients—acute kidney injury

Eleven trials with 10,569 participants reported usable results for acute kidney injury. Balanced crystalloids significantly reduced a greater risk of acute kidney injury than L-HES (OR, 0.80; 95% CI 0.65–0.99) and H-HES (OR, 0.54; 95% CI 0.37–0.84) (Fig. 2c). SUCRA ranking revealed that gelatin, balanced crystalloid, saline, and iso-oncotic albumin had a lower risk of acute kidney injury than L-HES and H-HES (Fig. 4).

#### Sepsis patients—red blood cell transfusion volume

Ten trials with 11,979 participants reported usable results for the packed red blood cell transfusion volume. Balanced crystalloids required less volume of red blood cell transfusion than hyperoncotic albumin (MD, 274 mL; 95% CI 5–548 mL), L-HES (MD, 232 mL; 95% CI 35–430 mL), and H-HES (MD, 497 mL; 95% CI 141–854 mL). (Fig. 2d). SUCRA revealed that the crystalloids and iso-oncotic albumin were associated with less transfusion volume than other colloids (Fig. 4).

The funnel plot and Egger’s test did not detect any significant publication bias (Additional file 1: appendix pp. 114–116). Loop inconsistency and design inconsistency were also not detected (Additional file 1: appendix pp. 124–129). The meta-regression did not change the ranking order (Additional file 1: appendix pp. 138–139). The evidence certainty in mortality revealed a moderate-to-high evidence confidence in comparison, including balanced crystalloids, saline, and L-HES; low-to-moderate in iso-oncotic albumin and hyperoncotic albumin; and very low in gelatin and H-HES (Additional file 1: appendix pp. 139–142). Results of sensitivity analyses with the exclusion of the largest SMART trials [12] or the inclusion of the pilot SALT trial [25] in Additional file 1: appendix 14 show no substantial differences from the main analysis.

### Surgical patients

From 1979 to 2020, 8 (34.80%), 6 (26.00%), 6 (26.00%), and 3RCTs compared different resuscitation fluids in patients receiving cardiac surgery, aortic surgery, major abdominal surgery, and hip arthroplasty and cystectomy, respectively (Additional file 1: appendix pp. 32–36). Fluid resuscitation was provided during surgical procedures to maintain hemodynamic parameters in most trials, and the mean resuscitated fluid of interest was 3327.5 mL (Additional file 1: appendix 65–67).

#### Surgical patients—mortality

**Fig. 5:**
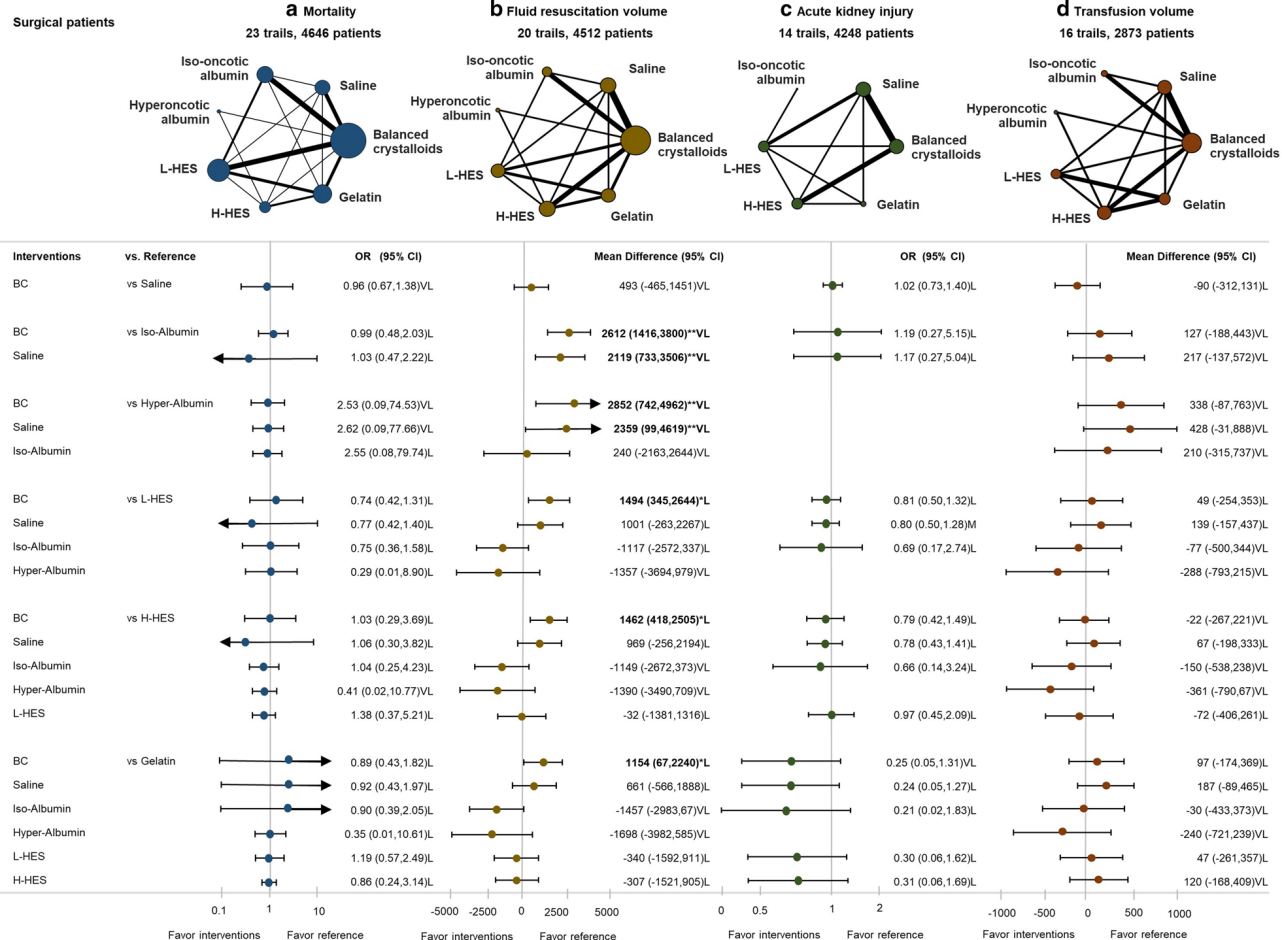
Network geometry and forest plot in surgical patients with four outcomes. **a** Mortality, **b** fluid resuscitation amount, **c** acute kidney injury, **d** transfusion amount. The difference among each comparison is visualized with forest plot, and the effect size and evidence rating are labeled on the right-hand side. The bold characters are to emphasize significant contrasts. The 95% confidence intervals in the forest plot are clipped to arrows, when they exceed the limit of x-axis. *OR* odds ratio; *p < 0.05; **p < 0.01; *H* high confidence rating, *M* moderate confident rating, *L* low confidence rating, *VL* very low confidence rating, *BC* balanced crystalloids, *Iso-albumin* iso-oncotic albumin, *Hyper-albumin* hyperoncotic albumin, *L-HES* low molecular weight hydroxyethyl starch, *H-HES* high molecular weight hydroxyethyl starch

Twenty-three trials with 4646 participants had valid results on mortality. There were no significant differences in mortality between 7 interventions (Fig. 5); SUCRA showed that hyperoncotic albumin and balanced crystalloid were associated with less mortality than gelatin, HES, and saline (Fig. 4).

#### Surgical patients—fluid resuscitation volume

Twenty trials with 4512 participants provided data on resuscitation fluid volume. Balanced crystalloids group required significantly more fluid resuscitation volume than iso-tonic albumin (MD, 2612 mL; 95% CI 1416–3800), hypertonic albumin (MD, 2852 mL; 95% CI 742–4962), L-HES (MD 1494 mL; 95% CI 345–2644), H-HES (MD, 1462 mL; 95% CI 418–2505), and gelatin (MD, 1154 mL; 95% CI 64–2240) (Fig. 5). SUCRA ranking showed that colloids (albumin, HES, and then gelatin) were associated with less fluid resuscitation volume than crystalloids (Fig. 4).

#### Surgical patients—acute kidney injury

Fourteen trials with 4248 participants reported results for acute kidney injury. The ORs between seven treatments were not statistically significant (Fig. 5). SUCRA showed iso-oncotic albumin, and balanced crystalloids were associated with less acute kidney injury than HES and gelatin.

#### Surgical patients—red blood cell transfusion volume

Sixteen trials with 2818 participants presented usable results for red blood cell transfusion volume. Ranking probabilities showed that albumin, L-HES, and then gelatin were associated with less transfusion volume than H-HES and crystalloids (Fig. 5).

Publication bias and inconsistency were not significant (Additional file 1: appendix pp. 118–121). The confidence ratings were low to very low among all comparisons in surgical trials (Additional file 1: appendix pp. 143–146).

### Trauma and traumatic brain injury patients

From 1977 to 2018, 10 RCTs compared different resuscitation fluids in trauma patients who required fluid resuscitations, and 4 RCTs in traumatic brain injury patients. (Additional file 1: appendix pp. 37–39). Patients’ mean age was 48.6 years, predominantly male (69.8%), and mean resuscitation study fluid was 5481 mL among trauma trials. (Additional file 1: appendix pp. 82–86).

Ten trials with 5076 participants had valid results on mortality in trauma patients, and differences in mortality were not significant between interventions in trauma patients. Balanced crystalloid required less volume of red blood cell transfusion than saline (MD, 350 mL; 95% CI 160 mL to 540 mL) and L-HES (MD, 964 mL; 95% CI 400 mL to 1527 mL). Four trials with 1970 participants had valid results on mortality in traumatic brain injury patients, and saline reduced mortality than albumin with OR of 0.55 (95% CI 0.35–0.87) (Additional file 1: appendix pp. 103–114). The confidence ratings were low to very low among all comparisons in traumatic and traumatic brain injury trials (Additional file 1: appendix pp. 123–124, 128, 148–150).

## Discussion

To our knowledge, this analysis is the largest NMA in the field of fluid resuscitation, as we considered a larger number of outcomes and undertook separate analyses for patients with different conditions. In sepsis patients, balanced crystalloids and iso-oncotic albumin were associated with lower mortality rates, lower risks of acute kidney injury, and less red blood cell transfusion volume. In surgical patients, nonsignificant differences in the risks of mortality and acute kidney injury were observed between the seven interventions, but balanced crystalloids required the greatest volume of fluid resuscitation among all fluid types. In traumatic brain injury trials, iso-oncotic albumin was associated with higher mortality than saline.

### Previous studies and important differences from this study

In many previous meta-analyses on fluid resuscitation, sepsis, surgical, trauma, and traumatic brain injury patients were put together as a single group. In 2013, Perel et al. published a meta-analysis [26] of critically ill patients of all causes, and another meta-analysis on HES [27], including patients with different causes being grouped together. Our analyses separated patients’ conditions, thereby providing more precise information applied to specific subgroups of patients. Furthermore, previous meta-analyses also combined different fluid types into a single treatment. Our network meta-analysis used a more comprehensive classification of resuscitation fluids according to the current knowledge, yielding more clinically meaningful information.

### Crystalloids: balanced crystalloids and saline

Several meta-analyses and current sepsis guideline recommended that crystalloids are the fluid of choice for resuscitation [26, 28]. The present study found that among crystalloids, balanced crystalloids show better survival benefit and renal function for sepsis and surgical patients than saline does, and the reverse was found in traumatic brain injury patients. Instead of considering crystalloids as one treatment group, we could be more specific in considering balanced crystalloids for sepsis and surgical patients, and saline for traumatic brain injury patients. However, both crystalloids required a higher volume to achieve resuscitation goals. Therefore, in addition to evaluate fluid responsiveness with passive leg raising or other static tests continuously, choosing optimal fluid types could also prevent fluid overload. [29].

### Albumin: iso-oncotic and hyperoncotic albumin

The osmotic pressure in iso-oncotic solution was similar to plasma, and hyperoncotic solution was higher than plasma. Iso-oncotic albumin was designed for fluid resuscitation and has volume-sparing effect; hyperoncotic albumin was used to maintain target serum albumin concentration, which helps to maintain effective volume by recruiting endogenous fluid^11^. This study found that iso-oncotic albumin was associated with better survival benefit in sepsis patients who suffer hypovolemia due to extravascular fluid loss caused by increased vascular permeability. However, hyperoncotic albumin achieved better survival possibilities in surgical patients, whose blood loss was caused by uncorrected blood loss. This indicated that iso-oncotic albumin helps with providing more volume for sepsis resuscitation, while hyperoncotic albumin is more beneficial for uncorrected blood loss patients with normal vascular permeability. Besides, iso-oncotic albumin in hypotonic solution was associated with higher mortality rate in traumatic brain injury patients, and greater fluid volume and hypotonic solution may further raise intracranial pressure, leading to a higher mortality [30].

### Hydroxyethyl starch (HES): L-HES and H-HES

HES of higher molecular weight has been retracted from the market, but the HES of lower molecular weight is still in use in daily practice, especially in surgical or trauma patients. However, this study found that L-HES was associated with the highest mortality rate in sepsis, surgical, and trauma patients, and a greater risk of acute kidney injury and greater transfusion volume was required during the resuscitation period. However, for traumatic brain injury patients, L-HES and saline, both hypertonic solutions, were associated with better survival than hypotonic solution, including iso-oncotic albumin and balanced crystalloid.

### Gelatin

Many review articles are opposed to gelatin use for fluid resuscitation due to the risk of anaphylaxis and acute kidney injury, but those opinions were based on animal studies, case series, or RCTs designed for other purposes [11, 31, 32]. Recent large RCTs reveal conflicting results, in that gelatin is associated with a nonsignificant, lower mortality than balanced crystalloids and saline^3^. Our sequential NMA demonstrated that the z-score trend for the difference between balanced crystalloids and gelatin has not yet exceeded the efficacy or futility boundary, indicating that the evidence was still insufficient (Fig. 2).

### Strengths and limitations

The present NMA analyzed all outcomes from previous RCTs, especially on the fluid resuscitation volume, which has never been considered in previous meta-analyses. This study also analyzed seven fluid types and patients’ conditions separately and demonstrated that the benefit or harmful effects of the fluid types were largely dependent on patients’ conditions. We present results from NMA followed by those from sequential NMA, in which the dynamic updates of the effect size help to corroborate the NMA results and estimate evidence uncertainty by depicting the trend and making allowance for multiple testing. Our NMA also has some limitations: first, in sepsis trials (sample size [*n*] = 14,659), the evidence was adequate between balanced crystalloids and saline, L-HES, and albumin, but insufficient between balanced crystalloids and gelatin. The confidence rating was low in surgical (*n* = 3871) and traumatic trials (*n* = 5076) because the sample size was insufficient and confidence intervals were wide. The confidence rating was very low for traumatic brain injury trials (*n* = 1970) because the direct and indirect evidence was inconsistent and sample sizes were insufficient. Secondly, the benefit or harm of gelatin could not be determined from the current evidence. Acute kidney injury was ranked best for gelatin in sepsis patients (only one trial) but was worse in surgical patients (only two trials). Survival benefit was also inconsistent between sepsis and surgical patients (Table 1). As very few trials included gelatin, the evidence on gelatin should be interpreted with cautions. Third, blood transfusion thresholds are unclear and largely dependent on physician decision in the included trials. In Additional file 1: appendix 8.1.5 (appendix pp. 74–75), we listed the blood transfusion volume and number of bleeding events requiring transfusion. Finally, the amount of investigation fluid was often very limited in many trials, and large volumes of these resuscitation fluids have not been well investigated. Some undetected adverse events may occur if larger volumes are used.

**Table 1:** Characteristics of the fluids assessed and qualitative summary from this network meta-analysis

Components	Plasma	Balanced crystalloid	Saline	Albumin (Iso-/Hyperoncotic)	L-HES	Gelatin
Osmolarity (mOsm/kg)	291	Hypotonic (254–273)	Isotonic (286)	Hypotonic (4%, 260; 5%, 250; 20%, 200; 25%, 250)	Isotonic to Hypertonic (283–304)	Isotonic to Hypertonic (274–301)
Na/Cl (mmol/l)	140/103	130–140/98–111	154/154	130–160/128–130	137–154/110–154	145–154/120–145
K/Ca (mmol/l)	40/4	4–5/2–2.7	0/0	< 2/0	0–4/0–2.5	0–5.1/0–6.25

Conditions	Outcome	Balanced crystalloid	Saline	Albumin	L-HES	Gelatin
Sepsis	NMA results	Lowest mortality Lowest acute kidney injury Lowest transfusion volume More fluid volume required	Higher mortality More fluid volume required	Lower mortality (Iso-oncotic) Least fluid volume required	Highest mortality More acute kidney injury More transfusion volume	
	Comments	Fluid of choice for sepsis	Not favored for sepsis	Iso-oncotic albumin for sepsis patients with risk of fluid overload	Not favored for sepsis	Require further trials
Surgery	NMA results	Most fluid volume required Lower acute kidney injury	More fluid volume required More blood transfusion volume	Lower mortality (Hyper-oncotic) Less fluid volume required Less acute kidney injury Less blood transfusion volume	Highest mortality Less fluid volume required	Less fluid volume required
	Comments	More favored for surgery	Less favored for surgery	Favored for surgery	Not favored for surgery	Require further trials
Trauma	Mortality	Lower mortality Less acute kidney injury Less transfusion volume More fluid volume required	Lower mortality More acute kidney injury More transfusion volume	Higher mortality Less acute kidney injury Less transfusion volume	Higher mortality	
	Comments	More favored for trauma	Damage control resuscitation. May consider blood products for resuscitation			
Traumatic brain injury (TBI)	Mortality	Higher mortality	Lower mortality	Highest mortality (Iso-oncotic)	Lowest mortality	
	Comments	Hypotonic solution was not suggested for TBI	Favored for TBI	Iso-oncotic albumin with hypotonic solution was not favored for TBI	May consider for TBI	Require further trials

## Conclusions

Among sepsis and surgical patients, balanced crystalloids and albumin attained lower mortality rates, lower risks of acute kidney injury, and less red blood transfusion volume than did saline and L-HES. Balanced crystalloids required the greatest fluid resuscitation volume than all the other fluid types. In traumatic brain injury patients, saline and L-HES showed better mortality rates than hypotonic solutions, including iso-oncotic albumin and balanced crystalloids.

## Supplementary Information

**Additional file 1**. Appendix.

## Data Availability

All data generated or analyzed during this study are included in this published article and its supplementary files.
