# Supplementary material for: Resuscitation fluid types in sepsis, surgical, and trauma patients: a systematic review and sequential network meta-analyses
Source: Crit Care. 2020 Dec 14;24:693. doi: 10.1186/s13054-020-03419-y (PMC7734863; doi:10.1186/s13054-020-03419-y)
Supplement: Supplementary file 1 — Additional file 1. Appendix. [file 13054_2020_3419_MOESM1_ESM.docx]

**Appendix**

**Table of content**

[Appendix 1. Systematic reviews and meta-analysis published on fluid resuscitation in critically-ill patients 5](#_Toc53220792)

[Appendix 2 : PRISMA checklist 8](#_Toc53220793)

[Appendix 3: Protocol and Search strategies 14](#_Toc53220794)

[3.1. Review eligibility criteria 14](#_Toc53220795)

[3.2. Search vocabulary 15](#_Toc53220796)

[Appendix 4: Excluded studies and reasons 17](#_Toc53220797)

[Appendix 5: List of included studies 21](#_Toc53220798)

[5.1. Sepsis patients 21](#_Toc53220799)

[5.1.1. Study group and primary outcome 21](#_Toc53220800)

[5.1.2. Study population and enrollment criteria 22](#_Toc53220801)

[5.1.3. Baseline characteristics 26](#_Toc53220802)

[5.1.4. Resuscitation goal and fluid volume 28](#_Toc53220803)

[5.2. Surgical patients 31](#_Toc53220804)

[5.2.1 Population, intervention, outcome 31](#_Toc53220805)

[5.2.2 Baseline characteristics, resuscitation goal and fluid volume 34](#_Toc53220806)

[5.3. Traumatic patients 36](#_Toc53220807)

[5.3.1 Population, intervention, outcome 36](#_Toc53220808)

[5.3.2 Baseline characteristics and fluid volume 38](#_Toc53220809)

[5.4. References 39](#_Toc53220810)

[Appendix 6: Assessment of transitivity 48](#_Toc53220811)

[6.1. Age 49](#_Toc53220812)

[6.2. Male percentage 50](#_Toc53220813)

[6.3. Sample size 51](#_Toc53220814)

[6.4. APACHE 52](#_Toc53220815)

[6.5. SAPS 53](#_Toc53220816)

[6.6. SOFA 54](#_Toc53220817)

[6.7. Mean arterial pressure 55](#_Toc53220818)

[6.8. Lactate level 56](#_Toc53220819)

[6.9. Vasopressor 57](#_Toc53220820)

[6.10. Source of sepsis from lung (pneumonia) 58](#_Toc53220821)

[6.11. Year 59](#_Toc53220822)

[Appendix 7: Risk of bias 60](#_Toc53220823)

[7.1. Sepsis patients 60](#_Toc53220824)

[7.1.1. Risk of bias assessment for the individual domains in sepsis trials 60](#_Toc53220825)

[7.1.2. Risk of bias assessment for the individual studies in sepsis trials 61](#_Toc53220826)

[7.1.3. Risk of bias notes for the individual studies in sepsis trials 62](#_Toc53220827)

[7.2 Surgical patients 64](#_Toc53220828)

[7.2.1. Risk of bias assessment for studies in surgical trials 64](#_Toc53220829)

[7.2.2. Risk of bias assessment for the individual studies in surgical trials 65](#_Toc53220830)

[7.2.3. Risk of bias notes for the individual studies in surgical trials 66](#_Toc53220831)

[7.3 Trauma patients 67](#_Toc53220832)

[7.3.1. Risk of bias assessment for studies in trauma trials 67](#_Toc53220833)

[7.3.2. Risk of bias assessment for the individual studies in trauma trials 68](#_Toc53220834)

[7.3.3. Risk of bias notes for the individual studies in trauma trials 69](#_Toc53220835)

[Appendix 8: Results 70](#_Toc53220836)

[8.1. Extracted outcome data in sepsis patients 70](#_Toc53220837)

[8.1.1. Mortality in sepsis patients 70](#_Toc53220838)

[8.1.2. Resuscitation fluid volume in sepsis patients 71](#_Toc53220839)

[8.1.3. No. of acute kidney injury in sepsis patients 72](#_Toc53220840)

[8.1.4. No. of renal replacement events in sepsis patients 73](#_Toc53220841)

[8.1.5. Blood transfusion volume in sepsis patients 74](#_Toc53220842)

[8.1.6. No. of bleeding events requiring transfusion in sepsis patients 75](#_Toc53220843)

[8.1.7. Allergic events in sepsis patients 76](#_Toc53220844)

[8.2. Extracted outcome data in surgical patients 77](#_Toc53220845)

[8.2.1. Mortality data in surgical patients 77](#_Toc53220846)

[8.2.2. Resuscitation fluid volume in surgical patients 78](#_Toc53220847)

[8.2.3. No. of acute kidney injury in surgical patients 79](#_Toc53220848)

[8.2.4. Blood transfusion volume in surgical patients 80](#_Toc53220849)

[8.3. Extracted outcome data in trauma patients 81](#_Toc53220850)

[8.3.1. Mortality in trauma patients 81](#_Toc53220851)

[8.3.2. Resuscitation fluid volume in trauma patients 82](#_Toc53220852)

[8.3.3. No. of acute kidney injury in trauma patients 83](#_Toc53220853)

[8.3.4. Blood transfusion volume in trauma patients 84](#_Toc53220854)

[8.4. Extracted outcome data in traumatic brain injury patients 85](#_Toc53220855)

[8.4.1. Mortality in traumatic brain injury patients 85](#_Toc53220856)

[Appendix 9: League table and Relative ranking 86](#_Toc53220857)

[9.1. Sepsis patients 86](#_Toc53220858)

[9.1.1. Mortality in sepsis patients 86](#_Toc53220859)

[9.1.2. Resuscitation fluid volume in sepsis patients 88](#_Toc53220860)

[9.1.3. Acute kidney injury in sepsis patients 90](#_Toc53220861)

[9.1.4. Blood transfusion volume in sepsis patients 92](#_Toc53220862)

[9.2. Relative ranking probability in surgical patients 94](#_Toc53220863)

[9.2.1. Mortality in surgical patients 94](#_Toc53220864)

[9.2.2. Resuscitation fluid volume in surgical patients 96](#_Toc53220865)

[9.2.3. Acute kidney injury in surgical patients 98](#_Toc53220866)

[9.2.4. Blood transfusion volume in surgical patients 100](#_Toc53220867)

[9.3. Relative ranking probability in trauma patients 102](#_Toc53220868)

[9.3.1. Mortality in trauma patients 102](#_Toc53220869)

[9.3.2. Fluid resuscitation volume in trauma patients 104](#_Toc53220870)

[9.3.3. Adverse renal events in trauma patients 106](#_Toc53220871)

[9.3.4. Blood transfusion volume in trauma patients 108](#_Toc53220872)

[9.3.5. Mortality in traumatic brain injury patients 110](#_Toc53220873)

[9.4. Interval plot for surgical and trauma patients 112](#_Toc53220874)

[Appendix 10: Publication bias 115](#_Toc53220875)

[10.1. Publication bias in sepsis patients 115](#_Toc53220876)

[10.1.1 Mortality in sepsis patients 115](#_Toc53220877)

[10.1.2. Resuscitation fluid volume in sepsis patients 116](#_Toc53220878)

[10.1.3. Acute kidney injury in sepsis patients 117](#_Toc53220879)

[10.1.4. Blood transfusion volume in sepsis patients 118](#_Toc53220880)

[10.2. Publication bias in surgical patients 119](#_Toc53220881)

[10.2.1. Mortality in surgical patients 119](#_Toc53220882)

[10.2.2. Resuscitation fluid volume in surgical patients 120](#_Toc53220883)

[10.2.3. Acute kidney injury in surgical patients 121](#_Toc53220884)

[10.2.4. Blood transfusion among in surgical patients 122](#_Toc53220885)

[10.3. Publication bias in trauma patients 123](#_Toc53220886)

[10.3.1.Mortality in trauma patients 123](#_Toc53220887)

[10.3.2. Mortality in traumatic brain injury patients 124](#_Toc53220888)

[Appendix 11: Inconsistency 125](#_Toc53220889)

[11.1. Inconsistency in sepsis patients 126](#_Toc53220890)

[11.1.1. Mortality in sepsis patients 126](#_Toc53220891)

[11.1.2. Resuscitation fluid volume in sepsis patients 128](#_Toc53220892)

[11.1.4. Blood transfusion among in sepsis patients 130](#_Toc53220893)

[11.2. Inconsistency in surgical patients 131](#_Toc53220894)

[11.2.1. Mortality in surgical patients 131](#_Toc53220895)

[11.2.2. Resuscitation fluid volume in surgical patients 133](#_Toc53220896)

[11.2.3. Acute kidney injury in surgical patients 134](#_Toc53220897)

[11.2.4. Blood transfusion among in surgical patients 136](#_Toc53220898)

[11.3. Inconsistency in traumatic patients 138](#_Toc53220899)

[11.3.1. Mortality in traumatic patients 138](#_Toc53220900)

[Appendix 12: Meta-regression 139](#_Toc53220901)

[12.1. SUCRA and mean ranks changes before and after model adjust in sepsis trials 139](#_Toc53220902)

[12.2. Significance for meta-regression model in sepsis trials 139](#_Toc53220903)

[Appendix 13: Grading the evidence using CINeMA web application 140](#_Toc53220904)

[13.1. Sepsis patients 140](#_Toc53220905)

[13.1.1 Confidence rating in sepsis trails for mortality 140](#_Toc53220906)

[13.1.2 Confidence rating in sepsis trails for fluid resuscitation volume 141](#_Toc53220907)

[13.1.3 Confidence rating in sepsis trails for acute kidney injury 142](#_Toc53220908)

[13.1.4 Confidence rating in sepsis trails for blood transfusion volume 143](#_Toc53220909)

[13.2. Surgical patients 144](#_Toc53220910)

[13.2.1 Confidence rating in surgical trails for mortality 144](#_Toc53220911)

[13.2.2 Confidence rating in surgical trails for fluid resuscitation volume 145](#_Toc53220912)

[13.2.3 Confidence rating in surgical trails for adverse renal events 146](#_Toc53220913)

[13.2.4 Confidence rating in surgical trails for blood transfusion volume 147](#_Toc53220914)

[13.3. Trauma patients 148](#_Toc53220915)

[13.4. Traumatic brain injury patients 150](#_Toc53220916)

[Appendix 14: Sensitivity analysis 151](#_Toc53220917)

[14.1. Exclusion with largest trial (The SMART randomized trial) 151](#_Toc53220918)

[14.1.1. Mortality 151](#_Toc53220919)

[14.1.2. Fluid resuscitation volume 152](#_Toc53220920)

[14.1.3. Acute kidney injury 153](#_Toc53220921)

[14.1.4. Blood cell transfusion volume 154](#_Toc53220922)

[14.2. Inclusion with pilot study (The SALT Randomized Trial) 155](#_Toc53220923)

[14.2.1. Mortality 155](#_Toc53220924)

[14.2.2. Acute kidney injury 156](#_Toc53220925)

#

# Appendix 1. Systematic reviews and meta-analysis published on fluid resuscitation in critically-ill patients

eTable 1. Systematic reviews and meta-analysis published on fluid resuscitation in critically-ill patients

| **Author.**  **Journal, Year** | **No. of trials**  **No. of cases** | **Patients** | **Crystalloids**  **(No. of trials)** | **Colloids**  **(No. of trials)** | **Outcome** | **Weakness** | | |
| --- | --- | --- | --- | --- | --- | --- | --- | --- |
|  |  |  |  |  |  | **Comments** | **Different**  **population** | **Different fluid** |
| Perel et al.  Cochrane, 2013 ^1^ | 56,  20407 | All critical ill patients | Isotonic crystalloid (67)  Hypertonic crystalloid (14) | Albumin (24), HES (25),  Gelatin (11), Dextran (9) | Mortality | Combined different patient populations  Combined H-HES and L-HES  Combined different isotonic crystalloid | V | V |
| Zarychanski et al.  JAMA, 2013 ^2^ | 38,  10880 | All critical ill patients | - | HES vs. other colloids | Mortality, Kidney,  Transfusion, Allergy | Combined different patient populations  Combined all HES with different molecular weight  Combined all crystalloid and colloid other than HES | V | V |
| Krajewski et al.  BJS, 2014 ^3^ | 21,  6253 | All critical ill patients | BS (21),  Saline (21) | - | Mortality, Acidosis,  Bleeding | Combined medical, surgical, trauma, burn and children  Including 2 large retrospective trial | V |  |
| Qureshi et al.  BJS, 2016 ^4^ | 59,  16889 | All critical ill patients | All crystalloid (41) | H-HES (25), L-HES (15), Dextran (13), Gelatin (15), | Mortality, Kidney | Combined different patient populations  Combined different crystalloid | V | V |
| Moeller et al.  JCC, 2016 ^5^ | 30,  3629 | All critical ill patients | All crystalloid (21) | Gelatin (30), other colloids (23) | Mortality, Kidney,  Anaphylaxis, Bleeding | Combined different patient populations,  almost half cases were the child or preterm babies  Combined different crystalloid | V | V |
| Rochwerg et al. AIM, 2014 ^6^  ICM, 2015 ^7^ | 14,  18916 | Sepsis | BS (3), Saline (9) | Albumin (4), H-HES (6),  L-HES (6), Gelatin (1), | Mortality | 4 node analysis combined different crystalloids  Combined different albumin  Only 14 trials were included |  | V |
|  | 10,  6664 | Sepsis | BS (2), Saline (6) | Albumin (1), H-HES (3),  L-HES (5), Gelatin (1), | RRT | Other important side effects were not included |  |  |
| Patel et al.  BMJ, 2014 ^8^ | 16,  4190 | Sepsis | - | Albumin (10), other colloids (10) | Mortality | Combined different colloids |  | V |
| Patel et al.  BMJ, 2014 ^8^ | 7,  3878 | Sepsis | Mixed (7) | Albumin (7) | Mortality | Combined different crystalloids |  | V |
| Jiang et al. PlosOne, 2014 ^9^ | 15,  6998 | Sepsis | Mixed (10) | Albumin (15), other colloids (5) | Mortality | Combined different crystalloids |  | V |
| Xu et al.  CC, 2014 ^10^ | 5,  5838 | Sepsis | Mixed (5) | Albumin (5) | Mortality | Combined different crystalloids |  | V |
| Neto et al.  JCC, 2016 ^11^ | 10,  4624 | Sepsis | Mixed (10) | HES (10) | Mortality, RRT, BT | Combined different crystalloids |  | V |
| Cortes et al.  BJA, 2014 ^12^ | 5060,  28 | Surgery | BS (28), Saline (28) | - | Electrolytes,  organ functions | Saline more acidosis, bleeding, |  |  |
| Burdett et al.  Cochrane, 2017 ^13^ | 706,  13 | Surgery | BS (13), Saline (13) | - | Mortality, pH and  transfusion volume | Few studies, no enough power to  detect the possible difference |  |  |
| Van Der Linden et al. Anesth Analg, 2013 ^14^ | 4529,  59 | Surgery | Mixed | HES, other colloids | Mortality, AKI, bleeding | Combined different crystalloids |  | V |
| Martin et al. Anesthesiology, 2013 ^15^ | 1230,  17 | Surgery | Mixed | HES, other colloids | Mortality, AKI | Combined different crystalloids |  | V |

*BS, balanced solution, including BC or Plasma-Lyte; HES, hydroxyethyl starch; AKI, acute kidney injury; BT, blood transfusion

# Appendix 2 : PRISMA checklist

eTable 2. PRISMA checklist

| **Section/Topic** | **Item #** | **Checklist Item ^16^** | **Reported on Page #** |
| --- | --- | --- | --- |
| **TITLE** | | | |
| Title | 1 | Identify the report as a systematic review *incorporating a network meta-analysis (or related form of meta-analysis).* | **1, Title section** |
| **ABSTRACT** | | | |
| Structured summary | 2 | Provide a structured summary including, as applicable: **Background:** main objectives; **Methods:** data sources; study eligibility criteria, participants, and interventions; study appraisal; and *synthesis methods, such as network meta-analysis.* **Results:** number of studies and participants identified; summary estimates with corresponding confidence/credible intervals; *treatment rankings may also be discussed. Authors may choose to summarize pairwise comparisons against a chosen treatment included in their analyses for brevity.***Discussion/Conclusions:** limitations; conclusions and implications of findings. **Other:** primary source of funding; systematic review registration number with registry name. | **4, Abstract section** |
| **INTRODUCTION** | | | |
| Rationale | 3 | Describe the rationale for the review in the context of what is already known*, including mention of why a network meta-analysis has been conducted.* | **5, Introduction (2^nd^ paragraph)** |
| Objectives | 4 | Provide an explicit statement of questions being addressed, with reference to participants, interventions, comparisons, outcomes, and study design (PICOS). | **5, Introduction (3^rd^ paragraph);**  **Appendix 3** |
| **METHODS** | | | |
| Protocol and registration | 5 | Indicate whether a review protocol exists: **PROSPERO register：CRD42018115641** | **7, Method (1^st^ paragraph)** |
| Eligibility criteria | 6 | Specify study characteristics (e.g., PICOS, length of follow-up) and report characteristics (e.g., years considered, language, publication status) used as criteria for eligibility, giving rationale. *Clearly describe eligible treatments included in the treatment network, and note whether any have been clustered or merged into the same node (with justification).* | **8, Method (2^nd^ paragraph);** |

| Information sources | 7 | Describe all information sources (e.g., databases with dates of coverage, contact with study authors to identify additional studies) in the search and date last searched. | **8, Method (2^nd^ paragraph);** |
| --- | --- | --- | --- |
| Search | 8 | Present full electronic search strategy for at least one database, including any limits used, such that it could be repeated. | **8, Method (2^nd^ paragraph); Appendix 3, 4** |
| Study selection | 9 | State the process for selecting studies (i.e., screening, eligibility, included in systematic review, and, if applicable, included in the meta-analysis). | **8, Method (2^nd^ paragraph);**  **Appendix 4** |
| Data collection process | 10 | Describe method of data extraction from reports (e.g., piloted forms, independently, in duplicate) and any processes for obtaining and confirming data from investigators. | **8, Method (2^nd^ paragraph)** |
| Data items | 11 | List and define all variables for which data were sought (e.g., PICOS, funding sources) and any assumptions and simplifications made. | **8, Method (2^nd^ paragraph)** |
| **Geometry of the network** | **S1** | Describe methods used to explore the geometry of the treatment network under study and potential biases related to it. This should include how the evidence base has been graphically summarized for presentation, and what characteristics were compiled and used to describe the evidence base to readers. | **9, Method (3^rd^ paragraph);**  **Figure 1** |
| Risk of bias within individual studies | 12 | Describe methods used for assessing risk of bias of individual studies (including specification of whether this was done at the study or outcome level), and how this information is to be used in any data synthesis. | **8-9, Method (2^nd^ paragraph** |
| Summary measures | 13 | State the principal summary measures (e.g., risk ratio, difference in means). *Also describe the use of additional summary measures assessed, such as treatment rankings and surface under the cumulative ranking curve (SUCRA) values, as well as modified approaches used to present summary findings from meta-analyses.* | **8, Method (2^nd^ paragraph** |
| Planned methods of analysis | 14 | Describe the methods of handling data and combining results of studies for each network meta-analysis. | **9, Method (3^rd^ paragraph)** |
| **Assessment of Inconsistency** | **S2** | Describe the statistical methods used to evaluate the agreement of direct and indirect evidence in the treatment network(s) studied. Describe efforts taken to address its presence when found. | **9, Method (3^rd^ paragraph)** |
| Risk of bias across studies | 15 | Specify any assessment of risk of bias that may affect the cumulative evidence (e.g., publication bias, selective reporting within studies). | **9, Method (3^rd^ paragraph)** |
| Additional analyses | 16 | Describe methods of additional analyses if done, indicating which were pre-specified. | **9, Method (3^rd^ paragraph)** |
| **RESULTS†** | | | |
| Study selection | 17 | Give numbers of studies screened, assessed for eligibility, and included in the review, with reasons for exclusions at each stage, ideally with a flow diagram. | **5, Results (1^st^ paragraph); eFigure 1, eTable 6** |
| **Presentation of network structure** | **S3** | Provide a network graph of the included studies to enable visualization of the geometry of the treatment network. | **Figure 1, eFigure 2** |
| **Summary of network geometry** | **S4** | Provide a brief overview of characteristics of the treatment network. This may include commentary on the abundance of trials and randomized patients for the different interventions and pairwise comparisons in the network, gaps of evidence in the treatment network, and potential biases reflected by the network structure. | **10, Results (1^st^ paragraph).** |
| Study characteristics | 18 | For each study, present characteristics for which data were extracted (e.g., study size, PICOS, follow-up period) and provide the citations. | **10, Results (2^nd^ paragraph);  eTable 7A-7D** |
| Risk of bias within studies | 19 | Present data on risk of bias of each study and, if available, any outcome level assessment. | **10, Results (2^nd^ paragraph);  eTable 8** |
| Results of individual studies | 20 | For all outcomes considered (benefits or harms), present, for each study: 1) simple summary data for each intervention group, and 2) effect estimates and confidence intervals. | **10, Results (3^rd^ paragraph);  eTable 9A-9F** |
| Synthesis of results | 21 | Present results of each meta-analysis done, including confidence/credible intervals. If additional summary measures were explored (such as treatment rankings), these should also be presented. | **10, Results (3^rd^ paragraph);  Figure 2** |
| **Exploration for inconsistency** | **S5** | Describe results from investigations of inconsistency. This may include such information as measures of model fit to compare consistency and inconsistency models, *P* values from statistical tests, or summary of inconsistency estimates from different parts of the treatment network. | **10, Results (4^th^ paragraph);** |
| Risk of bias across studies | 22 | Present results of any assessment of risk of bias across studies for the evidence base being studied. | **10, Results (4^th^ paragraph);** |
| Results of additional analyses | 23 | Give results of additional analyses, if done (e.g., sensitivity or subgroup analyses, meta-regression analyses*, alternative network geometries studied, alternative choice of prior distributions for Bayesian analyses,* and so forth). | **11, Results (4^th^ paragraph);  Figure 3** |
| **DISCUSSION** |  |  |  |
| Summary of evidence | 24 | Summarize the main findings, including the strength of evidence for each main outcome; consider their relevance to key groups (e.g., healthcare providers, users, and policy-makers). | **17** |
| Limitations | 25 | Discuss limitations at study and outcome level (e.g., risk of bias), and at review level (e.g., incomplete retrieval of identified research, reporting bias). *Comment on the validity of the assumptions, such as transitivity and consistency. Comment on any concerns regarding network geometry (e.g., avoidance of certain comparisons).* | **17** |
| Conclusions | 26 | Provide a general interpretation of the results in the context of other evidence, and implications for future research. | **17** |
| **FUNDING** | | | |
| Funding | 27 | Describe sources of funding for the systematic review and other support (e.g., supply of data); role of funders for the systematic review. This should also include information regarding whether funding has been received from manufacturers of treatments in the network and/or whether some of the authors are content experts with professional conflicts of interest that could affect use of treatments in the network. | **23** |

PICOS = population, intervention, comparators, outcomes, study design.

* Text in italics indicateS wording specific to reporting of network meta-analyses that has been added to guidance from the PRISMA statement.

† Authors may wish to plan for use of appendices to present all relevant information in full detail for items in this section.

Reference. For Appendix 1 and 2

1. Perel P, Roberts I, Ker K. Colloids versus crystalloids for fluid resuscitation in critically ill patients. *The Cochrane database of systematic reviews.* 2013(2):Cd000567.

2. Zarychanski R, Abou-Setta AM, Turgeon AF, et al. Association of hydroxyethyl starch administration with mortality and acute kidney injury in critically ill patients requiring volume resuscitation: A systematic review and meta-analysis. *JAMA - Journal of the American Medical Association.* 2013;309(7):678-688.

3. Krajewski ML, Raghunathan K, Paluszkiewicz SM, Schermer CR, Shaw AD. Meta-analysis of high- versus low-chloride content in perioperative and critical care fluid resuscitation. *Br J Surg.* 2015;102(1):24-36.

4. Qureshi SH, Rizvi SI, Patel NN, Murphy GJ. Meta-analysis of colloids versus crystalloids in critically ill, trauma and surgical patients. *Br J Surg.* 2016;103(1):14-26.

5. Moeller C, Fleischmann C, Thomas-Rueddel D, et al. How safe is gelatin? A systematic review and meta-analysis of gelatin-containing plasma expanders vs crystalloids and albumin. *J Crit Care.* 2016;35:75-83.

6. Rochwerg B, Alhazzani W, Sindi A, et al. Fluid resuscitation in sepsis: a systematic review and network meta-analysis. *Ann Intern Med.* 2014;161(5):347-355.

7. Rochwerg B, Alhazzani W, Gibson A, et al. Fluid type and the use of renal replacement therapy in sepsis: a systematic review and network meta-analysis. *Intensive Care Med.* 2015;41(9):1561-1571.

8. Patel A, Laffan MA, Waheed U, Brett SJ. Randomised trials of human albumin for adults with sepsis: systematic review and meta-analysis with trial sequential analysis of all-cause mortality. *Bmj.* 2014;349:g4561.

9. Jiang L, Jiang S, Zhang M, Zheng Z, Ma Y. Albumin versus other fluids for fluid resuscitation in patients with sepsis: A meta-analysis. *PLoS ONE.* 2014;9(12).

10. Xu JY, Chen QH, Xie JF, et al. Comparison of the effects of albumin and crystalloid on mortality in adult patients with severe sepsis and septic shock: a meta-analysis of randomized clinical trials. *Crit Care.* 2014;18(6):702.

11. Serpa Neto A, Veelo DP, Peireira VG, et al. Fluid resuscitation with hydroxyethyl starches in patients with sepsis is associated with an increased incidence of acute kidney injury and use of renal replacement therapy: a systematic review and meta-analysis of the literature. *J Crit Care.* 2014;29(1):185.e181-187.

12. Orbegozo Cortes D, Rayo Bonor A, Vincent JL. Isotonic crystalloid solutions: a structured review of the literature. *Br J Anaesth.* 2014;112(6):968-981.

13. Bampoe S, Odor PM, Dushianthan A, et al. Perioperative administration of buffered versus non-buffered crystalloid intravenous fluid to improve outcomes following adult surgical procedures. *The Cochrane database of systematic reviews.* 2017;9:CD004089.

14. Van Der Linden P, James M, Mythen M, Weiskopf RB. Safety of modern starches used during surgery. *Anesth Analg.* 2013;116(1):35-48.

15. Martin C, Jacob M, Vicaut E, Guidet B, Van Aken H, Kurz A. Effect of waxy maize-derived hydroxyethyl starch 130/0.4 on renal function in surgical patients. *Anesthesiology.* 2013;118(2):387-394.

16. Hutton B, Salanti G, Caldwell DM, et al. The PRISMA extension statement for reporting of systematic reviews incorporating network meta-analyses of health care interventions: checklist and explanations. *Ann Intern Med.* 2015;162(11):777-784.

17. Futier E, Garot M, Godet T, et al. Effect of Hydroxyethyl Starch vs Saline for Volume Replacement Therapy on Death or Postoperative Complications Among High-Risk Patients Undergoing Major Abdominal Surgery: The FLASH Randomized Clinical Trial. *JAMA.* 2020;323(3):225-236.

# Appendix 3: Protocol and Search strategies

Protocol as published in PROSPERO (Registration number: CRD42018115641

## 3.1. Review eligibility criteria

eTable 3.1. PICOS, Inclusion and exclusion criteria

| Patient | Acute ill patients requiring fluid resuscitation, including sepsis, trauma, surgical and burn patients |
| --- | --- |
| Intervention | Crystalloid solutions, including high chloride solution (e.g. Saline) or low chloride solutions (e.g. Ringer’s lactate, Ringer’s acetate, or Plasmalytes) |
| Comparator | Colloid solutions, including albumin (4%, 5%, 20% or 25%), high HES (MW≧200), low HES (MW≦130), dextran, or gelatin. |
| Outcomes | 1. Incidence of mortality 2. Incidence of patients with renal injury (use of renal replacement therapy) 3. Incidence of patients with major or minor bleeding events |
| Study design | Prospective randomized controlled trials |
| Inclusion criteria | 1. Randomized controlled trial. 2. Participant’s age≧18 years. 3. Indication for acute fluid resuscitation (e.g. hypovolemia or hypotension during sepsis, trauma, surgery or burn) 4. Allocation to resuscitation fluid with one with another among above fluid types |
| Exclusion criteria | 1. Observational study designs, cross-over trial, or single arm study 2. No specified patients clinical conditions for volume resuscitation 3. Comparator used is whole blood or blood products |

## 3.2. Search vocabulary

| **Pubmed** | ((RCT) OR (randomized controlled trial)) AND ((fluid therapy) OR (fluid replacement) OR (fluid resuscitation) OR (volume replacement) OR (volume expansion) OR (crystalloid) OR (colloid) OR (HES) OR (hydroxyethyl starch) OR (hetastarch) OR (hespan) OR (alba) OR (albumin) OR (gelatin) OR (dextran) OR (volulyte) OR (haemaccel) OR (ringers lactate) OR (hartmann’s solution) OR (plasmalyte) OR (normosol) OR (normal saline) OR (0.9% saline) OR (pentastarch)) AND ((AKI) OR (acute kidney injury) OR (acute renal injury) OR (renal failure) OR (renal insult) OR (CVVH) OR (haemodialysis) OR (hemofiltration) OR (dialysis) OR (renal replacement therapy) OR (morbidity) OR (organ dysfunction) OR (all-cause mortality) OR (mortality) OR (death) OR (bleeding) OR (transfusion)). |
| --- | --- |
| **EMBASE, PICO search** | ('sepsis'/exp OR 'abdominal sepsis' OR 'focal sepsis' OR 'intraabdominal sepsis' OR 'sepsis' OR 'sepsis syndrome' OR 'septic disease' OR 'injury'/exp OR 'surgery'/exp OR 'burn'/exp) AND ('crystalloid'/exp OR 'crystalloid' OR 'crystalloid formation' OR 'crystalloid solution' OR 'crystalloid solutions' OR 'colloid'/exp OR '113m jiv fe hydroxide macrocolloid' OR 'colloid' OR 'colloid solution' OR 'colloid substance' OR 'colloidal solution' OR 'colloids' OR 'hydrophilic colloid' OR 'solution, colloid' OR 'hetastarch'/exp OR '2 hydroxyethyl starch' OR 'amidolite' OR 'amylopectin, hydroxyethyl' OR 'asl 607' OR 'asl-607' OR 'asl607' OR 'elohaest' OR 'elohast' OR 'elohes' OR 'equihes' OR 'haes steril' OR 'haes-steril' OR 'heafusine' OR 'hemoes' OR 'hemohes' OR 'hes 130/0.4' OR 'hes steril' OR 'hespan' OR 'hespander' OR 'hesra' OR 'hetastarch' OR 'hetastarch 120' OR 'hetastarch 200' OR 'hetastarch 40' OR 'hextend' OR 'hydroxy ethyl starch' OR 'hydroxyethyl starch' OR 'hydroxyethyl starch 130/0.4' OR 'hydroxyethyl starch 60' OR 'hydroxyethylamylopectin' OR 'hydroxyethylstarch' OR 'hyperhaes' OR 'hyperhes' OR 'infohes' OR 'infukoll hes' OR 'isohes' OR 'isovol' OR 'ketastarch' OR 'onkohaes' OR 'onkohas' OR 'pentafraction' OR 'pentaspan' OR 'pentastarch' OR 'plasmafusin' OR 'plasmasteril' OR 'plasmohes' OR 'polyhydroxyamylopectin' OR 'refortan' OR 'refortan plus' OR 'restorvol' OR 'stabisol' OR 'starch, hydroxyethyl' OR 'tetraspan (drug)' OR 'venofundin' OR 'venohes' OR 'vitafusal' OR 'vitakoll' OR 'volex' OR 'voluforte' OR 'volulyte' OR 'voluven' OR 'vonten' OR 'albumin'/exp OR 'albumen' OR 'albumin' OR 'albumin secretion' OR 'albumin variant' OR 'liquid albumin' OR 'gelatin'/exp OR 'byco c' OR 'emagel' OR 'esma spreng' OR 'esmaspreng' OR 'fluid gelatin' OR 'gel film' OR 'gelafusal' OR 'gelatin' OR 'gelatin capsule' OR 'gelatin hydrolysate' OR 'gelatin medium' OR 'gelatin polymer' OR 'gelatin polymerizate' OR 'gelatin solution' OR 'gelatine' OR 'gelfilm' OR 'gelpart' OR 'high molecular gelatin' OR 'high molecular weight gelatin' OR 'hydroxypolygelatine' OR 'low molecular weight gelatin' OR 'modified fluid gelatin' OR 'pharmagel a' OR 'puragel' OR 'dextran'/exp OR 'alpha 1, 6 glucan' OR 'cyanogen bromide activated dextran' OR 'dextran' OR 'dextran 40 injection' OR 'dextran 45' OR 'dextran 70 in 0.9% sodium chloride' OR 'dextran 70 injection' OR 'dextran 75 in 0.9% sodium chloride' OR 'dextran k 300000' OR 'dextran k 40000' OR 'dextran k 500000' OR 'dextran k 70000' OR 'dextran plus dextrose' OR 'dextran plus sodium chloride' OR 'dextran-hm' OR 'dextrane' OR 'dextrans' OR 'dextraven' OR 'dextraven 150' OR 'dextril 70' OR 'dialens' OR 'expandex' OR 'gentran' OR 'hemodex' OR 'high molecular weight dextran' OR 'infucoll' OR 'infukoll' OR 'intradex' OR 'lm dextran' OR 'lm dextran l' OR 'lmd with 0.9% sodium chloride' OR 'lmd with 5% dextrose' OR 'lmw dextran' OR 'longasteril' OR 'low molecular dextran' OR 'low molecular weight dextran' OR 'macrodex' OR 'macrodex 500' OR 'macrodex 6%' OR 'macrose' OR 'neoplasmafusin' OR 'neoschiwadex' OR 'oncovertin' OR 'onkotin' OR 'onkovertin' OR 'pharmodex' OR 'plasmaclair' OR 'plavolex' OR 'polyglucin' OR 'polyglucine' OR 'polyglucinum' OR 'polyglusol' OR 'polyglusole' OR 'promit' OR 'promiten' OR 'pvtd' OR 'rheomacrodex' OR 'rheomacrodex 10%' OR 'sinkol' OR 'sodium meta periodate activated dextran' OR 'ringer lactate solution'/exp OR 'ringer lactate solution' OR 'acetated ringer solution' OR 'lactate ringer solution' OR 'lactated ringer solution' OR 'lactated ringer`s' OR 'lactated ringers' OR 'lactated ringers irrigation' OR 'lactated ringer`s solution' OR 'ringer lactate' OR 'ringer lactated solution' OR 'ringer saline solution' OR 'ringer solution lactated' OR 'ringer solution, lactated' OR 'sodium chloride'/exp OR 'alcathion' OR 'bacteriostatic sodium chloride 0.9%' OR 'broncho saline' OR 'hypertonic lactated saline solution' OR 'hypertonic saline' OR 'hypertonic saline bath' OR 'hypertonic sodium chloride' OR 'hypertonic sodium chloride solution' OR 'hypotonic sodium chloride' OR 'hypotonic sodium chloride solution' OR 'natrium chloride' OR 'natural saline' OR 'normal saline' OR 'physiological saline' OR 'physiological solution' OR 'saline' OR 'saline solution, hypertonic' OR 'salt' OR 'sodium chloride' OR 'sodium chloride 0.45%' OR 'sodium chloride 0.9%' OR 'sodium chloride 23.4%' OR 'sodium chloride 3%' OR 'sodium chloride 5%' OR 'sodium chloride solution' OR 'sodiumchloride' OR 'table salt') AND ('mortality'/exp OR 'excess mortality' OR 'mortality' OR 'mortality model' OR 'kidney failure'/exp OR 'kidney failure' OR 'kidney insufficiency' OR 'maternal kidney failure' OR 'renal failure' OR 'renal insufficiency' OR 'terminal kidney failure' OR 'bleeding'/exp OR 'abnormal bleeding' OR 'bleeding' OR 'bleeding complication' OR 'blood loss' OR 'haemorrhage' OR 'hemorrhage' OR 'blood transfusion'/exp OR 'blood exchange' OR 'blood infusion' OR 'blood replacement' OR 'blood retransfusion' OR 'blood transfusion' OR 'transfusion blood' OR 'transfusion therapy' OR 'transfusion, blood') AND ('randomized controlled trial'/exp OR 'controlled trial, randomized' OR 'randomised controlled study' OR 'randomised controlled trial' OR 'randomized controlled study' OR 'randomized controlled trial' OR 'trial, randomized controlled') |
| **CENTRAL** | ((fluid resuscitation) OR (volume replacement) OR (crystalloid) OR (colloid) OR (hydroxyethyl starch) OR (albumin) OR (gelatin) OR (dextran) OR (ringers lactate) OR (normal saline) OR (0.9% saline) OR (pentastarch)) AND ( (acute kidney injury) OR (acute renal injury) OR (renal failure) OR (haemodialysis) OR (renal replacement therapy) OR (morbidity) OR (organ dysfunction) OR (all-cause mortality) OR (mortality) OR (death) OR (bleeding) OR (transfusion)). |

# Appendix 4: Excluded studies and reasons

- **Systematic review or meta-analysis articles : 16 articles listed in eTable 1**
- **Retracted paper**
  - Boldt J, Heesen M, Müller M, Pabsdorf M, Hempelmann G. The effects of albumin versus hydroxyethyl starch solution on cardiorespiratory and circulatory variables in critically ill patients. Anesth Analg. 1996 Aug;83(2):254-61. Retraction in: Shafer SL. Anesth Analg. 2014 Nov;119(5):1225.
  - Boldt J, Müller M, Mentges D, Papsdorf M, Hempelmann G. Volume therapy in the critically ill: is there a difference? Intensive Care Med. 1998 Jan;24(1):28-36. Retraction in: Intensive Care Med. 2014 Jan;40(1):145.
  - Haisch G, Boldt J, Krebs C, Kumle B, Suttner S, Schulz A. The influence of intravascular volume therapy with a new hydroxyethyl starch preparation (6% HES 130/0.4) on coagulation in patients undergoing major abdominal surgery. Anesth Analg. 2001 Mar;92(3):565-71. Retraction in: Anesth Analg. 2011 May;112(5):1225.
  - Lang K, Boldt J, Suttner S, Haisch G. Colloids versus crystalloids and tissue oxygen tension in patients undergoing major abdominal surgery. Anesth Analg. 2001 Aug;93(2):405-9 , 3rd contents page. Retraction in: Anesth Analg. 2011 May;112(5):1211.
  - Boldt J, Haisch G, Suttner S, Kumle B, Schellhase F. Are lactated Ringer's solution and normal saline solution equal with regard to coagulation? Anesth Analg. 2002 Feb;94(2):378-84, table of contents. Retraction in: Anesth Analg. 2011 May;112(5):1178.
  - Boldt J, Suttner S, Brosch C, Lehmann A, Röhm K, Mengistu A. The influence of a balanced volume replacement concept on inflammation, endothelial activation, and kidney integrity in elderly cardiac surgery patients. Intensive Care Med. 2009 Mar;35(3):462-70. doi: 10.1007/s00134-008-1287-1. Epub 2008 Sep 20. Retraction in: Intensive Care Med. 2011 Jul;37(7):1233.
- **Retrospective or cross over studies, not randomized controlled trials (only list large trials or included in previous meta-analysis inadequately)**
  - Raghunathan K, Bonavia A, Nathanson BH, Beadles CA, Shaw AD, Brookhart MA, Miller TE, Lindenauer PK. Association between Initial Fluid Choice and Subsequent In-hospital Mortality during the Resuscitation of Adults with Septic Shock. Anesthesiology. 2015 Dec;123(6):1385-93. doi: 10.1097/ALN.0000000000000861.
  - Raghunathan K, Shaw A, Nathanson B, Stürmer T, Brookhart A, Stefan MS, Setoguchi S, Beadles C, Lindenauer PK. Association between the choice of IV crystalloid and in-hospital mortality among critically ill adults with sepsis. Crit Care Med. 2014 Jul;42(7):1585-91. doi: 10.1097/CCM.0000000000000305.
  - Leberle R, Ernstberger A, Loibl M, Merkl J, Bunz M, Creutzenberg M, Trabold B. Association of high volumes of hydroxyethyl starch with acute kidney injury in elderly trauma patients. Injury. 2015 Jan;46(1):105-9. doi: 10.1016/j.injury.2014.08.039.
  - Sen A, Keener CM, Sileanu FE, Foldes E, Clermont G, Murugan R, Kellum JA. Chloride Content of Fluids Used for Large-Volume Resuscitation Is Associated With Reduced Survival. Crit Care Med. 2017 Feb;45(2):e146-e153. doi: 10.1097/CCM.0000000000002063.
  - Yunos NM, Bellomo R, Hegarty C, Story D, Ho L, Bailey M. Association between a chloride-liberal vs chloride-restrictive intravenous fluid administration strategy and kidney injury in critically ill adults. JAMA. 2012 Oct 17;308(15):1566-72. doi: 10.1001/jama.2012.13356.
  - Zhu GC, Quan ZY, Shao YS, Zhao JG, Zhang YT. The study of hypertonic saline and hydroxyethyl starch treating severe sepsis. Zhongguo Wei Zhong Bing Ji Jiu Yi Xue. 2011 Mar;23(3):150-3.
  - Parker MJ, Griffiths R, Boyle A. Preoperative saline versus gelatin for hip fracture patients; a randomized trial of 396 patients. Br J Anaesth. 2004 Jan;92(1):67-70.
  - Shaw AD, Bagshaw SM, Goldstein SL, Scherer LA, Duan M, Schermer CR, Kellum JA. Major complications, mortality, and resource utilization after open abdominal surgery: 0.9% saline compared to Plasma-Lyte. Ann Surg. 2012 May;255(5):821-9.
- **Mixed all population in one studies, no matching nor subgroup data can be extracted (list trials included in previous meta-analysis inadequately)**
  - Haupt MT, Rackow EC. Colloid osmotic pressure and fluid resuscitation with hetastarch, albumin, and saline solutions. Crit Care Med. 1982 Mar;10(3):159-62.
  - Metildi LA, Shackford SR, Virgilio RW, Peters RM.Crystalloid versus colloid in fluid resuscitation of patients with severe pulmonary insufficiency. Surg Gynecol Obstet. 1984 Mar;158(3):207-12.
  - van der Heijden M, Verheij J, van Nieuw Amerongen GP, Groeneveld AB. Crystalloid or colloid fluid loading and pulmonary permeability, edema, and injury in septic and nonseptic critically ill patients with hypovolemia. Crit Care Med. 2009 Apr;37(4):1275-81. doi: 10.1097/CCM.0b013e31819cedfd.
  - Wu JJ1, Huang MS, Tang GJ, Kao WF, Shih HC, Su CH, Lee CH. Hemodynamic response of modified fluid gelatin compared with lactated ringer's solution for volume expansion in emergency resuscitation of hypovolemic shock patients: preliminary report of a prospective, randomized trial. World J Surg. 2001 May;25(5):598-602.
  - Stockwell MA, Soni N, Riley B. Colloid solutions in the critically ill. A randomised comparison of albumin and polygeline. 1. Outcome and duration of stay in the intensive care unit. Anaesthesia. 1992 Jan;47(1):3-6.
  - Beards SC, Watt T, Edwards JD, Nightingale P, Farragher EB. Comparison of the hemodynamic and oxygen transport responses to modified fluid gelatin and hetastarch in critically ill patients: a prospective, randomized trial. Crit Care Med. 1994 Apr;22(4):600-5.
- **Pilot study for another randomized controlled trail (also use for sensitivity analysis)**
  - Semler MW, Wanderer JP, Ehrenfeld JM, Stollings JL, Self WH, Siew ED, Wang L, Byrne DW, Shaw AD, Bernard GR, Rice TW; SALT Investigators and the Pragmatic Critical Care Research Group; SALT Investigators. Balanced Crystalloids versus Saline in the Intensive Care Unit. The SALT Randomized Trial. Am J Respir Crit Care Med. 2017 May 15;195(10):1362-1372. doi: 10.1164/rccm.201607-1345OC.
- **Study on children or less than 18 years of age**
  - Maitland K, Pamba A, English M, Peshu N, Marsh K, Newton C, Levin M. Randomized trial of volume expansion with albumin or saline in children with severe malaria: preliminary evidence of albumin benefit. Clin Infect Dis. 2005 Feb 15;40(4):538-45. Epub 2005 Jan 25.
  - Maitland K, Kiguli S, Opoka RO, Engoru C, Olupot-Olupot P, Akech SO, Nyeko R, Mtove G, Reyburn H, Lang T, Brent B, Evans JA, Tibenderana JK, Crawley J, Russell EC, Levin M, Babiker AG, Gibb DM; FEAST Trial Group. Mortality after fluid bolus in African children with severe infection. N Engl J Med. 2011 Jun 30;364(26):2483-95. doi: 10.1056/NEJMoa1101549. Epub 2011 May 26.
  - Van der Linden P, Dumoulin M, Van Lerberghe C, Torres CS, Willems A, Faraoni D. Efficacy and safety of 6% hydroxyethyl starch 130/0.4 (Voluven) for perioperative volume replacement in children undergoing cardiac surgery: a propensity-matched analysis.
- **Priming solution for cardiopulmonary bypass surgery**
  - Himpe D, Van Cauwelaert P, Neels H, Stinkens D, Van den Fonteyne F, Theunissen W, Muylaert P, Hermans C, Goossens G, Moeskops J, et al. Priming solutions for cardiopulmonary bypass: comparison of three colloids. J Cardiothorac Vasc Anesth. 1991 Oct;5(5):457-66.
  - Tigchelaar I, Gallandat Huet RC, Boonstra PW, van Oeveren W. Comparison of three plasma expanders used as priming fluids in cardiopulmonary bypass patients. Perfusion. 1998 Sep;13(5):297-303.
- **Fixed volume for fluid resuscitation**
  - Verheij J1, van Lingen A, Beishuizen A, Christiaans HM, de Jong JR, Girbes AR, Wisselink W, Rauwerda JA, Huybregts MA, Groeneveld AB. Cardiac response is greater for colloid than saline fluid loading after cardiac or vascular surgery. Intensive Care Med. 2006 Jul;32(7):1030-8.
- **Only hemodynamic results, no mortality data available**
  - Trof RJ1, Sukul SP, Twisk JW, Girbes AR, Groeneveld AB. Greater cardiac response of colloid than saline fluid loading in septic and non-septic critically ill patients with clinical hypovolaemia. Intensive Care Med. 2010 Apr;36(4):697-701.
  - Pfortmueller CA, Funk GC, Reiterer C, Schrott A, Zotti O, Kabon B, Fleischmann E, Lindner G.Saline versus a balanced crystalloid for goal-directed perioperative fluid therapy in major abdominal surgery: a double-blind randomised controlled study.
- **Mixed all population in one study (matched well in specific population or clear subgroup data, extract specific subpopulation data into current NMA)**
  - Finfer S, Bellomo R, Boyce N, French J, Myburgh J, Norton R; SAFE Study Investigators. A comparison of albumin and saline for fluid resuscitation in the intensive care unit. N Engl J Med. 2004 May 27;350(22):2247-56.
  - Myburgh JA, Finfer S, Bellomo R, Billot L, Cass A, Gattas D, Glass P, Lipman J, Liu B, McArthur C, McGuinness S, Rajbhandari D, Taylor CB, Webb SA; CHEST Investigators; Australian and New Zealand Intensive Care Society Clinical Trials Group. Hydroxyethyl starch or saline for fluid resuscitation in intensive care. N Engl J Med. 2012 Nov 15;367(20):1901-11. doi: 10.1056/NEJMoa1209759. Epub 2012 Oct 17.
  - Annane D, Siami S, Jaber S, Martin C, Elatrous S, Declère AD, Preiser JC, Outin H, Troché G, Charpentier C, Trouillet JL, Kimmoun A, Forceville X, Darmon M, Lesur O, Reignier J, Abroug F, Berger P, Clec'h C, Cousson J, Thibault L, Chevret S; CRISTAL Investigators. Effects of fluid resuscitation with colloids vs crystalloids on mortality in critically ill patients presenting with hypovolemic shock: the CRISTAL randomized trial. JAMA. 2013 Nov 6;310(17):1809-17. doi: 10.1001/jama.2013.280502.
  - Young P, Bailey M, Beasley R, Henderson S, Mackle D, McArthur C, McGuinness S, Mehrtens J, Myburgh J, Psirides A, Effect of a Buffered Crystalloid Solution vs Saline on Acute Kidney Injury Among Patients in the Intensive Care Unit: The SPLIT Randomized Clinical Trial. Reddy S3, Bellomo R10; SPLIT Investigators; ANZICS CTG. JAMA. 2015 Oct 27;314(16):1701-10. doi: 10.1001/jama.2015.12334.
  - Semler MW, Self WH, Wanderer JP, Ehrenfeld JM, Wang L, Byrne DW, Stollings JL, Kumar AB, Hughes CG, Hernandez A, Guillamondegui OD, May AK, Weavind L, Casey JD, Siew ED, Shaw AD, Bernard GR, Rice TW; SMART Investigators and the Pragmatic Critical Care Research Group. Balanced Crystalloids versus Saline in Critically Ill Adults. N Engl J Med. 2018 Mar 1;378(9):829-839. doi: 10.1056/NEJMoa1711584

# Appendix 5: List of included studies

## 5.1. Sepsis patients

### 5.1.1. Study group and primary outcome

eTable 5.1 Randomized controlled trials in sepsis patients

| **Author, Year (Study group)** | **Country** | **Centers** | **No. of sepsis cases** | **Primary outcome** | **Mortality** |
| --- | --- | --- | --- | --- | --- |
| Rackow et al, 1983 | USA | 1 | 26 | Hemodynamic parameters | In-hospital mortality |
| Rackow et al, 1989 | USA | 1 | 20 | Hemodynamic parameters | In-hospital mortality |
| Asfar et al, 2000 | France | 1 | 34 | Hemodynamic parameters | In-hospital mortality |
| Schortgen et al, 2001 | France | 3 | 129 | Acute renal failure | In-hospital mortality |
| Finfer et al, 2004 (SAFE) | Aus. & NZ | 16 | 1218 | Mortality | 28-day mortality |
| Molnar et al, 2004 | UK | 1 | 30 | Hemodynamic parameters | In-hospital mortality |
| Veneman et al, 2004 | Netherlands | 1 | 61 | Plasma colloid pressure. | 30-day mortality |
| Palumbo et al, 2006 | Italy | 1 | 20 | Hemodynamic parameters. | 5-day mortality |
| Brunkhorst et al, 2008 (VISEP) | Germany | 18 | 537 | Mortality | 90-day mortality |
| Friedman et al, 2008 | Belgium | 1 | 34 | Hemodynamic parameters. | In-hospital mortality |
| Li et al, 2008 | China | 1 | 60 | Mortality | 28-day mortality |
| McIntyre et al, 2008 (FINESS) | Canada and NZ | 4 | 40 | Feasibility trial. | 28-day mortality |
| Dolecek et al, 2009 | Czech Republic | 1 | 56 | Hemodynamic parameters. | In-hospital mortality |
| Dubin et al, 2010 | Argentina | 2 | 20 | Sublingual microcirculation. | 31-day mortality |
| Charpentier et al, 2011 (EARSS) | France | 29 | 798 | Mortality. | 28-day mortality. |
| Siegemund, 2011 (BaSES) | Netherlands | 1 | 240 | Hemodynamic parameters. | In-hospital mortality |
| Guidet et al, 2012 (CRYSTMAS) | France and Germany | 24 | 196 | Volume for hemodynamic stable. | 90-days mortality |
| Myburgh et al, 2012 (CHEST) | Australia and NZ | 32 | 1937 | Mortality | 90-days mortality |
| Perner et al, 2012 (6S) | Scandanavia | 26 | 798 | Mortality | 90-days mortality |
| Annane et al, 2013 (CRISTAL) | Worldwide | 57 | 1553 | Mortality | 90-days mortality |
| Caironi et al, 2014 (ALBIOS) | Italy | 100 | 1818 | Mortality | 90-days mortality |
| Young et al, 2015 (SPLIT) | Aus. & NZ | 4 | 77 | Acute renal failure. | In-hospital mortality |
| Matthew W. et al, 2018 (SMART) | USA | 1 | 2336 | Mortality and acute kidney injury | 30-days mortality |

### 5.1.2. Study population and enrollment criteria

eTable 5.2 Sepsis definition and inclusion criteria in trials with sepsis patients

| **Author, Year** | **Study population** | **Sepsis definition** | **Inclusion** | **Exclusion** |
| --- | --- | --- | --- | --- |
| Rackow et al, 1983 | 26 hypovolemic shock patients. | Infection and meet the following inclusion criteria | Systemic hypoperfusion : (1) SBP<90mmHg or (2) cardiac index<2.2 l/min*m^2 or (3) lactate>18mg/dl and (4) WP<15mmHG | <18 yr of age, terminal state, significant coagulopathy |
| Rackow et al, 1989 | 20 severe sepsis patients. | Infection and three of following criteria: (1) BT>38 or <36 degree, (2) HR>100/min, (3) RR>20/min, (4) WBC>15000/cumm or <3500/cumm, (5) platelet<150000/cumm | Systemic hypoperfusion : (1) SBP<90mmHg or (2) cardiac index<2.2 l/min*m^2 or (3) lactate>18mg/dl and (4) WP<12mmHG | <21 yr of age, terminal state, spontaneous bleeding |
| Asfar et al, 2000 | 34 septic hypovolemic ventilated and hemodynamically controlled patients. | Infection and three of following criteria: (1) BT>38.2 or <35.5degree, (2) HR>100/min, (3) controlled ventilatoion, (4) WBC>12,000/cumm or <3,500/cumm, (5) platelet<100,000/cumm | SBP<90mmHg or PAWP<12mmHg and gastric pH<7.35 or PCO2 gastric-artery gradient > 8mmHg | <16 yr of age, overt hemodynamic, ventilatory or acid base status instability |
| Schortgen et al, 2001 | 129 severe sepsis or septic shock ICU patients who requred fluid loading | Infection and any of following criteria: (1) BT>38 or <36 degree, (2) HR>90/min, (3) RR>20/min or controlled ventilatoion, (4) WBC>12,000/cumm or <4,000/cumm | At least one of the following six criteria: SBP<90mmHg, metabolic acidosis, hypoxemia, oliguria, coagulopathy or encephalopathy | <18 yr of age or pregnancy, history of allergy to starch or gelatin, severe renal dysfunction (creatine concentration >320 mmol/l or a need of dialysis) |

eTable 5.2 Sepsis definition and inclusion criteria in trials with sepsis patients (continued)

| **Author, Year** | **Study population** | **Sepsis def.** | **Inclusion** | **Exclusion** |
| --- | --- | --- | --- | --- |
| Finfer et al, 2004 (SAFE) | 6997 ICU patients requiring fluid administration. 1218 were severe sepsis patients. | Sepsis 2** | At least one of the following clinical signs : (1) HR>90/min, (2) SBP<100mmHg or MAP<75mmHg, (3) CVP<10mmHg, (4) PAWP<12mmHg, (5) Respiratory variation in SBP>5mmHg, (6) capillary refill time>1 sec., (7) urine output<0.5ml/kg for one hour. | <18 yr of age, admission to the ICU following cardiac surgery, body burn, liver transplantation surgery; previously received fluid resuscitation during the current admission |
| Molnar et al, 2004 | 30 septic shock with acute lung injury and hypovelemic shock patients. | Sepsis 2** | Septic shock and severe respiratory failure | Chronic cardiovascular failure (NYHA class IV), chronic respiratory failure, chronic renal failure requiring renal replacement therapy, choic liver failure or those with diabetes mellitus or with known aortic aneurysm. |
| Veneman et al, 2004 | 61 severely ill patient. | NI* | Hypoalbuminemic (serum albumin concentration <20g/l). | Two patients died shortly after randomization were excluded. |
| Palumbo et al, 2006 | 20 patients with severe sepsis. | NI* | NI | <21 yr of age, renal dysfunction, severe liver failure, DIC and considered to be in a terminal state |
| Brunkhorst et al, 2008 (VISEP) | 537 severe sepsis or septic shock patients | Sepsis 2** | Onset of the syndrome was less than 24 hours before ICU admission or less than 12 hours after admission. | Received HES more than 1000m in the 24 hours before randomisation. |
| Friedman et al, 2008 | 34 sepsis and suspected hypovolemia patients. | Sepsis 2** | suspected hypovolemia but relative hemodynamic stability. | A patient was excluded if hemodynamic instability required aggressive resuscitation during the first 100 minutes. |
| Li et al, 2008 | 60 septic shock patients. | Sepsis 2** | MAP <60mmHg or SBP dropped more than 40 mmHg. | Coagulopathy, renal replacement therapy, pregnant, congestive heart failure, history of cardiac arrest and expired less than 72 hours after admission. |

*NI, no information；** 2001 SCCM/ESICM/ACCP/ATS/SIS International Sepsis Definitions Conference.

eTable 5.2 Sepsis definition and inclusion criteria in trials with sepsis patients (continued)

| **Author, Year** | **Study population** | **Sepsis def.** | **Inclusion** | **Exclusion** |
| --- | --- | --- | --- | --- |
| McIntyre et al, 2008 (FINESS) | 40 early septic shock patients. | Sepsis 2* | persistent hypotention after more than 1 liter of crystalloid fluid were recruited. | <18 yr of age, other forms of shock, chronic renal failure requiring dialysis, pregnancy, previous admission to ICU with septic shock during the present hospitalization. |
| Dolecek et al, 2009 | 56 severe sepsis patients. | Sepsis 2* | NI | NI |
| Dubin et al, 2010 | 20 severe sepsis patients. | Sepsis 2* | tissue hypoperfusion as MAP< 65mmHg despite a crystalloid fluid challenge of 20 ml/kg or blood lactate >4mmol/l. | impossibility to perform sublingual bideomicroscopy, <18yr, pregnancy, stroke, AMI, hydrostatic pulmonary edema, arrythmia. |
| Charpentier et al, 2011 (EARSS) | 798 septic shock patient. | Sepsis 2* | within 6 hours after catecholamine introduction. | overweight severe heart failure, neutropenia, cirrhosis and severe burn. |
| Siegemund, 2011 (BaSES) | 240 sepsis patients. | Sepsis 2* | NI | NI |
| Guidet et al, 2012 (CRYSTMAS) | 196 severe sepsis patients. | Sepsis 2* | NI | <18 yr of age, known serum creatine>3.39mg/dl, anuria lating more than 8 hours despite fluid resuscitation, requirment for renal support |
| Myburgh et al, 2012 (CHEST) | 7000 patient require fluid resuscitation. | Sepsis 2* | clinician judge to require fluid resuscitation. | <18 yr of age, current dialysis-dependent renal failure, evidence of intracranial hemorrhage on CT scan. |
| Perner et al, 2012 (6S) | 804 severe sepsis patients | Sepsis 2* | Sepsis plus at least one SOFA score > 2 for the organ in question | <18 yr of age, underwent renal replacement therapy, burn injury more than 10%, intracranial bleeding, potassium more than 6mmol/l |

* 2001 SCCM/ESICM/ACCP/ATS/SIS International Sepsis Definitions Conference.

eTable 5.2 Sepsis definition and inclusion criteria in trials with sepsis patients (continued)

| **Author, Year** | **Study population** | **Sepsis def.** | **Inclusion** | **Exclusion** |
| --- | --- | --- | --- | --- |
| Annane et al, 2013 (CRISTAL) | 2857 hypovolemic shock patients | Sepsis 2** | Sepsis, multiple trauma or other cause of hypovolemic shock. | anesthesia-related hypotension, advanced chronic liver disease, chronic renal failure, acute anaphylactitc reaction, inherited coagulation disorders, do-not-resuscitate order, pregnant, burn injury more than 20% body surface area |
| Caironi et al, 2014 (ALBIOS) | 1818 severe sepsis or septic shock. | Sepsis 2** | severe sepsis or septic shock. | <18 yr of age, terminal state, congestive heart failure, suspected head injury, pathological conditions in which albumin administration is clinically indicated (cirrhosis, nephrotic syndrome, burn) |
| Young et al, 2015 (SPLIT) | 2278 ICU patient required fluid resuscitation. | NI* | ICU patient required fluid resuscitation. | require renal replacement within 6 hours after enrollment, usually on dialysis for end-stage renal failure, palliative care. |
| Matthew W. et al, 2018 | 15802 critically ill adults required fluid administration. | ICD 10 | critically ill adults required fluid administration. | <18 yr of age. |

*NI, no information；** 2001 SCCM/ESICM/ACCP/ATS/SIS International Sepsis Definitions Conference.

### 5.1.3. Baseline characteristics

eTable 5.3 Baseline characteristics in trials with sepsis patients

| **Author, Year** | **Arm** | **Age,**  **yr (mean)** | **Male,**  **(%)** | **Lung inf. (%)** | **Severity of illness, mean±SD** | **MAP,**  **mmHg** | **Vasopressor,**  **(%)** | **Lactate,**  **mg/dl** |
| --- | --- | --- | --- | --- | --- | --- | --- | --- |
| Rackow et al, 1983 | Saline | 82 | 68 |  |  | 54.7 |  | 42 |
|  | Iso-oncotic albumin | 80 | 78 |  |  | 60.3 |  | 53.9 |
|  | L-HES | 72 | 50 |  |  | 63.7 |  | 64.4 |
| Rackow et al, 1989 | Iso-oncotic albumin | 65.1 | 80 | 25 |  | 67.1 |  | 50.4 |
|  | H-HES | 73.5 | 70 | 30 |  | 57.2 |  | 54 |
| Asfar et al, 2000 | H-HES | 64 | 75 | 56 | SAPS II=52±26 | 75 | 50 |  |
|  | Gelatin | 65 | 78 | 56 | SAPS II=46±19 | 76 | 33 |  |
| Schortgen et al, 2001 | H-HES | 60 | 66 | 44 | SAPS II=53±7.4 | 80 | 49 |  |
|  | Gelatin | 56 | 71 | 52 | SAPS II=50±5.6 | 82 | 41 |  |
| Finfer et al, 2004  (SAFE) | Saline | 58.5 | 61 |  | APACHE=19±8.0 | 78.2 |  |  |
|  | Iso-oncotic albumin | 58.6 | 59 |  | APACHE=18.7±7.9 | 77.8 |  |  |
| Molnar et al, 2004 | H-HES | 49 | 36 | 0 | SAPS2=34±21 | 76 |  |  |
|  | gelatin | 56 | 36 | 6 | SAPS2=34±14 | 73 |  |  |
| Veneman et al, 2004 | Saline | 67 | 63 |  | APACHE-II=19±4 | 79 | 56 | 11.3 |
|  | Hyperoncotic albumin | 72 | 53 |  | APACHE-II=22±8 | 85 | 60 | 11.7 |
|  | L-HES | 69.5 | 60 |  | APACHE-II=23.5±6.5 | 81.5 | 56.5 | 13.1 |
| Palumbo et al, 2006 | Hyperoncotic albumin | 59.6 | 50 |  | APACHE-II=19.7±3.6 | 78 |  |  |
|  | L-HES | 59.6 | 50 |  | APACHE=18.5±3.6 | 75 |  |  |
| Brunkhorst et al, 2008  (VISEP) | Ringer's lactate | 64.9 | 60 | 45 | APACHE=20.3±6.7 | 75 |  | 20.8 |
|  | H-HES | 64.4 | 60 | 37 | APACHE=20.1±6.7 | 75.5 |  | 20.8 |
| Friedman et al, 2008 | Iso-oncotic albumin | 66 | 62 |  |  | 79 | 84.6 |  |
|  | H-HES | 64 | 81 |  |  | 71.5 | 76.1 |  |
| Li et al, 2008 | Saline | 38.6 | 73 |  | APACHE=18.7±10.3 | 80.9 | 93.3 | 53.9 |
|  | H-HES | 44.8 | 67 |  | APACHE=15.2±7.0 | 78.3 | 93.3 | 58.9 |
| McIntyre et al, 2008  (FINESS) | Saline | 63.6 | 58 |  | APACHE=20.2±6.3 | 60.9 | 32 |  |
|  | H-HES | 63.1 | 62 |  | APACHE=21.1±6.1 | 59.7 | 57 |  |
| Dolecek et al, 2009 | Hyperoncotic albumin | 47 |  |  | SOFA=8±3 |  |  |  |
|  | L-HES | 43 |  |  | SOFA=8±3 |  |  |  |
| Dubin et al, 2010 | Saline | 65 | 55 |  | SOFA=8.9±3.6 | 55 |  | 34.2 |
|  | L-HES | 62 | 67 |  | SOFA=8.1±2.5 | 56 |  | 27 |
| Charpentier et al, 2011  (EARSS) | Saline | 66 | 67 | 45 | SOFA=10±3.1 |  |  | 2.2 |
|  | Hyperoncotic albumin | 66 | 67 | 45 | SOFA=10±3.1 |  |  | 2.2 |
| Siegemund, 2011 | Saline, L-HES | NI |  |  |  |  |  |  |
| Guidet et al, 2012  (CRYSTMAS) | Saline | 65.9 | 64 | 53 | SOFA=7.9, SAPS II=53±7.4 |  | 88 |  |
|  | L-HES | 65.8 | 59 | 60 | SOFA=9.1, SAPS II=50 |  | 87 |  |
| Myburgh et al, 2012  (CHEST) | Saline | 62.9 | 60 |  | APACHE=17.0±13.11 | 73.7 | 46.1 | 18 |
|  | L-HES | 63.1 | 61 |  | APACHE=17.0±8.14 | 74 | 45.5 | 18.9 |
| Perner et al, 2012  (6S) | Ringer's lactate | 67 | 61 | 53 | SOFA=4.0±2.96, SAPS II=51±17.03 |  | 84 | 18.9 |
|  | L-HES | 66 | 60 | 57 | SOFA=4.0±2.96, SAPS II=50±14.81 |  | 84 | 18 |
| Annane et al, 2013  (CRISTAL) | Crystalloids | 63 | 63 |  | SOFA=8±4.3, SAPS II=50±21 | 66 |  | 21.6 |
|  | Colloids | 63 | 63 |  | SOFA=8±4.3, SAPS II=48±21 | 64.6 |  | 20.7 |
| Caironi et al, 2014  (ALBIOS) | Saline | 69 | 61 | 42 | SOFA=8±3.7, SAPS II=48±17 | 73 |  | 22.5 |
|  | Hyperoncotic albumin | 70 | 60 | 39 | SOFA=8±3.0, SAPS II=48±14.8 | 74 |  | 20.7 |
| Young et al, 2015  (SPLIT) | Balanced crystalloids | 60.1 | 64 |  | APACHE=14.1±6.9 |  |  |  |
|  | Saline | 61 | 67 |  | APACHE=14.1±6.7 |  |  |  |
| Matthew W. et al, 2018 | Balanced crystalloids | 58 | 57 |  |  |  | 26.4 |  |
|  | Saline | 58 | 58 |  |  |  | 26.2 |  |

### 5.1.4. Resuscitation goal and fluid volume

eTable 5.4 Resuscitation goal and fluid balance in trials with sepsis patients

| **Author, Year** | **Resuscitation target** | **Arm** | **Study fluid volume** | | **Total input** | | **Urine volume** | | **Cumulative net** | | **Duration**  **(hour)** |
| --- | --- | --- | --- | --- | --- | --- | --- | --- | --- | --- | --- |
|  |  |  | **Mean** | **SD** | **Mean** | **SD** | **Mean** | **SD** | **Mean** | **SD** |  |
| Rackow et al, 1983 | 250ml per 15 min to achieved wedge pressure equalled to 15mmHg | Saline | 8356 | 1874 | 10287 | 2074 | 1721 | 327.4 | 8566 | 2227 | 24 |
|  |  | Iso-oncotic albumin | 2833 | 266.8 | 4703 | 452 | 2202 | 351.9 | 2500 | 568 | 24 |
|  |  | L-HES | 4569 | 1202 | 6923 | 1190 | 1734 | 400.1 | 5189 | 1300 | 24 |
| Rackow et al, 1989 | 250ml per 15 min to achieved wedge pressure equalled to 15mmHg | Iso-oncotic albumin | 975 | 169 | 975 | 169 |  |  |  |  | 0.8 |
|  |  | H-HES | 900 | 205 | 900 | 205 |  |  |  |  | 0.8 |
| Asfar et al, 2000 | only 500ml study fluid infused in 30minutes, no target | H-HES | 500 | 0 | 500 | 0 | 48 | 22 |  |  | 0.5 |
|  |  | gelatin | 500 | 0 | 500 | 0 | 95 | 101 |  |  | 0.5 |
| Schortgen et al, 2001 | Reversal of shock or organ dysfunction. | H-HES | 31ml/kg | |  |  |  |  |  |  | 96 |
|  |  | gelatin | 43ml/kg | |  |  |  |  |  |  | 96 |
| Finfer et al, 2004 (SAFE) | The treating clinicians determined | Saline | 3094 |  | 9582 |  |  |  | 4204 |  | 96 |
|  |  | Iso-oncotic albumin | 2247 |  | 9385 |  |  |  | 3118 |  | 96 |
| Molnar et al, 2004 | ITBVI>900 ml/m2 | H-HES | 1000 | 0 |  |  | 837 |  |  |  | 24 |
|  |  | gelatin | 1000 | 0 |  |  | 204 |  |  |  | 24 |
| Veneman et al, 2004 | MAP=70mmHG, CVP=5-10 mmHG | Saline | 3000 | 0 |  |  |  |  | 2488 |  | 72 |
|  |  | Hyperoncotic albumin | 900 | 0 |  |  |  |  | 2219 |  | 72 |
|  |  | L-HES | 2250 | 0 |  |  |  |  | 2190 |  | 72 |
| Palumbo et al, 2006 | PCWP=15-18mmHg | Hyperoncotic albumin |  |  |  |  |  |  |  |  | 120 |
|  |  | L-HES |  |  |  |  |  |  |  |  | 120 |

| **Author, Year** | **Resuscitation target** | **Arm** | **Study fluid volume** | | **Total input** | | **Urine volume** | | **Cumulative net** | | **Duration**  **(hour)** |
| --- | --- | --- | --- | --- | --- | --- | --- | --- | --- | --- | --- |
|  |  |  | **Mean** | **SD** | **Mean** | **SD** | **Mean** | **SD** | **Mean** | **SD** |  |
| Brunkhorst et al, 2008 (VISEP) | CVP<8mmHg, MAP<70mmHg | Ringer's lactate | 92.9ml/kg | |  |  |  |  |  |  | 504 |
|  |  | H-HES | 70.4ml/kg | |  |  |  |  |  |  | 504 |
| Friedman et al, 2008 | NI | Iso-oncotic albumin | 400 |  |  |  |  |  | 280 |  | 40 |
|  |  | H-HES | 400 |  |  |  |  |  | 88.5 |  | 40 |
| Li et al, 2008 | CVP>12mmHg, MAP>65mmHg, Urine output>0.5ml/kg/hr, ScvO2>70mmHg | Saline | 466.7 | 129.1 | 2917 | 579.9 |  |  | 921 | 1069 | 24 |
|  |  | H-HES | 100 | 338.1 | 2800 | 1014 |  |  | 982 | 1161 | 24 |
| McIntyre et al, 2008 (FINESS) | CVP=8-12mmHg, ScvO2>70% | Saline | 2100 | 600 |  |  |  |  |  |  | 24 |
|  |  | H-HES | 1900 | 600 |  |  |  |  |  |  | 24 |
| Dolecek et al, 2009 | ITBVI<850ml/m2, or CI<3.5l/min/m2 | Hyperoncotic albumin | 100ml q12h | |  |  |  |  |  |  | 72 |
|  |  | L-HES | 250ml q6h | |  |  |  |  |  |  | 72 |
| Dubin et al, 2010 | CVP=8-12mmHg, MAP=65, ScvO2=70% | Saline | 6254 |  | 8368 | 2405 | 1507 | 1350 | 6606 | 2669 | 24 |
|  |  | L-HES | 2610 |  | 4682 | 1371 | 1825 | 863 | 2857 | 1596 | 24 |
| Charpentier et al, 2011 (EARSS) | NI | Saline | NI |  |  |  |  |  |  |  | 72 |
|  |  | Hyperoncotic albumin | NI |  |  |  |  |  |  |  | 72 |
| Siegemund, 2011 (BaSES) | NI | Saline | NI |  |  |  |  |  |  |  | 124 |
|  |  | L-HES | 3775 |  |  |  |  |  |  |  | 124 |

| **Author, Year** | **Resuscitation target** | **Arm** | **Study fluid volume** | | **Total input** | | **Urine volume** | | **Duration**  **(hour)** |
| --- | --- | --- | --- | --- | --- | --- | --- | --- | --- |
|  |  |  | **Mean** | **SD** | **Mean** | **SD** | **Mean** | **SD** |  |
| Guidet et al, 2012 (CRYSTMAS) | Hemodynamic stable (MAP>65mmHG, CVP=8-12mmHG, ScVO2>70%, urine output<2ml/kg) | Saline | 1379 | 886 |  |  |  |  | 96 |
|  |  | L-HES | 1709 | 1164 |  |  |  |  | 96 |
| Myburgh et al, 2012 (CHEST) | stopped in patients who were treated with renal replacement therapy. | Saline | 616 | 488 | 1377 | 550 |  |  | 96 |
|  |  | L-HES | 526 | 425 | 1731 | 740 |  |  | 96 |
| Perner et al, 2012 (6S) | The treating clinicians determined | Ringer's lactate | 3500 | 370.3 |  |  | 13700 | 3312.33 | 72 |
|  |  | L-HES | 4000 | 308.5 |  |  | 14890 | 3350.5 | 72 |
| Annane et al, 2013 (CRISTAL) | The treating clinicians determined | Ringer's lactate | 3000 | 740.7 |  |  |  |  | 168 |
|  |  | Saline | 1000 | 246.8 |  |  |  |  | 168 |
|  |  | Hyperoncotic albumin | 300 | 49.33 |  |  |  |  | 168 |
|  |  | L-HES | 1500 | 123.3 |  |  |  |  | 168 |
|  |  | gelatin | 1500 | 246.8 |  |  |  |  | 168 |
| Caironi et al, 2014 (ALBIOS) | EGDT goal | Saline | 1100 | 185.2 | 31970 | 7313 | 22261 | 6189.75 | 168 |
|  |  | Hyperoncotic albumin | 0 | 0 | 31867 | 7884 | 23282 | 6434.1 | 169 |
| Young et al, 2015 (SPLIT) | The treating clinicians determined | Balanced crystalloids | 2655 | 3052 |  |  |  |  | 96 |
|  |  | Saline | 2554 | 2120 |  |  |  |  | 96 |
| Matthew et al, 2018 | The treating clinicians determined | Balanced crystalloids | 2117 | 3157 |  |  |  |  | 168 |
|  |  | Saline | 2775 | 3488 |  |  |  |  | 168 |

## 5.2. Surgical patients

### 5.2.1 Population, intervention, outcome

eTable 5.5. Study characteristics in surgical patients

| Author, Year | study population | interventions compared | Colloid fluid detail | Primary outcome |
| --- | --- | --- | --- | --- |
| Boutros et al, 1979 | abdominal aortic surgery | Ringer's lactate, 0.45% saline, albumin |  | hemodynamic parameters |
| Virgilio et al, 1979 | abdominal aortic surgery | Ringer's lactate, albumin | 5% albumin | hemodynamic parameters |
| Zetterstrom et al, 1981 | abdominal aortic surgery | Ringer's lactate, albumin | 5% albumin | hemodynamic parameters |
| Shires et al, 1983 | aortic surgery | Ringer's lactate, albumin |  | hemodynamic parameters |
| Prien et al, 1990 | Gastrointestinal surgery (hemipancreatoduodenectomy) | Ringer's lactate, albumin, H-HES | 20% albumin, Rhodalbumin, France; 10% HES-steril, Fresenius AG, Germany | hemodynamic parameters |
| Wahba et al, 1996 | coronary artery bypass surgery | Ringer's lactate, Gelatin | Haemaccel(degraded delatin) | hemodynamic parameters |
| Waters et al, 2001 | abdominal aortic surgery | Ringer's lactate, Saline |  | hemodynamic parameters |
| Linden et al, 2003 | cardiac surgery | H-HES, gelatin | 6% 200, Haesteril, Fresenius Kabi, Germany; Haemaccel, Hoechst, Germany | hemodynamic parameters |
| Linden et al, 2005 | cardiac surgery | L-HES, gelatin | 6% 130, Voluven, Fresenius Kabi, Germany; 3% Geloplasma, Fresenius Kabi | hemodynamic parameters |

eTable 5.5. Study characteristics in surgical patients (continued)

| Mahmood et al. 2007 | aortic aneurysm surgery | L-HES, H-HES, Gelatin | 6% 130/0.4, Voluven, Fresenius Kabi; 6% 200/0.62, Elohes, Fresenius Kabi, UK; 4% gelofusine, Braun, UK | renal outcome |
| --- | --- | --- | --- | --- |
| Tamayo et al, 2008 | cardiac surgery | Ringer's lactate, Gelatin | Gelofusin, B. Braun, Germany | hemodynamic parameters |
| Soares et al, 2009 | cardiac surgery | Saline, gelatin |  | hemodynamic parameters and other clinical outcomes |
| Gondos et al, 2010 | mainly abdominal, cardiac surgery | Ringer's lactate, Albumin, L-HES, Gelatin | 5% human albumin, 130/0.4 6% HES, 4% succinylated gelatin | hemodynamic parameters and other clinical outcomes |
| Magder et al. 2010 | cardiac surgery | Saline, H-HES | 10% 250 pentastarch | hemodynamic parameters and other clinical outcomes |
| Hung et al, 2012 | abdominal surgery | Ringer's lactate, L-HES | 6% 130/0.4 HES | hemodynamic parameters and other clinical outcomes |
| Hamaji et al. 2013 | hip arthroplasty | Ringer's lactate,L-HES | 6% 130/0.4 HES | hemodynamic parameters and other clinical outcomes |
| Yates et al, 2014 | colorectal surgery | Hartmann's, L-HES | 6% 130/0.4 HES | gastrointestinal morbidities and other clinical outcomes |
| Yanartas et al, 2015 | coronary artery bypass surgery | Ringer's lactate, L-HES | 6% 130/0.4 HES | hemodynamic parameters and other clinical outcomes |
| Young et al, 2015 | cardiac surgery | Plasma-Lyte 148, Saline |  | renal outcome |

eTable 5.5. Study characteristics in surgical patients (continued)

| Kammerer et al, 2018 | elective cystectomy | Albumin, L-HES | 5% albumin; 6% 130/0.4 HES | renal outcome |
| --- | --- | --- | --- | --- |
| Joosten et al, 2018 | abdominal surgery | Plasmalyte, L-HES | Volulyte | Post-Operative Morbidity Survey score |
| Matthew W. et al, 2018 | surgical ICU | Balanced crystalloids, Saline |  | Mortality and acute kidney injury. |
| Futier et al, 2020 | Abdominal surgery | Saline, L-HES | 6% 130/0.4 HES | Mortality and complications |

### 5.2.2 Baseline characteristics, resuscitation goal and fluid volume

eTable 5.6. Patient’s characteristics in surgical trials

| Author, Year | Fluid resuscitation settings | case | age | male | fluid volume |
| --- | --- | --- | --- | --- | --- |
| Boutros et al, 1979 | post-operation | 24 | 61.3/64.5/60.3 | 62.5 | 2691/2205/2066 |
| Virgilio et al, 1979 | during operation to maintain PCWP | 29 | - | - | 11000/6200 |
| Zetterstrom et al, 1981 | during and after operation to maintain mean arterial occlusion pressure | 18 | - | - | - |
| Shires et al, 1983 | during operation to maintain cardiac output, PCWP, urine output | 18 | 64/67 | 78/78 | 8400/4800 |
| Prien et al, 1990 | during operation to maintain CVP | 18 | 52.7/60.8/62.1 | 33.3 | 3850/463/1358 |
| Wahba et al, 1996 | during operation | 22 | 62.3/66.3 | 60/80 | 5882/4010 |
| Waters et al, 2001 | during operation to maintain CVP within 10% of the baseline preoperative value | 66 | 69.9/69.8 | - | 6871/7000 |
| Linden et al, 2003 | during operation to maintain cardiac index, mixed venous oxygen saturation, and urine output | 110 | 63/63 | 64/67 | 4940/4920 |
| Linden et al, 2005 | during operation to maintain PCWP, cardiac index, and urine output | 132 | 67/66 | 77/71 | 1682/1638 |
| Mahmood et al. 2007 | during and after operation to maintain mean blood pressure and CVP level | 61 | 72/72/73 | 90/76/75 | 3911/3443/4490 |
| Tamayo et al, 2008 | for pump priming | 44 | 66.5/67.8 | 77/86 | 2577/2325 |
| Soares et al, 2009 | during and after operation to maintain CVP level | 40 | 67/66 | 75/65 | 3550/2555 |
| Gondos et al, 2010 | after operation | 200 | 59/53/58/60 | 42/48/60/52 | 10ml/kg |
| Magder et al. 2010 | after operation | 262 | 65.9/65.5 | 69.5/74.8 | 3275/2619 |
| Hung et al, 2012 | during operation to maintain PCWP, cardiac index, and urine output | 80 | 50.4/48 | 64.1/56.1 | 1760/999 |
| Hamaji et al. 2013 | during operation to maintain PCWP, cardiac index, and urine output | 48 | 53/54 | 37.5/58.3 | 30ml/kg, 15ml/kg |
| Yates et al, 2014 | during operation to maintain hemodynamics | 202 | 70/72 | 55/61 | 2500/1750 |
| Yanartas et al, 2015 | for pump priming | 132 | 61.3/62.5 | 33.3/42.4 | 1500/1500 |
| Young et al, 2015 | during operation to maintain hemodynamics | 960 | 60/61 | 64/67 | 1200/1000 |
| Kammerer et al, 2018 | during operation to maintain hemodynamics | 100 | 70/70 | 40/41 | 1705/2000 |
| Joosten et al, 2018 | during operation to maintain stroke volume, MAP | 160 | 62/65 | 51/45 | 4000/2900 |
| Matthew W. et al, 2018 | during ICU admission to maintain hemodynamic parameters | 1305 | 58/58 | 57.2/58 | 2117/2775 |
| Futier et al, 2020 | during operation to maintain hemodynamics | 775 | 69/68 | 89/87 | 1250/1000 |

## 5.3. Traumatic patients

### 5.3.1 Population, intervention, outcome

eTable 5.7. Study characteristics in traumatic patients

| Author, Year | Study population | Interventions | Colloid fluid detail | Outcome |
| --- | --- | --- | --- | --- |
| **Trauma** | | | | |
| Shah et al, 1977 | Laparotomy for acute abdominal trauma | Ringer's lactate, albumin | 5% albumin | hemodynamic parameters and other clinical outcomes |
| Lowe et al, 1979 | Laparotomy for acute abdominal trauma | Ringer's lactate, albumin | 25% albumin | hemodynamic parameters and other clinical outcomes |
| Nagy et al, 1993 |  | Ringer's lactate, H-HES | 10F% 200/0.5 pentastarch |  |
| Allison et al, 1999 | Blunt trauma patient. | H-HES, gelatin | 250/0.45 Pentaspan, Geistlich Pharma, UK; Gelofusine, Braun, UK | hemodynamic parameters and other clinical outcomes |
| Finfer et al, 2004 | Tramatic patients requiring fluid resuscitation. | Saline, Albumin | 4% albumin, CSL | mortality |
| James et al, 2011 | Blunt and penetrating trauma patients requiring >3 liters of fluid resuscitation. | Saline, L-HES | 130/0.4 Starch | mortality |
| Myburgh et al, 2012 | Trauma patients requring fluid resuscitation. | Saline, L-HES | 130/0.4 Starch | mortality |
| Young et al, 2014 | Trauma patients requring fluid resuscitation. | Plasma-Lyte, Saline, |  | mortality |
| Young et al, 2015 | Trauma patients requring fluid resuscitation. | Plasma-Lyte 148, Saline |  | mortality |
| Matthew W. et al, 2018 | Trauma patients requring fluid resuscitation. | Balanced crystalloids, Saline |  | mortality |
| **Traumatic brain injury** | | | | |
| SAFE study, 2007 | Trauma with brain injury. | Saline, Albumin | 4% albumin, CSL | mortality |
| Myburgh et al, 2012 | Trauma with brain injury. | Saline, L-HES |  | mortality |
| Young et al, 2015 | Trauma with brain injury. | Plasma-Lyte 148, Saline |  | mortality |
| Matthew W. et al, 2018 | Trauma with brain injury. | Balanced crystalloids, Saline |  | mortality |

### 5.3.2 Baseline characteristics and fluid volume

eTable 5.8 Patient’s characteristics in traumatic trials

| Author, Year | Interventions | case | age | male | fluid volume |
| --- | --- | --- | --- | --- | --- |
| Trauma | | | | | |
| Shah et al, 1977 | Ringer's lactate, albumin | 17 | 46.5/54 | 62/75 | 16400/7100 |
| Lowe et al, 1979 | Ringer's lactate, albumin | 137 | 27.6/32.3 | 89/94 | 5370/5870 |
| Nagy et al, 1993 | Ringer's lactate, H-HES | 41 |  |  |  |
| Allison et al, 1999 | H-HES, gelatin | 59 | 44/32.3 | 58/76 | 2744/3132 |
| Finfer et al, 2004 | Saline, Albumin | 694 | 58.5/58.6 | 61/59 | 1563/1184 |
| James et al, 2011 | Saline, L-HES | 109 | 34.1/30.3 | 79.2/85.7 | 6884/5603 |
| Myburgh et al, 2012 | Saline, L-HES | 521 | 62.9/63.1 | 60/61 | 1377/1731 |
| Young et al, 2014 | Plasma-Lyte, Saline, | 65 | 38/39 | 73/79 | 15600/14000 |
| Young et al, 2015 | Plasma-Lyte 148, Saline | 65 | 69/70 | 64/67 | 2655/2554 |
| Matthew W. et al, 2018 | Balanced crystalloids, Saline | 3328 | 58/58 | 57/58 | 2117/2775 |
| Traumatic brain injury | | | | | |
| Finfer et al, 2004 | Saline, Albumin | 492 | 58.5/58.6 | 61/59 | 1563/1184 |
| Myburgh et al, 2012 | Saline, L-HES | 57 | 62.9/63.1 | 60/61 | 1377/1731 |
| Young et al, 2015 | Plasma-Lyte 148, Saline | 58 | 69/70 | 64/67 | 2655/2554 |
| Matthew W. et al, 2018 | Balanced crystalloids, Saline | 1363 | 58/58 | 57/58 | 2117/2775 |

## 5.4. References

Referenced to studies included in this review

- **Sepsis patients, listed by year**

**Rackow et al, 1983**

Rackow EC, Falk JL, Fein IA, Siegel JS, Packman MI, Haupt MT, Kaufman BS, Putnam D. Fluid resuscitation in circulatory shock: a comparison of the cardiorespiratory effects of albumin, hetastarch, and saline solutions in patients with hypovolemic and septic shock. Crit Care Med. 1983 Nov;11(11):839-50. PMID: 6194934

**Rackow et al, 1989**

Rackow EC, Mecher C, Astiz ME, Griffel M, Falk JL, Weil MH. Effects of pentastarch and albumin infusion on cardiorespiratory function and coagulation in patients with severe sepsis and systemic hypoperfusion. Crit Care Med. 1989 May;17(5):394-8. PMID: 2468447

**Asfar et al, 2000**

Asfar P, Kerkeni N, Labadie F, Gouëllo JP, Brenet O, Alquier P. Assessment of hemodynamic and gastric mucosal acidosis with modified fluid versus 6% hydroxyethyl starch: a prospective, randomized study. Intensive Care Med. 2000 Sep;26(9):1282-7. PMID: 11089754

**Schortgen et al, 2001**

Schortgen F, Lacherade JC, Bruneel F, Cattaneo I, Hemery F, Lemaire F, Brochard L. Effects of hydroxyethylstarch and gelatin on renal function in severe sepsis: a multicentre randomised study. Lancet. 2001 Mar 24;357(9260):911-6. PMID: 11289347

**Finfer et al, 2004 (SAFE)**

Finfer S, Bellomo R, Boyce N, French J, Myburgh J, Norton R; SAFE Study Investigators. A comparison of albumin and saline for fluid resuscitation in the intensive care unit. N Engl J Med. 2004 May 27;350(22):2247-56. PMID: 15163774

**Molnar et al, 2004**

Molnár Z1, Mikor A, Leiner T, Szakmány T. Fluid resuscitation with colloids of different molecular weight in septic shock. Intensive Care Med. 2004 Jul;30(7):1356-60. PMID: 15127186

**Veneman et al, 2004**

Veneman TF1, Oude Nijhuis J, Woittiez AJ. Human albumin and starch administration in critically ill patients: a prospective randomized clinical trial. Wien Klin Wochenschr. 2004 May 31;116(9-10):305-9. PMID: 15237655

**Palumbo et al, 2006**

Palumbo D, Servillo G, D'Amato L, Volpe ML, Capogrosso G, De Robertis E, Piazza O, Tufano R. The effects of hydroxyethyl starch solution in critically ill patients. Minerva Anestesiol. 2006 Jul-Aug;72(7-8):655-64. PMID: 16865084

**Brunkhorst et al, 2008 (VISEP)**

Brunkhorst FM, Engel C, Bloos F, Meier-Hellmann A, Ragaller M, Weiler N, Moerer O, Gruendling M, Oppert M, Grond S, Olthoff D, Jaschinski U, John S, Rossaint R, Welte T, Schaefer M, Kern P, Kuhnt E, Kiehntopf M, Hartog C, Natanson C, Loeffler M, Reinhart K; German Competence Network Sepsis (SepNet). Intensive insulin therapy and pentastarch resuscitation in severe sepsis. N Engl J Med. 2008 Jan 10;358(2):125-39. PMID: 18184958

**Friedman et al, 2008**

Friedman G, Jankowski S, Shahla M, Gomez J, Vincent JL. Hemodynamic effects of 6% and 10% hydroxyethyl starch solutions versus 4% albumin solution in septic patients. J Clin Anesth. 2008 Nov;20(7):528-33. PMID: 19019650

**Li et al, 2008**

Li F, Sun H, Han XD. The effect of different fluids on early fluid resuscitation in septic shock, Zhongguo Wei Zhong Bing Ji Jiu Yi Xue. 2008 Aug;20(8):472-5. PMID: 18687174.

**McIntyre et al, 2008 (FINESS)**

McIntyre LA1, Fergusson D, Cook DJ, Rankin N, Dhingra V, Granton J, Magder S, Stiell I, Taljaard M, Hebert PC; Canadian Critical Care Trials Group. Fluid resuscitation in the management of early septic shock (FINESS): a randomized controlled feasibility trial. Can J Anaesth. 2008 Dec;55(12):819-26. PMID: 19050085

**Dolecek et al, 2009**

Dolecek M1, Svoboda P, Kantorová I, Scheer P, Sas I, Bíbrová J, Radvanova J, Radvan M. Therapeutic influence of 20 % albumin versus 6% hydroxyethylstarch on extravascular lung water in septic patients: a randomized controlled trial. Hepatogastroenterology. 2009 Nov-Dec;56(96):1622-8. PMID: 20214205

**Dubin et al, 2010**

Dubin A, Pozo MO, Casabella CA, Murias G, Pálizas F Jr, Moseinco MC, Kanoore Edul VS, Pálizas F, Estenssoro E, Ince C. Comparison of 6% hydroxyethyl starch 130/0.4 and saline solution for resuscitation of the microcirculation during the early goal-directed therapy of septic patients. J Crit Care. 2010 Dec;25(4):659.e1-8. PMID: 20813485

**Charpentier et al, 2011 (EARSS)**

Charpentier J, Mira JP. Efficacy and tolerance of hyperoncolit albumin administration in septic shock patients: The EARSS study. Intensive Care Med 37 Suppl 1: S6–314.

**Siegemund, 2011 (BaSES)**

Siegemund M. BaSES Trial: Basel Starch Evaluation in Sepsis. ClinicalTrials.gov 2012; http://clinicaltrials.gov/ct2/show/study/NCT00273728. Accessed October 31, 2012

**Guidet et al, 2012 (CRYSTMAS)**

Guidet B, Martinet O, Boulain T, Philippart F, Poussel JF, Maizel J, Forceville X, Feissel M, Hasselmann M, Heininger A, Van Aken H. Assessment of hemodynamic efficacy and safety of 6% hydroxyethylstarch 130/0.4 vs. 0.9% NaCl fluid replacement in patients with severe sepsis: the CRYSTMAS study. Crit Care. 2012 May 24;16(3):R94. PMID: 22624531

**Myburgh et al, 2012 (CHEST)**

Myburgh JA, Finfer S, Bellomo R, Billot L, Cass A, Gattas D, Glass P, Lipman J, Liu B, McArthur C, McGuinness S, Rajbhandari D, Taylor CB, Webb SA; CHEST Investigators; Australian and New Zealand Intensive Care Society Clinical Trials Group. Hydroxyethyl starch or saline for fluid resuscitation in intensive care. N Engl J Med. 2012 Nov 15;367(20):1901-11. PMID: 23075127

**Perner et al, 2012 (6S)**

Perner A, Haase N, Guttormsen AB, Tenhunen J, Klemenzson G, Åneman A, Madsen KR, Møller MH, Elkjær JM, Poulsen LM, Bendtsen A, Winding R, Steensen M, Berezowicz P, Søe-Jensen P, Bestle M, Strand K, Wiis J, White JO, Thornberg KJ, Quist L, Nielsen J, Andersen LH, Holst LB, Thormar K, Kjældgaard AL, Fabritius ML, Mondrup F, Pott FC, Møller TP, Winkel P, Wetterslev J; 6S Trial Group; Scandinavian Critical Care Trials Group. Hydroxyethyl starch 130/0.42 versus Ringer's acetate in severe sepsis. N Engl J Med. 2012 Jul 12;367(2):124-34. PMID: 22738085

**Annane et al, 2013 (CRISTAL)**

Annane D, Siami S, Jaber S, Martin C, Elatrous S, Declère AD, Preiser JC, Outin H, Troché G, Charpentier C, Trouillet JL, Kimmoun A, Forceville X, Darmon M, Lesur O, Reignier J, Abroug F, Berger P, Clec'h C, Cousson J, Thibault L, Chevret S; CRISTAL Investigators. Effects of fluid resuscitation with colloids vs crystalloids on mortality in critically ill patients presenting with hypovolemic shock: the CRISTAL randomized trial. JAMA. 2013 Nov 6;310(17):1809-17. PMID: 24108515

**Caironi et al, 2014 (ALBIOS)**

Caironi P, Tognoni G, Masson S, Fumagalli R, Pesenti A, Romero M, Fanizza C, Caspani L, Faenza S, Grasselli G, Iapichino G, Antonelli M, Parrini V, Fiore G, Latini R, Gattinoni L; ALBIOS Study Investigators. Albumin replacement in patients with severe sepsis or septic shock. N Engl J Med. 2014 Apr 10;370(15):1412-21. PMID: 24635772

**Young et al, 2015 (SPLIT)**

Young P, Bailey M, Beasley R, Henderson S, Mackle D, McArthur C, McGuinness S, Mehrtens J, Myburgh J, Psirides A, Reddy S, Bellomo R; SPLIT Investigators; ANZICS CTG. Effect of a Buffered Crystalloid Solution vs Saline on Acute Kidney Injury Among Patients in the Intensive Care Unit: The SPLIT Randomized Clinical Trial. JAMA. 2015 Oct 27;314(16):1701-10. PMID: 26444692

**Matthew W. et al, 2018 (SMART)**

Semler MW, Self WH, Wanderer JP, Ehrenfeld JM, Wang L, Byrne DW, Stollings JL, Kumar AB, Hughes CG, Hernandez A, Guillamondegui OD, May AK, Weavind L, Casey JD, Siew ED, Shaw AD, Bernard GR, Rice TW; SMART Investigators and the Pragmatic Critical Care Research Group.Balanced Crystalloids versus Saline in Critically Ill Adults. N Engl J Med. 2018 Mar 1;378(9):829-839. PMID: 29485925

- **Surgical patients, listed by year**

**Boutros et al, 1979**

Boutros AR, Ruess R, Olson L, Hoyt JL, Baker WH. Comparison of hemodynamic, pulmonary, and renal effects of use of three types of fluids after major surgical procedures on the abdominal aorta. Crit Care Med. 1979 Jan;7(1):9-13. PMID: 367709

**Virgilio et al, 1979**

Virgilio RW, Rice CL, Smith DE, James DR, Zarins CK, Hobelmann CF, Peters RM. Crystalloid vs. colloid resuscitation: is one better? A randomized clinical study. Surgery. 1979 Feb;85(2):129-39. PMID: 419454

**Zetterstrom et al, 1981**

Zetterström H. Albumin treatment following major surgery. II. Effects on postoperative lung function and circulatory adaptation. Acta Anaesthesiol Scand. 1981 Apr;25(2):133-41. PMID: 7324820

**Shires et al, 1983**

Shires GT 3rd, Peitzman AB, Albert SA, Illner H, Silane MF, Perry MO, Shires GT. Response of extravascular lung water to intraoperative fluids. Ann Surg. 1983 May;197(5):515-9. PMID: 6847271

**Prien et al, 1990**

Prien T, Backhaus N, Pelster F, Pircher W, Bünte H, Lawin P. Effect of intraoperative fluid administration and colloid osmotic pressure on the formation of intestinal edema during gastrointestinal surgery. J Clin Anesth. 1990 Sep-Oct;2(5):317-23. PMID: 1702977

**Wahba et al, 1996**

Wahba A1, Sendtner E, Strotzer M, Wild K, Birnbaum DE. Fluid therapy with Ringer's solution versus Haemaccel following coronary artery bypass surgery. Acta Anaesthesiol Scand. 1996 Nov;40(10):1227-33. PMID: 8986187

**Waters et al, 2001**

Waters JH, Gottlieb A, Schoenwald P, Popovich MJ, Sprung J, Nelson DR. Saline versus lactated Ringer's solution for intraoperative fluid management in patients undergoing abdominal aortic aneurysm repair: an outcome study. Anesth Analg. 2001 Oct;93(4):817-22. PMID: 11574339

**Linden et al, 2003**

Van der Linden PJ1, De Hert SG, Daper A, Trenchant A, Schmartz D, Defrance P, Kimbimbi P. 3.5% urea-linked gelatin is as effective as 6% HES 200/0.5 for volume management in cardiac surgery patients. Can J Anaesth. 2004 Mar;51(3):236-41. PMID: 15010405

**Linden et al, 2005**

Van der Linden PJ, De Hert SG, Deraedt D, Cromheecke S, De Decker K, De Paep R, Rodrigus I, Daper A, Trenchant A. Hydroxyethyl starch 130/0.4 versus modified fluid gelatin for volume expansion in cardiac surgery patients: the effects on perioperative bleeding and transfusion needs. Anesth Analg. 2005 Sep;101(3):629-34. PMID: 16115963

**Mahmood et al. 2007**

Mahmood A1, Gosling P, Vohra RK. Randomized clinical trial comparing the effects on renal function of hydroxyethyl starch or gelatine during aortic aneurysm surgery. Br J Surg. 2007 Apr;94(4):427-33. PMID: 17380548

**Tamayo et al, 2008**

Tamayo E1, Alvarez FJ, Alonso O, Castrodeza J, Bustamante R, Gómez-Herreras JI, Florez S, Rodríguez R. The inflammatory response to colloids and crystalloids used for pump priming during cardiopulmonary bypass. Acta Anaesthesiol Scand. 2008 Oct;52(9):1204-12. PMID: 18823458

**Soares et al, 2009**

Soares RR1, Ferber L, Lorentz MN, Soldati MT. Intraoperative volume replacement: crystalloids versus colloids in surgical myocardial revascularization without cardiopulmonary bypass. Rev Bras Anestesiol. 2009 Jul-Aug;59(4):439-51. PMID: 19669018

**Gondos et al, 2010**

Gondos T, Marjanek Z, Ulakcsai Z, Szabó Z, Bogár L, Károlyi M, Gartner B, Kiss K, Havas A, Futó J. Short-term effectiveness of different volume replacement therapies in postoperative hypovolaemic patients. Eur J Anaesthesiol. 2010 Sep;27(9):794-800. PMID: 20520555

**Magder et al. 2010**

Magder S, Potter BJ, Varennes BD, Doucette S, Fergusson D; Canadian Critical Care Trials Group. Fluids after cardiac surgery: a pilot study of the use of colloids versus crystalloids. Crit Care Med. 2010 Nov;38(11):2117-24. PMID: 20802322

**Hung et al, 2012**

Hung MH, Zou C, Lin FS, Lin CJ, Chan KC, Chen Y. New 6% hydroxyethyl starch 130/0.4 does not increase blood loss during major abdominal surgery - a randomized, controlled trial. J Formos Med Assoc. 2014 Jul;113(7):429-35. PMID: 24961184

**Hamaji et al. 2013**

Hamaji A, Hajjar L, Caiero M, Almeida J, Nakamura RE, Osawa EA, Fukushima J, Galas FR, Auler JO Jr. Volume replacement therapy during hip arthroplasty using hydroxyethyl starch (130/0.4) compared to lactated Ringer decreases allogeneic blood transfusion and postoperative infection. Braz J Anesthesiol. 2013 Jan;63(1):27-35. PMID: 24565088

**Yates et al, 2014**

Yates DR, Davies SJ, Milner HE, Wilson RJ. Crystalloid or colloid for goal-directed fluid therapy in colorectal surgery. Br J Anaesth. 2014 Feb;112(2):281-9. PMID: 24056586

**Yanartas et al, 2015**

Yanartas M, Baysal A, Aydın C, Ay Y, Kara I, Aydın E, Cevirme D, Köksal C, Sunar H. The effects of tranexamic acid and 6% hydroxyethyl starch (HES) solution (130/0.4) on postoperative bleeding in coronary artery bypass graft (CABG) surgery. Int J Clin Exp Med. 2015 Apr 15;8(4):5959-71. PMID: 26131192

**Young et al, 2015 (SPLIT)**

Young P, Bailey M, Beasley R, Henderson S, Mackle D, McArthur C, McGuinness S, Mehrtens J, Myburgh J, Psirides A, Reddy S, Bellomo R; SPLIT Investigators; ANZICS CTG. Effect of a Buffered Crystalloid Solution vs Saline on Acute Kidney Injury Among Patients in the Intensive Care Unit: The SPLIT Randomized Clinical Trial. JAMA. 2015 Oct 27;314(16):1701-10. PMID: 26444692

**Kammerer et al, 2018**

Kammerer T, Brettner F, Hilferink S, Hulde N, Klug F, Pagel JI, Karl A, Crispin A, Hofmann-Kiefer K, Conzen P, Rehm M. No Differences in Renal Function between Balanced 6% Hydroxyethyl Starch (130/0.4) and 5% Albumin for Volume Replacement Therapy in Patients Undergoing Cystectomy: A Randomized Controlled Trial. Anesthesiology. 2018 Jan;128(1):67-78. PMID: 29064872

**Joosten et al, 2018**

Joosten A, Delaporte A, Ickx B, Touihri K, Stany I, Barvais L, Van Obbergh L, Loi P, Rinehart J, Cannesson M, Van der Linden P. Crystalloid versus Colloid for Intraoperative Goal-directed Fluid Therapy Using a Closed-loop System: A Randomized, Double-blinded, Controlled Trial in Major Abdominal Surgery. Anesthesiology. 2018 Jan;128(1):55-66. PMID: 29068831

**Matthew W. et al, 2018**

Semler MW, Self WH, Wanderer JP, Ehrenfeld JM, Wang L, Byrne DW, Stollings JL, Kumar AB, Hughes CG, Hernandez A, Guillamondegui OD, May AK, Weavind L, Casey JD, Siew ED, Shaw AD, Bernard GR, Rice TW; SMART Investigators and the Pragmatic Critical Care Research Group.Balanced Crystalloids versus Saline in Critically Ill Adults. N Engl J Med. 2018 Mar 1;378(9):829-839. PMID: 29485925

**Futier et al, 2020 (FLASH)**

Futier E, Garot M, Godet T, et al. Effect of Hydroxyethyl Starch vs Saline for Volume Replacement Therapy on Death or Postoperative Complications Among High-Risk Patients Undergoing Major Abdominal Surgery: The FLASH Randomized Clinical Trial. *JAMA.* 2020;323(3):225-236.

- **Traumatic patients, listed by year**

**Shah et al, 1977**

Shah DM, Browner BD, Dutton RE, Newell JC, Powers SR Jr. Cardiac output and pulmonary wedge pressure. Use for evaluation of fluid replacement in trauma patients. Arch Surg. 1977 Oct;112(10):1161-8. PMID: 907460

**Lowe et al, 1979**

Lowe RJ, Moss GS, Jilek J, Levine HD. Crystalloid versus colloid in the etiology of pulmonary failure after trauma--a randomized trial in man. Crit Care Med. 1979 Mar;7(3):107-12. PMID: 436425

**Nagy et al, 1993**

Nagy KK, Davis J, Duda J, Fildes J, Roberts R, Barrett J. A comparison of pentastarch and lactated Ringer's solution in the resuscitation of patients with hemorrhagic shock. Circ Shock. 1993 Aug;40(4):289-94. PMID: 7690689

**Allison et al, 1999**

Allison KP, Gosling P, Jones S, Pallister I, Porter KM. Randomized trial of hydroxyethyl starch versus gelatine for trauma resuscitation. J Trauma. 1999 Dec;47(6):1114-21. PMID: 10608543

**SAFE study investigators, 2007 (SAFE)**

SAFE study investigators. Saline or albumin for fluid resusucitation in patients with traumatic brain injury. N Engl J Med. 2007 Aug 39;357(9):874-84. PMID: 17761591

**James et al, 2011**

James MF, Michell WL, Joubert IA, Nicol AJ, Navsaria PH, Gillespie RS. Resuscitation with hydroxyethyl starch improves renal function and lactate clearance in penetrating trauma in a randomized controlled study: the FIRST trial (Fluids in Resuscitation of Severe Trauma). Br J Anaesth. 2011 Nov;107(5):693-702. PMID: 21857015

**Myburgh et al, 2012 (CHEST)**

Myburgh JA, Finfer S, Bellomo R, Billot L, Cass A, Gattas D, Glass P, Lipman J, Liu B, McArthur C, McGuinness S, Rajbhandari D, Taylor CB, Webb SA; CHEST Investigators; Australian and New Zealand Intensive Care Society Clinical Trials Group. Hydroxyethyl starch or saline for fluid resuscitation in intensive care. N Engl J Med. 2012 Nov 15;367(20):1901-11. PMID: 23075127

**Young et al, 2014**

Young JB, Utter GH, Schermer CR, Galante JM, Phan HH, Yang Y, Anderson BA, Scherer LA. Saline versus Plasma-Lyte A in initial resuscitation of trauma patients: a randomized trial. Ann Surg. 2014 Feb;259(2):255-62. PMID: 23732264

**Young et al, 2015 (SPLIT)**

Young P, Bailey M, Beasley R, Henderson S, Mackle D, McArthur C, McGuinness S, Mehrtens J, Myburgh J, Psirides A, Reddy S, Bellomo R; SPLIT Investigators; ANZICS CTG. Effect of a Buffered Crystalloid Solution vs Saline on Acute Kidney Injury Among Patients in the Intensive Care Unit: The SPLIT Randomized Clinical Trial. JAMA. 2015 Oct 27;314(16):1701-10. PMID: 26444692

**Matthew W. et al, 2018**

Semler MW, Self WH, Wanderer JP, Ehrenfeld JM, Wang L, Byrne DW, Stollings JL, Kumar AB, Hughes CG, Hernandez A, Guillamondegui OD, May AK, Weavind L, Casey JD, Siew ED, Shaw AD, Bernard GR, Rice TW; SMART Investigators and the Pragmatic Critical Care Research Group.Balanced Crystalloids versus Saline in Critically Ill Adults. N Engl J Med. 2018 Mar 1;378(9):829-839. PMID: 29485925

- **Traumatic brain injury patients, listed by year**

**Finfer et al, 2004 (SAFE)**

Finfer S, Bellomo R, Boyce N, French J, Myburgh J, Norton R; SAFE Study Investigators. A comparison of albumin and saline for fluid resuscitation in the intensive care unit. N Engl J Med. 2004 May 27;350(22):2247-56. PMID: 15163774

**Myburgh et al, 2012 (CHEST)**

Myburgh JA, Finfer S, Bellomo R, Billot L, Cass A, Gattas D, Glass P, Lipman J, Liu B, McArthur C, McGuinness S, Rajbhandari D, Taylor CB, Webb SA; CHEST Investigators; Australian and New Zealand Intensive Care Society Clinical Trials Group. Hydroxyethyl starch or saline for fluid resuscitation in intensive care. N Engl J Med. 2012 Nov 15;367(20):1901-11. PMID: 23075127

**Young et al, 2015 (SPLIT)**

Young P, Bailey M, Beasley R, Henderson S, Mackle D, McArthur C, McGuinness S, Mehrtens J, Myburgh J, Psirides A, Reddy S, Bellomo R; SPLIT Investigators; ANZICS CTG. Effect of a Buffered Crystalloid Solution vs Saline on Acute Kidney Injury Among Patients in the Intensive Care Unit: The SPLIT Randomized Clinical Trial. JAMA. 2015 Oct 27;314(16):1701-10. PMID: 26444692

**Matthew W. et al, 2018**

Semler MW, Self WH, Wanderer JP, Ehrenfeld JM, Wang L, Byrne DW, Stollings JL, Kumar AB, Hughes CG, Hernandez A, Guillamondegui OD, May AK, Weavind L, Casey JD, Siew ED, Shaw AD, Bernard GR, Rice TW; SMART Investigators and the Pragmatic Critical Care Research Group.Balanced Crystalloids versus Saline in Critically Ill Adults. N Engl J Med. 2018 Mar 1;378(9):829-839. PMID: 29485925

# Appendix 6: Assessment of transitivity

Before statistical analysis, we assessed the transitivity assumption by checking the included trials in the NMA were on average similar in terms of characteristics that might modify the treatment effect. Those characteristics in sepsis trials included

- Age
- Male percentage
- Sample size
- APACHE
- SAPS
- SOFA
- Mean arterial pressure
- Lactate level
- Vasopressor
- Source of sepsis from lung (pneumonia)
- Year

## 6.1. Age

| Age | Balanced  crystalloids | 0.9%  saline | Iso-oncotic  albumin | Hyperoncotic  albumin | L-HES | H-HES | Gelatin |
| --- | --- | --- | --- | --- | --- | --- | --- |
| Mean | 62.6 | 63.1 | 67.4 | 62.9 | 62.7 | 60.4 | 60.0 |
| SD | 3.7 | 6.3 | 11.5 | 9.4 | 7.6 | 8.0 | 11.3 |
| P value | Reference | 0.909 | 0.399 | 0.948 | 0.989 | 0.641 | 0.647 |

Regarding mean age, the range was 60.0 to 64.4, no statistically nor clinically significant different among 7 interventions.

## 6.2. Male percentage

| Male percentage | Balanced  crystalloids | 0.9%  saline | Iso-oncotic  albumin | Hyperoncotic  albumin | L-HES | H-HES | Gelatin |
| --- | --- | --- | --- | --- | --- | --- | --- |
| Mean | 61.0 | 62.9 | 69.7 | 58.6 | 58.8 | 64.6 | 62.0 |
| SD | 4.2 | 6.4 | 11.6 | 10.4 | 8.1 | 8.1 | 11.5 |
| P value | Reference | 0.693 | 0.164 | 0.682 | 0.670 | 0.493 | 0.872 |

Regarding mean male percentage, the range was 58.8 to 64.6, no statistically significant different among 7 interventions.

## 6.3. Sample size

| Sample siza | Balanced  crystalloids | 0.9%  saline | Iso-oncotic  albumin | Hyperoncotic  albumin | L-HES | H-HES | Gelatin |
| --- | --- | --- | --- | --- | --- | --- | --- |
| Mean | 696.2 | 457.0 | 158.7 | 236.0 | 205.0 | 53.7 | 62.5 |
| SD | 247.7 | 158.7 | 300.1 | 245.0 | 187.8 | 209.9 | 296.9 |
| p-value | Reference | 0.411 | 0.159 | 1.181 | 0.112 | 0.048 | 0.095 |

Regarding the mean sample size, the range was 53.7 to 696.2, the sample size was significantly less in H-HES than in BC.

## 6.4. APACHE

| APACHE | Balanced  crystalloids | 0.9%  saline | Iso-oncotic  albumin | Hyperoncotic  albumin | L-HES | H-HES | Gelatin |
| --- | --- | --- | --- | --- | --- | --- | --- |
| Mean | 17.2 | 18.0 | 18.0 | 20.8 | 19.7 | 18.8 | Not available |
| SD | 1.9 | 2.3 | 7.5 | 5.3 | 2.6 | 2.6 |  |
| P value | Reference | 0.732 | 0.822 | 0.225 | 0.353 | 0.543 |  |

Regarding mean APACHE score, the range was 17.2 to 19.9, no statistically significant different among interventions.

## 6.5. SAPS

| SAPS score | Balanced  crystalloids | 0.9%  saline | Iso-oncotic  albumin | Hyperoncotic  albumin | L-HES | H-HES | Gelatin |
| --- | --- | --- | --- | --- | --- | --- | --- |
| Mean | 50.5 | 50.3 | Not available | 48 | 49.3 | 46.3 | 44.5 |
| SD | 4.3 | 12.4 |  | 15.1 | 12.4 | 12.4 | 10.7 |
| P value | reference | 0.976 |  | 0.686 | 0.836 | 0.465 | 0.275 |

Regarding mean SAPS score, the range was 44.5 to 50.3, no statistically significant different among 6 interventions.

## 6.6. SOFA

| SOFA score | Balanced  crystalloids | 0.9%  saline | Iso-oncotic  albumin | Hyperoncotic  albumin | L-HES | H-HES | Gelatin |
| --- | --- | --- | --- | --- | --- | --- | --- |
| Mean | 6.0 | 8.6 | Not available | 8.5 | 7.4 | Not available | 8.0 |
| SD | 1.1 | 1.3 |  | 1.4 | 1.3 |  | 2.8 |
| P value | reference | 0.076 |  | 0.092 | 0.297 |  | 0.321 |

Regarding mean SOFA scores, the range was 6.0 to 8.6, no statistically significant different among 5 interventions.

## 6.7. Mean arterial pressure

| MAP | Balanced  crystalloids | 0.9%  saline | Iso-oncotic  albumin | Hyperoncotic  albumin | L-HES | H-HES | Gelatin |
| --- | --- | --- | --- | --- | --- | --- | --- |
| Mean | 70.5 | 69.0 | 71.1 | 75.4 | 69.1 | 71.6 | 73.9 |
| SD | 6.3 | 6.6 | 9.9 | 9.9 | 8.0 | 7.0 | 9.8 |
| P value | reference | 0.836 | 0.944 | 0.535 | 0.853 | 0.872 | 0.664 |

Regarding mean arterial pressure (MAP) during enrollment, the range was 69.0 to 73.9, no statistically nor clinically significant different among 7 groups.

## 6.8. Lactate level

| Lactate level | Balanced  crystalloids | 0.9%  saline | Iso-oncotic  albumin | Hyperoncotic  albumin | L-HES | H-HES | Gelatin |
| --- | --- | --- | --- | --- | --- | --- | --- |
| Mean | 20.4 | 25.7 | 52.2 | 13.8 | 27.0 | 44.6 | 20.7 |
| SD | 10.4 | 12.2 | 12.1 | 8.5 | 12.7 | 14.7 | 20.8 |
| P value | reference | 0.670 | 0.037 | 0.585 | 0.611 | 0.116 | 0.990 |

Regarding mean lactate level during enrollment, the range was 20.7 to 44.6, the iso-oncotic albumin group was significantly higher than balanced crystalloids group.

## 6.9. Vasopressor

| Vasopressor usage | Balanced  crystalloids | 0.9%  saline | Iso-oncotic  albumin | Hyperoncotic  albumin | L-HES | H-HES | Gelatin |
| --- | --- | --- | --- | --- | --- | --- | --- |
| Mean | 55.2 | 56.9 | 84.6 | 60 | 68.3 | 65.1 | 37.0 |
| SD | 17.0 | 12.4 | 31.1 | 31.1 | 15.2 | 13.6 | 21.5 |
| P value | reference | 0.931 | 0.343 | 0.875 | 0.541 | 0.631 | 0.461 |

Regarding vasopressor usage in enrolled patients, the range was 37% to 72.3%, no statistically significant different among 6groups.

## 6.10. Source of sepsis from lung (pneumonia)

| Pneumonia percentage | Balanced  crystalloids | 0.9%  saline | Iso-oncotic  albumin | Hyperoncotic  albumin | L-HES | H-HES | Gelatin |
| --- | --- | --- | --- | --- | --- | --- | --- |
| Mean | 49.0 | 46.6 | 25 | 41.9 | 58.5 | 33.4 | 38.0 |
| SD | 12.3 | 14.7 | 26.2 | 18.5 | 18.0 | 11.4 | 14.7 |
| P value | reference | 0.878 | 0.290 | 0.693 | 0.596 | 0.303 | 0.499 |

Regarding the source of sepsis, the pneumonia percentage was 33.4% to 58.5%, there was no statistically significant different among 6 interventions.

## 6.11. Year

| Age | Balanced  crystalloids | 0.9%  saline | Iso-oncotic  albumin | Hyperoncotic  albumin | L-HES | H-HES | Gelatin |
| --- | --- | --- | --- | --- | --- | --- | --- |
| Mean | 2013.2 | 2008.8 | 1996 | 2009.5 | 2008.2 | 2003.2 | 2004.5 |
| P value | reference | 0.309 | 0.002 | 0.432 | 0.190 | 0.039 | 0.122 |

Regarding study year, the iso-oncotic albumin and H-HES study was older.

# Appendix 7: Risk of bias

## 7.1. Sepsis patients

### 7.1.1. Risk of bias assessment for the individual domains in sepsis trials

eFigure 7.1: Risk of bias assessment for the individual domains in sepsis trials

### 7.1.2. Risk of bias assessment for the individual studies in sepsis trials

| Author, Year | Domain 1 | Domain 2 | Domain 3 | Domain 4 | Domain 5 | Overall Bias |  |  |
| --- | --- | --- | --- | --- | --- | --- | --- | --- |
| Rackow et al, 1983 |  |  |  |  |  |  |  | Domain :   1. Randomization process 2. Deviations from intended interventions 3. Missing outcome data 4. Measurement of the outcome 5. Selection of the reported result |
| Rackow et al, 1989 |  |  |  |  |  |  |  |  |
| Asfar et al, 2000 |  |  |  |  |  |  |  |  |
| Schortgen et al, 2001 |  |  |  |  |  |  |  |  |
| Finfer et al, 2004 |  |  |  |  |  |  |  |  |
| Molnar et al, 2004 |  |  |  |  |  |  |  |  |
| Veneman et al, 2004 |  |  |  |  |  |  |  |  |
| Palumbo et al, 2006 |  |  |  |  |  |  |  | Low risk |
| Burnkhorst et al, 2008 |  |  |  |  |  |  |  | Some concerns |
| Friedman et al, 2008 |  |  |  |  |  |  |  | High risk |
| Li et al, 2008 |  |  |  |  |  |  |  |  |
| McIntyre et al, 2008 |  |  |  |  |  |  |  |  |
| Dolecek et al, 2009 |  |  |  |  |  |  |  |  |
| Dubin et al, 2010 |  |  |  |  |  |  |  |  |
| Charpentier et al, 2011 |  |  |  |  |  |  |  |  |
| Siegemund, 2011 |  |  |  |  |  |  |  |  |
| Guidet et al, 2012 |  |  |  |  |  |  |  |  |
| Myburgh et al, 2012 |  |  |  |  |  |  |  |  |
| Perner et al, 2012 |  |  |  |  |  |  |  |  |
| Annane et al, 2013 |  |  |  |  |  |  |  |  |
| Caironi et al, 2014 |  |  |  |  |  |  |  |  |
| Young et al, 2015 |  |  |  |  |  |  |  |  |
| Matthew W. et al, 2018 |  |  |  |  |  |  |  |  |

eFigure 7.2: Risk of bias assessment for sepsis trials

### 7.1.3. Risk of bias notes for the individual studies in sepsis trials

eTable 7.1. Risk of bias details in sepsis trials

| Study ID | Notes for risk of bias assessment |
| --- | --- |
| Rackow et al, 1983 | Domain 1. Predetermined randomization schedule. Saline group was younger and had a higher percentage of women. |
|  | Domain 2. May consider using regression model to adjust baseline difference. |
| Rackow et al, 1989 | Domain 1. Randomly assigned to different treatment. (No detailed randomization methods were mentioned.) Younger and more male patients in albumin group. |
|  | Domain 5. This study was designed to observe the cardiorespiratory function and coagulation after fluid resuscitation. |
| Asfar et al, 2000 | Domain 2. Unblinded trial. |
|  | Domain 5. This study was designed to observe the hemodynamic changes after fluid resuscitation. |
| Schortgen et al, 2001 | Domain 1. Centralised randomisation was organised at the Henri Mondor University Hospital. Blocks were prepared separately for each treatment center. Sealed opaque envelops were serially numbered and used in sequence. |
|  | Domain 2. The difference in dose recommendations from manufacturer between the two plasma-volume expanders precluded double blinding. Hydroxyethylstarch: 33 mL/kg during the first day, 20 mL/kg daily thereafter, the duration was limited to 4 days, and the cumulative dose to 80 mL/kg (further fluid loading after this point were given a crystalloid solution). Gelatin : given as needed, with no dose limitation. |
| Molnar et al, 2004 | Domain 1. Patients were randomly allocated in a block-of-six fashion. |
| Veneman et al, 2004 | radomization with sealed envolop. Higher APACHEII score and lactate level in HES group and the mortality was also higher in HES group. |
| Palumbo et al, 2006 | Domain 1. Radomized sequence. |
| Burnkhorst et al, 2008 | Domain 2. Open label study. Subgroup analysis in supplementary files and dose response analysis in main text. |
| Friedman et al, 2008 | Domain 1. Sealed opaque envelop assignment. |
| Li et al, 2008 | Domain 1. Randomization process was not mentioned. |
| Dubin et al, 2010 | Domain 2. Only 57% of physicians and 54% of nurses correctly guessed the study fluid (P -- 0.46 and P = 0.67, respectively). |
| Charpentier et al, 2011 | Domain 2. Open label study. |
|  | Domain 3. No detailed infomation. |
| Siegemund, 2011 | Domain 3. No detailed follow-up data was reported. |
| Annane et al, 2013 | Domain 2. Multicenter trials, unblinded. In colloid group, 6.2% patients received 3000ml Ringer's lactate; in crystalloid group, 17.6% patients reveived 2000ml Ringer's lactate. The Ringer's lactate was not balanced in two groups. In our knowledge, isotonic saline and Ringer's lactate were differenct crystalloids, but the study design let the physicians to choose those crystalloids without restrictions. No analysis to deal with the two different crystalloids. |
| Matthew W. et al, 2018 | Domain 1. This is a pragmatic, cluster-randomized, multiple-crossover trial conducted in five intensive care units at an academic center. |

## 7.2 Surgical patients

### 7.2.1. Risk of bias assessment for studies in surgical trials

eFigure 7.3: Risk of bias assessment for the individual domains in surgical trials

### 7.2.2. Risk of bias assessment for the individual studies in surgical trials

| Author, Year | Domain 1 | Domain 2 | Domain 3 | Domain 4 | Domain 5 | Overall Bias |  |  |
| --- | --- | --- | --- | --- | --- | --- | --- | --- |
| Boutros et al, 1979 |  |  |  |  |  |  |  | Domain :   1. Randomization process 2. Deviations from intended interventions 3. Missing outcome data 4. Measurement of the outcome 5. Selection of the reported result |
| Virgilio et al, 1979 |  |  |  |  |  |  |  |  |
| Zetterstrom et al, 1981 |  |  |  |  |  |  |  |  |
| Shires et al, 1983 |  |  |  |  |  |  |  |  |
| Prien et al, 1990 |  |  |  |  |  |  |  |  |
| Wahba et al, 1996 |  |  |  |  |  |  |  |  |
| Waters et al, 2001 |  |  |  |  |  |  |  |  |
| Linden et al, 2003 |  |  |  |  |  |  |  | Low risk |
| Linden et al, 2005 |  |  |  |  |  |  |  | Some concerns |
| Mahmood et al. 2007 |  |  |  |  |  |  |  | High risk |
| Tamayo et al, 2008 |  |  |  |  |  |  |  |  |
| Soares et al, 2009 |  |  |  |  |  |  |  |  |
| Gondos et al, 2010 |  |  |  |  |  |  |  |  |
| Magder et al. 2010 |  |  |  |  |  |  |  |  |
| Hung et al, 2012 |  |  |  |  |  |  |  |  |
| Hamaji et al. 2013 |  |  |  |  |  |  |  |  |
| Yates et al, 2014 |  |  |  |  |  |  |  |  |
| Yanartas et al, 2015 |  |  |  |  |  |  |  |  |
| Young et al, 2015 |  |  |  |  |  |  |  |  |
| Kammerer et al, 2018 |  |  |  |  |  |  |  |  |
| Joosten et al, 2018 |  |  |  |  |  |  |  |  |
| Matthew W. et al, 2018 |  |  |  |  |  |  |  |  |
| Futier et al, 2020 |  |  |  |  |  |  |  |  |

eFigure 7.4: Risk of bias assessment for surgical trials

### 7.2.3. Risk of bias notes for the individual studies in surgical trials

eTable 7.2. Risk of bias details in surgical trials

| **Study ID** | **Notes for risk of bias assessment** |
| --- | --- |
| Boutros et al, 1979 | Domain 1. Allocation or randomization method was not mentioned. |
| Virgilio et al, 1979 | Domain 1. Allocation or randomization method was not mentioned. |
| Zetterstrom et al, 1981 | Domain 1. Allocation or randomization method was not mentioned. |
| Shires et al, 1983 | Domain 1. Allocation or randomization method was not mentioned. |
| Linden et al, 2003 | Domain 2. open labelled. |
| Linden et al, 2005 | Domain 2. open labelled. |
| Mahmood et al. 2007 | Domain 2. open labelled. |
| Tamayo et al, 2008 | Domain 2. open labelled. |
| Soares et al, 2009 | Domain 2. open labelled. |
| Hung et al, 2012 | Domain 2. open labelled. |
| Hamaji et al. 2013 | Domain 2. open labelled. |
| Yanartas et al, 2015 | Domain 2. open labelled. |
| Kammerer et al, 2018 | Domain 2. open labelled. |
| Matthew W. et al, 2018 | Domain 1. This is a pragmatic, cluster-randomized, multiple-crossover trial conducted in five intensive care units at an academic center. |

## 7.3 Trauma patients

### 7.3.1. Risk of bias assessment for studies in trauma trials

eFigure 7.5: Risk of bias assessment for the individual domains in trauma trials

### 7.3.2. Risk of bias assessment for the individual studies in trauma trials

| Author, Year | Domain 1 | Domain 2 | Domain 3 | Domain 4 | Domain 5 | Overall Bias |  |  |  |
| --- | --- | --- | --- | --- | --- | --- | --- | --- | --- |
| Shah et al, 1977 |  |  |  |  |  |  |  | Domain :   1. Randomization process 2. Deviations from intended interventions 3. Missing outcome data 4. Measurement of the outcome 5. Selection of the reported result | |
| Lowe et al, 1979 |  |  |  |  |  |  |  |  |  |
| Nagy et al, 1993 |  |  |  |  |  |  |  |  |  |
| Allison et al, 1999 |  |  |  |  |  |  |  |  |  |
| Finfer et al, 2004 |  |  |  |  |  |  |  |  |  |
| James et al, 2011 |  |  |  |  |  |  |  |  |  |
| Myburgh et al, 2012 |  |  |  |  |  |  |  |  |  |
| Young et al, 2014 |  |  |  |  |  |  |  |  | Low risk |
| Young et al, 2015 |  |  |  |  |  |  |  |  | Some concerns |
| Matthew W. et al, 2018 |  |  |  |  |  |  |  |  | High risk |

eFigure 7.6: Risk of bias assessment for trauma trials

### 7.3.3. Risk of bias notes for the individual studies in trauma trials

eTable 7.3. Risk of bias details in trauma trials

| **Study ID** | **Notes for risk of bias assessment** |
| --- | --- |
| Shah et al, 1977 | Domain 2. open labelled. |
| Lowe et al, 1979 | Domain 2. open labelled. |
| Nagy et al, 1993 | Domain 1. Allocation or randomization method was not mentioned. |
| Allison et al, 1999 | Domain 2. open labelled. |
| Matthew W. et al, 2018 | Domain 1. This is a pragmatic, cluster-randomized, multiple-crossover trial conducted in five intensive care units at an academic center. |

# Appendix 8: Results

## 8.1. Extracted outcome data in sepsis patients

### 8.1.1. Mortality in sepsis patients

| Author, Year | BC | | Saline | | Iso-Alb | | Hyper-Alb | | L-HES | | H-HES | | Gelatin | |
| --- | --- | --- | --- | --- | --- | --- | --- | --- | --- | --- | --- | --- | --- | --- |
|  | d | n | d | n | d | n | d | n | d | n | d | n | d | n |
| Rockow et al, 1983 |  |  | 4 | 11 | 5 | 7 |  |  | 3 | 7 |  |  |  |  |
| Rockow et al, 1989 |  |  |  |  | 5 | 10 |  |  |  |  | 5 | 10 |  |  |
| Asfar et al, 2000 |  |  |  |  |  |  |  |  |  |  | 10 | 16 | 12 | 18 |
| Schortgen et al, 2001 |  |  |  |  |  |  |  |  |  |  | 28 | 65 | 29 | 64 |
| Finfer et al, 2004 |  |  | 217 | 615 | 185 | 603 |  |  |  |  |  |  |  |  |
| Molnar et al, 2004 |  |  |  |  |  |  |  |  |  |  | 12 | 15 | 10 | 15 |
| Veneman et al, 2004 |  |  | 5 | 16 |  |  | 8 | 15 | 18 | 30 |  |  |  |  |
| Palumbo et al, 2006 |  |  |  |  |  |  | 3 | 10 | 4 | 10 |  |  |  |  |
| Brunkhorst et al, 2008 | 93 | 274 |  |  |  |  |  |  |  |  | 107 | 261 |  |  |
| Friedman et al, 2008 |  |  |  |  | 5 | 15 |  |  |  |  | 10 | 27 |  |  |
| Li et al, 2008 |  |  | 10 | 15 |  |  |  |  | 9 | 15 |  |  |  |  |
| McIntyre et al, 2008 |  |  | 7 | 19 |  |  |  |  |  |  | 9 | 21 |  |  |
| Dolecek et al, 2009 |  |  |  |  |  |  | 4 | 30 | 6 | 26 |  |  |  |  |
| Dubin et al, 2010 |  |  | 7 | 13 |  |  |  |  | 3 | 12 |  |  |  |  |
| Charpentier et al, 2011 |  |  | 138 | 393 |  |  | 138 | 399 |  |  |  |  |  |  |
| Siegemund, 2011 |  |  | 50 | 124 |  |  |  |  | 44 | 117 |  |  |  |  |
| Guidet et al, 2012 |  |  | 32 | 95 |  |  |  |  | 40 | 99 |  |  |  |  |
| Myburgh et al, 2012 |  |  | 224 | 945 |  |  |  |  | 248 | 976 |  |  |  |  |
| Perner et al, 2012 | 173 | 400 |  |  |  |  |  |  | 202 | 398 |  |  |  |  |
| Annane et al, 2013 | 16 | 37 | 197 | 557 |  |  | 22 | 59 | 120 | 375 |  |  | 47 | 152 |
| Caironi et al, 2014 |  |  | 389 | 893 |  |  | 365 | 888 |  |  |  |  |  |  |
| Young et al, 2015 | 7 | 35 | 9 | 42 |  |  |  |  |  |  |  |  |  |  |
| Matthew et al, 2018 | 418 | 2735 | 467 | 2646 |  |  |  |  |  |  |  |  |  |  |

Abbreviations: BC, Balanced crystalloids; Iso-Alb, Iso-oncotic albumin; Hyper-Alb, Hyperoncotic albumin; L-HES, Low molecular weight HES; H-HES, High molecular weight HES, F: Gelatin; d: no. of cases with events; n: total no. of cases

### 8.1.2. Resuscitation fluid volume in sepsis patients

| Author, Year | Treatment | Mean | Standard deviation | No. of cases |
| --- | --- | --- | --- | --- |
| Rackow et al, 1983 | Saline | 8355.5 | 1874 | 11 |
|  | Iso-oncotic albumin | 2833.3 | 266.8 | 7 |
|  | L-HES | 4568.6 | 1201.8 | 7 |
| Rackow et al, 1989 | Saline | 975 | 169 | 10 |
|  | H-HES | 900 | 205 | 10 |
| Finfer et al, 2004 (SAFE) | Saline | 1563.3 | 1536.1 | 615 |
|  | Iso-oncotic albumin | 1183.9 | 973.6 | 503 |
| Li et al, 2008 | Saline | 466.7 | 129.1 | 15 |
|  | H-HES | 100 | 338.1 | 15 |
| McIntyre et al, 2008 (FINESS) | Saline | 2100 | 600 | 19 |
|  | H-HES | 1900 | 600 | 21 |
| Dubin et al, 2010 | Saline | 8368 | 2405 | 13 |
|  | L-HES | 4682 | 1371 | 12 |
| Guidet et al, 2012 (CRYSTMAS) | Saline | 1379 | 886 | 95 |
|  | L-HES | 1709 | 1164 | 99 |
| Myburgh et al, 2012 (CHEST) | Saline | 616 | 488 | 945 |
|  | L-HES | 526 | 425 | 976 |
| Perner et al, 2012 (6S) | Balanced crystalloids | 3500 | 370.3333 | 400 |
|  | L-HES | 4000 | 308.5 | 398 |
| Annane et al, 2013 (CRISTAL) | Balanced crystalloids | 3000 | 740.6667 | 37 |
|  | Saline | 1000 | 246.8333 | 557 |
|  | Hyperoncotic albumin | 300 | 49.33333 | 59 |
|  | L-HES | 1500 | 123.3333 | 375 |
|  | Gelatin | 1500 | 246.8333 | 152 |
| Young et al, 2015 (SPLIT) | Balanced crystalloids | 2655 | 3052 | 35 |
|  | Saline | 2554 | 2120 | 42 |
| Matthew W. et al, 2018 | Balanced crystalloids | 2117 | 3157 | 2735 |
|  | Saline | 2775 | 3488 | 2646 |

Abbreviations: L-HES, Low molecular weight HES; H-HES, High molecular weight HES.

### 8.1.3. No. of acute kidney injury in sepsis patients

| Author, Year | Balanced  crystalloids | | Saline | | Iso-oncotic  albumin | | L-HES | | H-HES | | Gelatin | |
| --- | --- | --- | --- | --- | --- | --- | --- | --- | --- | --- | --- | --- |
|  | d | n | d | n | d | n | d | n | d | n | d | n |
| Schortgen et al, 2001 |  |  |  |  |  |  |  |  | 27 | 65 | 15 | 64 |
| Finfer et al, 2004 |  |  | 112 | 615 | 113 | 603 |  |  |  |  |  |  |
| Brunkhorst et al, 2008 | 62 | 272 |  |  |  |  |  |  | 91 | 261 |  |  |
| McIntyre et al, 2008 |  |  | 1 | 19 |  |  |  |  | 3 | 21 |  |  |
| Dubin et al, 2010 |  |  | 2 | 11 |  |  | 0 | 9 |  |  |  |  |
| Siegemund, 2011 |  |  | 23 | 124 |  |  | 28 | 117 |  |  |  |  |
| Guidet et al, 2012 |  |  | 11 | 96 |  |  | 21 | 100 |  |  |  |  |
| Myburgh et al, 2012 |  |  | 110 | 957 |  |  | 124 | 979 |  |  |  |  |
| Perner et al, 2012 | 127 | 400 |  |  |  |  | 148 | 398 |  |  |  |  |
| Young et al, 2015 | 7 | 35 | 9 | 42 |  |  |  |  |  |  |  |  |
| Matthew et al, 2018 | 197 | 2735 | 192 | 2646 |  |  |  |  |  |  |  |  |

Abbreviations: L-HES, Low molecular weight HES; H-HES, High molecular weight HES; d: no. of cases with events; n: total no. of cases

*Acute kidney injury including renal replacement treatment and acute renal failure

### 8.1.4. No. of renal replacement events in sepsis patients

| Author, Year | Balanced  crystalloids | | Saline | | Iso-oncotic  albumin | | L-HES | | H-HES | | Gelatin | |
| --- | --- | --- | --- | --- | --- | --- | --- | --- | --- | --- | --- | --- |
|  | d | n | d | n | d | n | d | n | d | n | d | n |
| Schortgen et al, 2001 |  |  |  |  |  |  |  |  | 27 | 65 | 15 | 64 |
| Finfer et al, 2004 |  |  | 112 | 615 | 113 | 603 |  |  |  |  |  |  |
| Brunkhorst et al, 2008 | 62 | 272 |  |  |  |  |  |  | 91 | 261 |  |  |
| McIntyre et al, 2008 |  |  | 1 | 19 |  |  |  |  | 3 | 21 |  |  |
| Dubin et al, 2010 |  |  | 2 | 11 |  |  | 0 | 9 |  |  |  |  |
| Siegemund, 2011 |  |  | 23 | 124 |  |  | 28 | 117 |  |  |  |  |
| Guidet et al, 2012 |  |  | 11 | 96 |  |  | 21 | 100 |  |  |  |  |
| Myburgh et al, 2012 |  |  | 110 | 957 |  |  | 124 | 979 |  |  |  |  |
| Perner et al, 2012 | 127 | 400 |  |  |  |  | 148 | 398 |  |  |  |  |
| Young et al, 2015 | 7 | 35 | 9 | 42 |  |  |  |  |  |  |  |  |
| Matthew et al, 2018 | 197 | 2735 | 192 | 2646 |  |  |  |  |  |  |  |  |

Abbreviations: L-HES, Low molecular weight HES; H-HES, High molecular weight HES; d: no. of cases with events; n: total no. of cases

### 8.1.5. Blood transfusion volume in sepsis patients

| Author, Year | Treatment | Mean | SD | No. of cases |
| --- | --- | --- | --- | --- |
| Rackow et al, 1983 | Saline | 291.7 | 130.6 | 11 |
|  | Iso-oncotic  albumin | 363.9 | 186 | 7 |
|  | L-HES | 757.1 | 201 | 7 |
| Finfer et al, 2004 (SAFE) | Saline | 228.3 | 227.95 | 615 |
|  | Iso-oncotic  Albumin | 307.7 | 268.7 | 603 |
| Palumbo et al, 2006 | Hyperoncotic  albumin | 280 | 130 | 10 |
|  | L-HES | 300 | 150 | 10 |
| Brunkhorst et al, 2008 (VISEP) | BC | 1000 | 1111 | 274 |
|  | H-HES | 1500 | 1481 | 261 |
| Guidet et al, 2012 (CRYSTMAS) | Saline | 165 | 354 | 95 |
|  | L-HES | 214 | 358 | 99 |
| Myburgh et al, 2012 (CHEST) | Saline | 60 | 190 | 945 |
|  | L-HES | 78 | 250 | 976 |
| Perner et al, 2012 (6S) | BC | 1055 | 266.0333 | 400 |
|  | L-HES | 1340 | 263.45 | 398 |
| Caironi et al, 2014 (ALBIOS) | Saline | 600 | 333 | 907 |
|  | Hyperoncotic  Albumin | 900 | 383 | 903 |
| Young et al, 2015 (SPLIT) | BC | 149 | 703 | 35 |
|  | Saline | 122 | 479 | 42 |
| Matthew W. et al, 2018 | BC | 7 | 113 | 2735 |
|  | Saline | 12 | 255 | 2646 |

Abbreviations: BC, Balanced crystalloids; L-HES, Low molecular weight HES; H-HES, High molecular weight HES, M: mean of blood transfusion volume; SD: standard deviation of blood transfusion volume.

### 8.1.6. No. of bleeding events requiring transfusion in sepsis patients

| Author, Year | BC | | Saline | | L-HES | | H-HES | |
| --- | --- | --- | --- | --- | --- | --- | --- | --- |
|  | d | n | d | n | d | n | d | n |
| Brunkhorst et al, 2008 | 189 | 275 |  |  |  |  | 199 | 262 |
| McIntyre et al, 2008 |  |  | 5 | 19 |  |  | 10 | 21 |
| Dubin et al, 2010 |  |  | 2 | 11 | 2 | 9 |  |  |
| Guidet, 2012 |  |  | 20 | 96 | 29 | 100 |  |  |
| Perner et al, 2012 | 204 | 380 |  |  | 243 | 376 |  |  |

Abbreviations: BC, Balanced crystalloids; L-HES, Low molecular weight HES; H-HES, High molecular weight HES; d: no. of cases with events; n: total no. of cases

### 8.1.7. Allergic events in sepsis patients

| Author, Year | BC | | Saline | | L-HES | | H-HES | |
| --- | --- | --- | --- | --- | --- | --- | --- | --- |
|  | d | n | d | n | d | n | d | n |
| Myburgh et al, 2012 |  |  | 180 | 3416 |  |  | 95 | 3358 |
| Perner et al, 2012 | 0 | 400 |  |  | 1 | 398 |  |  |

Abbreviations: BC, Balanced crystalloids; L-HES, Low molecular weight HES; H-HES, High molecular weight HES; d: no. of cases with events; n: total no. of cases

## 8.2. Extracted outcome data in surgical patients

### 8.2.1. Mortality data in surgical patients

| Author, Year | Balanced crystalloids | | Saline | | Iso-Alb | | Hyper-Alb | | L-HES | | H-HES | | Gelatin | |
| --- | --- | --- | --- | --- | --- | --- | --- | --- | --- | --- | --- | --- | --- | --- |
|  | d | n | d | n | d | n | d | n | d | n | d | n | d | n |
| Boutros et al, 1979 | 1 | 8 | 1 | 9 | 0 | 7 |  |  |  |  |  |  |  |  |
| Virgilio et al, 1979 | 1 | 14 |  |  | 1 | 15 |  |  |  |  |  |  |  |  |
| Zetterstrom et al, 1981 | 0 | 9 |  |  | 2 | 9 |  |  |  |  |  |  |  |  |
| Shires et al, 1983 | 0 | 9 |  |  | 0 | 9 |  |  |  |  |  |  |  |  |
| Prien et al, 1990 | 0 | 6 |  |  |  |  | 0 | 6 |  |  | 1 | 6 |  |  |
| Wahba et al, 1996 | 0 | 10 |  |  |  |  |  |  |  |  |  |  | 0 | 10 |
| Waters et al, 2001 | 1 | 33 | 1 | 33 |  |  |  |  |  |  |  |  |  |  |
| Linden et al, 2003 |  |  |  |  |  |  |  |  |  |  | 0 | 55 | 0 | 55 |
| Linden et al, 2005 |  |  |  |  |  |  |  |  | 0 | 64 |  |  | 1 | 68 |
| Mahmood et al. 2007 |  |  |  |  |  |  |  |  | 0 | 21 | 1 | 21 | 3 | 20 |
| Tamayo et al, 2008 | 0 | 22 |  |  |  |  |  |  |  |  |  |  | 0 | 22 |
| Soares et al, 2009 |  |  | 1 | 20 |  |  |  |  |  |  |  |  | 0 | 20 |
| Gondos et al, 2010 | 14 | 50 |  |  | 12 | 50 |  |  | 15 | 50 |  |  | 12 | 50 |
| Magder et al. 2010 |  |  | 2 | 117 |  |  |  |  |  |  | 2 | 118 |  |  |
| Hung et al, 2012 | 0 | 39 |  |  |  |  |  |  | 0 | 41 |  |  |  |  |
| Hamaji et al. 2013 | 0 | 24 |  |  |  |  |  |  | 1 | 24 |  |  |  |  |
| Yates et al, 2014 | 2 | 98 |  |  |  |  |  |  | 5 | 104 |  |  |  |  |
| Yanartas et al, 2015 |  |  |  |  |  |  |  |  |  |  |  |  |  |  |
| Young et al, 2015 | 10 | 475 | 13 | 485 |  |  |  |  |  |  |  |  |  |  |
| Kammerer et al, 2018 |  |  |  |  | 1 | 53 |  |  | 1 | 47 |  |  |  |  |
| Joosten et al, 2018 | 4 | 80 |  |  |  |  |  |  | 0 | 80 |  |  |  |  |
| Matthew W. et al, 2018 | 42 | 657 | 43 | 648 |  |  |  |  |  |  |  |  |  |  |
| Futier et al, 2020 |  |  | 64 | 386 |  |  |  |  | 88 | 389 |  |  |  |  |

Abbreviations: Iso-Alb, iso-oncotic albumin; Hyper-Alb, hyperoncotic albumin; L-HES, Low molecular weight HES; H-HES, High molecular weight HES; d: no. of case with events, n: no. of case.

### 8.2.2. Resuscitation fluid volume in surgical patients

|  | Balanced crystalloids | | | Saline | | | Iso-oncotic albumin | | | Hyperoncotic albumin | | | L-HES | | | H-HES | | | Gelatin | | |
| --- | --- | --- | --- | --- | --- | --- | --- | --- | --- | --- | --- | --- | --- | --- | --- | --- | --- | --- | --- | --- | --- |
| Author, Year | m | sd | n | m | sd | n | m | sd | n | m | sd | n | m | sd | n | m | sd | n | m | sd | n |
| Boutros et al, 1979 | 2691 | 322 | 8 | 2205 | 260 | 9 | 2066 | 102 | 7 |  |  |  |  |  |  |  |  |  |  |  |  |
| Virgilio et al, 1979 | 11300 | 800 | 14 |  |  |  | 6200 | 400 | 15 |  |  |  |  |  |  |  |  |  |  |  |  |
| Shires et al, 1983 | 8400 | 200 | 9 |  |  |  | 4800 | 300 | 9 |  |  |  |  |  |  |  |  |  |  |  |  |
| Prien et al, 1990 | 3850 | 584 | 6 |  |  |  | 463 | 49 | 6 | 463 | 49 | 6 |  |  |  | 1358 | 45 | 6 |  |  |  |
| Wahba et al, 1996 | 5882 | 812 | 10 |  |  |  |  |  |  |  |  |  |  |  |  |  |  |  | 4010 | 530 | 10 |
| Waters et al, 2001 | 6871 | 1629 | 33 | 7000 | 2592 | 33 |  |  |  |  |  |  |  |  |  |  |  |  |  |  |  |
| Linden et al, 2003 |  |  |  |  |  |  |  |  |  |  |  |  |  |  |  | 1810 | 360 | 55 | 1900 | 360 | 55 |
| Linden et al, 2005 |  |  |  |  |  |  |  |  |  |  |  |  | 4084 | 884.8 | 64 |  |  |  | 4180.8 | 865.8 | 68 |
| Mahmood et al. 2007 |  |  |  |  |  |  |  |  |  |  |  |  | 3911 | 1783 | 21 | 3443 | 1769 | 21 | 4490 | 1499 | 20 |
| Tamayo et al, 2008 | 2577.3 | 434.2 | 22 |  |  |  |  |  |  |  |  |  |  |  |  |  |  |  | 2325 | 476 | 22 |
| Soares et al, 2009 |  |  |  | 3550 | 710 | 20 |  |  |  |  |  |  |  |  |  |  |  |  | 2555 | 584 | 20 |
| Magder et al. 2010 |  |  |  | 3275 | 2270 | 117 |  |  |  |  |  |  |  |  |  | 2619 | 1988 | 118 |  |  |  |
| Hung et al, 2012 | 2303 | 1033 | 39 |  |  |  |  |  |  |  |  |  |  |  |  | 1547 | 424 | 41 |  |  |  |
| Yates et al, 2014 | 6375 | 1731 | 98 |  |  |  |  |  |  |  |  |  |  |  |  | 5398 | 1409 | 104 |  |  |  |
| Yanartas et al, 2015 | 2700 | 1074 | 66 |  |  |  |  |  |  |  |  |  | 2450 | 962 | 66 |  |  |  |  |  |  |
| Young et al, 2015 | 2655 | 3052 | 475 | 2554 | 2120 | 485 |  |  |  |  |  |  |  |  |  |  |  |  |  |  |  |
| Kammerer et al, 2018 |  |  |  |  |  |  | 10894 | 2533 | 53 |  |  |  | 11116 | 2839 | 47 |  |  |  |  |  |  |
| Joosten et al, 2018 | 12200 | 10814 | 80 |  |  |  |  |  |  |  |  |  | 7000 | 5555 | 80 |  |  |  |  |  |  |
| Matthew W. et al, 2018 | 2117 | 3157 | 657 | 2775 | 3488 | 648 |  |  |  |  |  |  |  |  |  |  |  |  |  |  |  |
| Futier et al, 2020 |  |  |  | 1250 | 360 | 386 |  |  |  |  |  |  |  |  |  |  |  |  |  |  |  |

Abbreviations: L-HES, Low molecular weight HES; H-HES, High molecular weight HES; m, mean of fluid resuscitation volume; sd: standard deviation of fluid resuscitation volume; n, sample size.

### 8.2.3. No. of acute kidney injury in surgical patients

|  | Balanced crystalloids | | Saline | | Iso-oncotic albumin | | L-HES | | H-HES | | Gelatin | |
| --- | --- | --- | --- | --- | --- | --- | --- | --- | --- | --- | --- | --- |
| Author, Year | d | n | d | n | d | n | d | n | d | n | d | n |
| Waters et al, 2001 | 4 | 33 | 5 | 33 |  |  |  |  |  |  |  |  |
| Mahmood et al. 2007 |  |  |  |  |  |  | 1 | 21 | 1 | 21 | 3 | 20 |
| Tamayo et al, 2008 | 4 | 33 | 5 | 33 |  |  |  |  |  |  |  |  |
| Soares et al, 2009 |  |  | 0 | 20 |  |  |  |  |  |  | 1 | 20 |
| Magder et al. 2010 |  |  | 18 | 117 |  |  |  |  | 19 | 118 |  |  |
| Hung et al, 2012 |  |  | 3 | 53 |  |  | 2 | 56 |  |  |  |  |
| Hamaji et al. 2013 | 0 | 24 |  |  |  |  |  |  | 1 | 24 |  |  |
| Yates et al, 2014 | 0 | 98 |  |  |  |  |  |  | 4 | 104 |  |  |
| Yanartas et al, 2015 | 5 | 66 |  |  |  |  |  |  | 7 | 66 |  |  |
| Young et al, 2015 | 26 | 470 | 24 | 478 |  |  |  |  |  |  |  |  |
| Kammerer et al, 2018 |  |  |  |  | 4 | 53 | 5 | 47 |  |  |  |  |
| Joosten et al, 2018 | 23 | 80 |  |  |  |  | 19 | 80 |  |  |  |  |
| Matthew W. et al, 2018 | 33 | 657 | 36 | 648 |  |  |  |  |  |  |  |  |
| Futier et al, 2020 |  |  | 64 | 386 |  |  | 88 | 389 |  |  |  |  |

Abbreviations: L-HES, Low molecular weight HES; H-HES, High molecular weight HES; d: no. of case with events, n: no. of case.

### 8.2.4. Blood transfusion volume in surgical patients

|  | Balanced crystalloids | | | Saline | | | Iso-oncotic  albumin | | | Hyperoncotic albumin | | | Low molecular weight  HES | | | High molecular weight  HES | | | Gelatin | | |
| --- | --- | --- | --- | --- | --- | --- | --- | --- | --- | --- | --- | --- | --- | --- | --- | --- | --- | --- | --- | --- | --- |
| Author, Year | m | sd | n | m | sd | n | m | sd | n | m | sd | n | m | sd | n | m | sd | n | m | sd | n |
| Boutros et al, 1979 | 1477 | 306 | 8 | 1850 | 370 | 9 | 1228 | 182 | 7 |  |  |  |  |  |  |  |  |  |  |  |  |
| Virgilio et al, 1979 |  |  | 14 |  |  |  |  |  |  |  |  |  |  |  |  |  |  |  |  |  |  |
| Shires et al, 1983 | 1000 | 150 | 9 |  |  |  | 1150 | 75 | 9 |  |  |  |  |  |  |  |  |  |  |  |  |
| Prien et al, 1990 | 825 | 100 | 6 |  |  |  |  |  |  | 300 | 175 | 6 |  |  |  | 450 | 175 | 6 |  |  |  |
| Waters et al, 2001 | 560 | 776 | 33 | 780 | 489 | 33 |  |  |  |  |  |  |  |  |  |  |  |  |  |  |  |
| Linden et al, 2003 |  |  |  |  |  |  |  |  |  |  |  |  |  |  |  | 490.9 | 1120.1 | 55 | 27.3 | 141.7 | 55 |
| Linden et al, 2005 |  |  |  |  |  |  |  |  |  |  |  |  | 627.5 | 1031.7 | 64 |  |  |  | 615.83 | 1117.5 | 68 |
| Mahmood et al. 2007 |  |  |  |  |  |  |  |  |  |  |  |  | 1500 | 740.74 | 21 | 1750 | 1018.52 | 21 | 1750 | 787.037 | 20 |
| Tamayo et al, 2008 | 525 | 225 | 22 |  |  |  |  |  |  |  |  |  |  |  |  |  |  |  | 485 | 225 | 22 |
| Soares et al, 2009 |  |  |  | 250 | 450 | 20 |  |  |  |  |  |  |  |  |  |  |  |  | 100 | 200 | 20 |
| Magder et al. 2010 |  |  |  | 668 | 340 | 117 |  |  |  |  |  |  |  |  |  | 711 | 449 | 118 |  |  |  |
| Hung et al, 2012 |  |  |  |  |  |  |  |  |  |  |  |  |  |  |  |  |  | 41 |  |  |  |
| Yates et al, 2014 | 565 | 211 | 98 |  |  |  |  |  |  |  |  |  |  |  |  | 582 | 383 | 104 |  |  |  |
| Yanartas et al, 2015 | 250 | 1111 | 66 |  |  |  |  |  |  |  |  |  | 250 | 1111 | 66 |  |  |  |  |  |  |
| Young et al, 2015 | 166 | 171 | 475 | 139 | 214 | 485 |  |  |  |  |  |  |  |  |  |  |  |  |  |  |  |
| Futier et al, 2020 |  |  |  | 560 | 80 | 386 |  |  |  |  |  |  | 560 | 80 | 389 |  |  |  |  |  |  |

Abbreviations: m, mean of blood transfusion volume; SD, standard deviation of blood transfusion volume; n: no. of case.

## 8.3. Extracted outcome data in trauma patients

### 8.3.1. Mortality in trauma patients

| Author, Year | Balanced crystalloids | | Saline | | Iso-oncotic  albumin | | Hyperoncotic  albumin | | Low molecular  weight HES | | High molecular  weight HES | | Gelatin | |
| --- | --- | --- | --- | --- | --- | --- | --- | --- | --- | --- | --- | --- | --- | --- |
|  | d | n | d | n | d | n | d | n | d | n | d | n | d | n |
| Shah et al, 1977 | 0 | 8 |  |  | 2 | 9 |  |  |  |  |  |  |  |  |
| Lowe et al, 1979 | 3 | 84 |  |  |  |  | 3 | 57 |  |  |  |  |  |  |
| Nagy et al, 1993 | 2 | 20 |  |  |  |  |  |  |  |  | 2 | 21 |  |  |
| Allison et al, 1999 |  |  |  |  |  |  |  |  |  |  | 1 | 30 | 0 | 29 |
| Finfer et al, 2004 |  |  | 21 | 339 | 22 | 355 |  |  |  |  |  |  |  |  |
| James et al, 2011 |  |  | 6 | 53 |  |  |  |  | 12 | 56 |  |  |  |  |
| Myburgh et al, 2012 |  |  | 18 | 263 |  |  |  |  | 18 | 258 |  |  |  |  |
| Young et al, 2014 | 7 | 32 | 8 | 33 |  |  |  |  |  |  |  |  |  |  |
| Young et al, 2015 | 7 | 40 | 8 | 61 |  |  |  |  |  |  |  |  |  |  |
| Matthew W. et al, 2018 | 131 | 1640 | 142 | 1688 |  |  |  |  |  |  |  |  |  |  |

Abbreviations: d: no. of case with events, n: no. of case.

### 8.3.2. Resuscitation fluid volume in trauma patients

|  | Balanced crystalloids | | | Saline | | | Iso-oncotic  albumin | | | Hyperoncotic  albumin | | | Low molecular  weight HES | | | High molecular  weight HES | | | Gelatin | | |
| --- | --- | --- | --- | --- | --- | --- | --- | --- | --- | --- | --- | --- | --- | --- | --- | --- | --- | --- | --- | --- | --- |
| Author, Year | m | sd | n | m | sd | n | m | sd | n | m | sd | n | m | sd | n | m | sd | n | m | sd | n |
| Shah et al, 1977 | 14600 | 400 | 8 |  |  |  | 7100 | 850 | 9 |  |  |  |  |  |  |  |  |  |  |  |  |
| Lowe et al, 1979 | 5370 | 3380 | 84 |  |  |  |  |  |  | 5870 | 3050 | 57 |  |  |  |  |  |  |  |  |  |
| Allison et al, 1999 |  |  |  |  |  |  |  |  |  |  |  |  |  |  |  | 2622 | 641 | 30 | 4955 | 1532 | 29 |
| James et al, 2011 |  |  |  | 6884 | 3259 | 53 |  |  |  |  |  |  | 5603 | 2326 | 56 |  |  |  |  |  |  |
| Young et al, 2014 | 15600 | 13300 | 22 | 14000 | 12100 | 24 |  |  |  |  |  |  |  |  |  |  |  |  |  |  |  |

Abbreviations: m, mean of blood transfusion volume; SD, standard deviation of blood transfusion volume; n: no. of case.

### 8.3.3. No. of acute kidney injury in trauma patients

|  | Balanced crystalloids | | Saline | | Hyperoncotic albumin | |
| --- | --- | --- | --- | --- | --- | --- |
| Author, Year | d | n | d | n | d | n |
| James et al, 2011 |  |  | 12 | 53 | 8 | 56 |
| Young et al, 2014 | 3 | 22 | 6 | 24 |  |  |
| Young et al, 2015 | 1 | 33 | 3 | 48 |  |  |

Abbreviations: d: no. of case with events; n: no. of case.

### 8.3.4. Blood transfusion volume in trauma patients

|  | Balanced  crystalloids | | | Saline | | | Hyperoncotic  albumin | | | Low molecular  weight HES | | | High molecular  weight HES | | | Gelatin | | |
| --- | --- | --- | --- | --- | --- | --- | --- | --- | --- | --- | --- | --- | --- | --- | --- | --- | --- | --- |
| Author, Year | m | sd | n | m | sd | n | m | sd | n | m | sd | n | m | sd | n | m | sd | n |
| Lowe et al, 1979 | 375 | 717.5 | 84 |  |  |  | 490 | 782.5 | 57 |  |  |  |  |  |  |  |  |  |
| Allison et al, 1999 |  |  |  |  |  |  |  |  |  |  |  |  | 3067 | 1856 | 30 | 2643 | 1106 | 29 |
| James et al, 2011 |  |  |  | 1634 | 1216 | 53 |  |  |  | 2248 | 1595 | 56 |  |  |  |  |  |  |
| Young et al, 2014 | 450 | 312.5 | 22 | 800 | 345 | 24 |  |  |  |  |  |  |  |  |  |  |  |  |

Abbreviations: m, mean of blood transfusion volume; SD, standard deviation of blood transfusion volume; n: no. of case.

## 8.4. Extracted outcome data in traumatic brain injury patients

### 8.4.1. Mortality in traumatic brain injury patients

|  | Balanced crystalloids | | Saline | | Iso-oncotic albumin | | Low molecular weight HES | |
| --- | --- | --- | --- | --- | --- | --- | --- | --- |
| Author, Year | d | n | d | n | d | n | d | n |
| Finfer et al, 2004 |  |  | 38 | 251 | 59 | 241 |  |  |
| Myburgh et al, 2012 |  |  | 3 | 30 |  |  | 1 | 27 |
| Young et al, 2015 | 5 | 26 | 5 | 32 |  |  |  |  |
| Matthew W. et al, 2018 | 105 | 698 | 93 | 665 |  |  |  |  |

Abbreviations: d: no. of case with events, n: no. of case.

# Appendix 9: League table and Relative ranking

## 9.1. Sepsis patients

### 9.1.1. Mortality in sepsis patients

eTable 9.1 Mortality in league table in sepsis patients

| **Balanced crystalloids** |  |  |  |  |  |  |
| --- | --- | --- | --- | --- | --- | --- |
| **0.84  (0.74,0.95)** | **Saline** |  |  |  |  |  |
| 1.00  (0.77,1.30) | 0.84 (0.66,1.06) | **Iso-oncotic**  **albumin** |  |  |  |  |
| 0.90  (0.71,1.09) | 0.93  (0.81,1.08) | 1.12  0.85,1.47) | **Hyperoncotic albumin** |  |  |  |
| **0.81 (0.69,0.95)** | 1.03  (0.91,1.18) | 0.23  (0.95,1.61) | 1.11  (0.91,1.34) | **L-HES** |  |  |
| 0.79  (0.56,1.06) | 1.06  (0.78,1.45) | 1.27 (0.87,1.84) | 1.14  (0.81,1.60) | 1.03  (0.74,1.42) | **H-HES** |  |
| 0.92  (0.66,1.29) | 0.91  (0.65,1.26) | 1.08  (0.73,1.61) | 0.97  (0.68,1.38) | 0.88  (0.63,1.23) | 0.85  (0.58, 1.25) | **Gelatin** |

Abbreviations: L-HES, Low molecular weight hydroxyethyl starch; H-HES, High molecular weight hydroxyethyl starch

*Results of the network meta-analysis are presented in the left lower half. Odd ratio and 95% confidence interval were presented and left hand side intervention was reference group. Odds ratio less than one favor the column-defining treatment.

eTable 9.2 Mortality in relative ranking probability in sepsis patients

|  | | | | | | | |
| --- | --- | --- | --- | --- | --- | --- | --- |
| Ranking\Treatment | BC | Saline | Iso-Alb | Hyper-Alb | L-HES | H-HES | Gelatin |
| Best | 31.6 | 0 | 40.3 | 5.1 | 0 | 1.8 | 21.2 |
| 2nd | 39.9 | 0.2 | 26.1 | 12.9 | 0.8 | 4.8 | 15.3 |
| 3rd | 21.7 | 3.9 | 16.2 | 25.9 | 4.7 | 8 | 19.6 |
| 4th | 6.1 | 20.7 | 10.3 | 27.6 | 10.6 | 11.5 | 13.2 |
| 5th | 0.6 | 37.4 | 3.6 | 16.6 | 21.9 | 11.1 | 8.8 |
| 6th | 0.1 | 29.7 | 2.1 | 7.9 | 33.1 | 14.6 | 12.5 |
| Worst | 0 | 8.1 | 1.4 | 4 | 28.9 | 48.2 | 9.4 |
| Mean Rank | 2.0 | 5.2 | 2.2 | 3.8 | 5.7 | 5.6 | 3.5 |
| SUCRA | 82.6 | 30.5 | 79.6 | 53.8 | 21.9 | 23.0 | 58.6 |

Abbreviations: BC, Balanced crystalloids; Iso-Alb, iso-oncotic albumin; Hyper-Alb, hyperoncotic albumin; L-HES, Low molecular weight hydroxyethyl starch; H-HES, High molecular weight hydroxyethyl starch; SUCRA, the surface under the cumulative ranking.

### 9.1.2. Resuscitation fluid volume in sepsis patients

eTable 9.3 Resuscitation fluid volume in league table in sepsis patients

| **Balanced crystalloids** |  |  |  |  |  |  |
| --- | --- | --- | --- | --- | --- | --- |
| 119.71  (-1528.21,1767.62) | **Saline** |  |  |  |  |  |
| 2075.92  (-330.42,4482.27) | **1956.22**  **(71.96,3840.47)** | **Iso-oncotic**  **albumin** |  |  |  |  |
| 1721.98  (-1102.55,4546.52) | 1602.28  (-1160.78,4365.33) | -353.94  (-3624.40,2916.52) | **Hyperoncotic albumin** |  |  |  |
| 456.83  (-1333.69,2247.35) | 337.12  (-1148.43,1822.67) | -1619.10  (-3802.63,564.44) | -1265.16  (-4057.29,1526.97) | **L-HES** |  |  |
| 1570.82  (-760.32,3901.96) | 1451.11  (-263.60,3165.82) | -505.11  (-2663.23,1653.02) | -151.17  (-3376.47,3074.14) | 1113.99  (-1089.08,3317.07) | **H-HES** |  |
| 521.98  (-2302.80,3346.76) | 402.28  (-2361.03,3165.58) | -1553.94  (-4824.61,1716.73) | -1200.00  (-4415.80,2015.80) | 65.16  (-2727.22,2857.53) | -1048.83  (-4274.35,2176.69) | **Gelatin** |

Abbreviations: L-HES, Low molecular weight hydroxyethyl starch; H-HES, High molecular weight hydroxyethyl starch

*Results of the network meta-analysis are presented in the left lower half. Mean difference and 95% confidence interval were presented and left hand side intervention was reference group. Mean difference less than zero favor the column-defining treatment.

eTable 9.4 Resuscitation fluid volume in relative ranking probability in sepsis patients

|  | | | | | | | |
| --- | --- | --- | --- | --- | --- | --- | --- |
| Ranking\Treatment | BC | Saline | Iso-Alb | Hyper-Alb | L-HES | H-HES | Gelatin |
| Best | 0.3 | 0 | 42.2 | 32.8 | 0.6 | 15 | 9.1 |
| 2nd | 1.5 | 0.3 | 32.1 | 20.8 | 4.6 | 30.4 | 10.3 |
| 3rd | 6.6 | 4.7 | 15.8 | 19.7 | 12.5 | 26.3 | 14.4 |
| 4th | 12.2 | 14.1 | 5.9 | 10.5 | 25.9 | 14.2 | 17.2 |
| 5th | 19.1 | 25.2 | 2.3 | 6.8 | 26.3 | 7.5 | 12.8 |
| 6th | 25.6 | 31 | 1.2 | 5 | 18.9 | 5 | 13.3 |
| Worst | 34.7 | 24.7 | 0.5 | 4.4 | 11.2 | 1.6 | 22.9 |
| Mean Rank | 5.6 | 5.6 | 2.1 | 5.6 | 4.8 | 2.8 | 4.6 |
| SUCRA | 23.6 | 24.1 | 81.8 | 73.0 | 36.8 | 70.6 | 40.1 |

Abbreviations: BC, Balanced crystalloids; Iso-Alb, iso-oncotic albumin; Hyper-Alb, hyperoncotic albumin; L-HES, Low molecular weight hydroxyethyl starch; H-HES, High molecular weight hydroxyethyl starch; SUCRA, the surface under the cumulative ranking.

### 9.1.3. Acute kidney injury in sepsis patients

eTable 9.5 Acute kidney injury in league table in sepsis patients

| **Balanced crystalloids** |  |  |  |  |  |
| --- | --- | --- | --- | --- | --- |
| 0.98  (0.82,1.17) | **Saline** |  |  |  |  |
| 0.95  (0.68,1.33) | 0.97  (0.72,1.29) | **Iso-oncotic**  **albumin** |  |  |  |
| **0.80**  **(0.65,0.99)** | 0.82  (0.67,1.00) | 0.85  (0.60,1.20) | **L-HES** |  |  |
| **0.54**  **(0.37,0.79)** | **0.55**  **(0.37,0.84)** | **0.57**  **(0.35,0.95)** | 0.68  (0.44,1.04) | **H-HES** |  |
| 1.26  (0.54,2.95) | 1.29  (0.54,3.05) | 1.33  (0.53,3.32) | 1.57  (0.66,3.77) | **2.32**  **(1.09,4.96)** | **Gelatin** |

Abbreviations: L-HES, Low molecular weight hydroxyethyl starch; H-HES, High molecular weight hydroxyethyl starch

*Results of the network meta-analysis are presented in the left lower half. Odd ratio and 95% confidence interval were presented and left hand side intervention was reference group. Odds ratio less than one favor the column-defining treatment. NA, not available.

eTable 9.6 Acute kidney injury in relative ranking probability in sepsis patients

|  | | | | | | |
| --- | --- | --- | --- | --- | --- | --- |
| Ranking\Treatment | BC | Saline | Iso-Alb | L-HES | H-HES | Gelatin |
| Best | 13.3 | 9.5 | 15.2 | 0 | 0 | 62 |
| 2nd | 39.6 | 28.2 | 24.5 | 1 | 0 | 6.7 |
| 3rd | 28.8 | 43.5 | 17.5 | 4.9 | 0.1 | 5.2 |
| 4th | 17.6 | 18.3 | 29.3 | 23.4 | 0.4 | 11 |
| 5th | 0.7 | 0.5 | 12.5 | 68 | 5 | 13.3 |
| Worst | 0 | 0 | 1 | 2.7 | 94.5 | 1.8 |
| Mean Rank | 2.5 | 2.7 | 3.1 | 4.6 | 5.9 | 2.0 |
| SUCRA | 69.1 | 65.2 | 57.4 | 27.0 | 1.2 | 80.1 |

Abbreviations: BC, Balanced crystalloids; Iso-Alb, iso-oncotic albumin; Hyper-Alb, hyperoncotic albumin; L-HES, Low molecular weight hydroxyethyl starch; H-HES, High molecular weight hydroxyethyl starch; SUCRA, the surface under the cumulative ranking.

### 9.1.4. Blood transfusion volume in sepsis patients

eTable 9.7 Blood transfusion volume in league table in sepsis patients

| **Balanced crystalloids** |  |  |  |  |  |
| --- | --- | --- | --- | --- | --- |
| -28.47  (-215.20,158.26) | **Saline** |  |  |  |  |
| -50.54  (-322.44,221.36) | -22.07  (-226.89,182.75) | **Iso-oncotic**  **albumin** |  |  |  |
| **-274.86**  **(-547.82,-1.89)** | **-246.38**  **(-463.94,-28.83)** | -224.31  (-516.63,68.00) | **Hyperoncotic albumin** |  |  |
| **-231.93**  **(-429.57,-34.29)** | **-203.46**  **(-349.29,-57.63)** | -181.39  (-416.57,53.79) | 42.92  (-179.10,264.94) | **L-HES** |  |
| **-500.00**  **(-857.79,-142.21)** | **-471.53**  **(-875.12,-67.94)** | **-449.46**  **(-898.85,-0.07)** | -225.14  (-675.18,224.89) | -268.07  (-676.82,140.68) | **H-HES** |

Abbreviations: L-HES, Low molecular weight hydroxyethyl starch; H-HES, High molecular weight hydroxyethyl starch

*Results of the network meta-analysis are presented in the left lower half. Mean difference and 95% confidence interval were presented and left hand side intervention was reference group. Mean difference less than zero favor the column-defining treatment.

eTable 9.8 Blood transfusion volume in relative ranking probability in sepsis patients

|  | | | | | | |
| --- | --- | --- | --- | --- | --- | --- |
| Ranking\Treatment | BC | Saline | Iso-Alb | Hyper-Alb | L-HES | H-HES |
| Best | 50.7 | 21.8 | 26.3 | 0.5 | 0 | 0.7 |
| 2nd | 24.2 | 52 | 21 | 1.5 | 0.3 | 1 |
| 3rd | 21.8 | 25 | 40.6 | 5.9 | 4.9 | 1.8 |
| 4th | 2.4 | 1.2 | 8.2 | 26.7 | 54.2 | 7.3 |
| 5th | 0.9 | 0 | 3.7 | 49.5 | 36.4 | 9.5 |
| Worst | 0 | 0 | 0.2 | 15.9 | 4.2 | 79.7 |
| Mean Rank | 1.8 | 2.1 | 2.4 | 4.7 | 4.4 | 5.6 |
| SUCRA | 84.3 | 78.9 | 71.5 | 25.8 | 32.1 | 7.4 |

Abbreviations: BC, Balanced crystalloids; Iso-Alb, iso-oncotic albumin; Hyper-Alb, hyperoncotic albumin; L-HES, Low molecular weight hydroxyethyl starch; H-HES, High molecular weight hydroxyethyl starch; SUCRA, the surface under the cumulative ranking.

## 9.2. Relative ranking probability in surgical patients

### 9.2.1. Mortality in surgical patients

eTable 9.9 Mortality in league table in surgical patients

| **Balanced crystalloids** |  |  |  |  |  |  |
| --- | --- | --- | --- | --- | --- | --- |
| 0.96  (0.67,1.38) | **Saline** |  |  |  |  |  |
| 0.99  (0.48,2.03) | 1.03  (0.47,2.22) | **Iso-oncotic**  **albumin** |  |  |  |  |
| 2.53  (0.09,74.53) | 2.62  (0.09,77.66) | 2.55  (0.08,79.74) | **Hyperoncotic albumin** |  |  |  |
| 0.74  (0.42,1.31) | 0.77  (0.42,1.40) | 0.75  (0.36,1.58) | 0.29  (0.01,8.90) | **L-HES** |  |  |
| 1.03  (0.29,3.69) | 1.06  (0.30,3.82) | 1.04  (0.25,4.23) | 0.41  (0.02,10.77) | 1.38  (0.37,5.21) | **H-HES** |  |
| 0.89  (0.43,1.82) | 0.92  (0.43,1.97) | 0.90  (0.39,2.05) | 0.35  (0.01,10.61) | 1.19  (0.57,2.49) | 0.86  (0.24,3.14) | **Gelatin** |

Abbreviations: L-HES, Low molecular weight hydroxyethyl starch; H-HES, High molecular weight hydroxyethyl starch

*Results of the network meta-analysis are presented in the left lower half. Odd ratio and 95% confidence interval were presented and left hand side intervention was reference group. Odds ratio less than one favor the column-defining treatment.

eTable 9.10 Mortality in relative ranking probability in surgical patients

|  | | | | | | | |
| --- | --- | --- | --- | --- | --- | --- | --- |
| Ranking\Treatment | BC | Saline | Iso-Alb | Hyper-Alb | L-HES | H-HES | Gelatin |
| Best | 5.4 | 4.7 | 9.7 | 63 | 1.3 | 11.5 | 4.4 |
| 2nd | 16.3 | 14.4 | 19.2 | 4.9 | 3.2 | 27.9 | 14.1 |
| 3rd | 25.6 | 22.5 | 16.9 | 2 | 5.7 | 10.7 | 16.6 |
| 4th | 25.6 | 20.9 | 15.6 | 2.7 | 11.9 | 8.2 | 15.1 |
| 5th | 17.4 | 18.6 | 16.5 | 2.9 | 18.2 | 8.7 | 17.7 |
| 6th | 7.9 | 13.2 | 14.3 | 5 | 26.9 | 13.5 | 19.2 |
| Worst | 1.8 | 5.7 | 7.8 | 19.5 | 32.8 | 19.5 | 12.9 |
| Mean Rank | 3.6 | 4.0 | 3.9 | 2.7 | 5.6 | 3.8 | 4.5 |
| SUCRA | 55.9 | 50.3 | 52.4 | 72.4 | 24.2 | 52.9 | 42.0 |

Abbreviations: BC, Balanced crystalloids; Iso-Alb, iso-oncotic albumin; Hyper-Alb, hyperoncotic albumin; L-HES, Low molecular weight hydroxyethyl starch; H-HES, High molecular weight hydroxyethyl starch; SUCRA, the surface under the cumulative ranking.

### 9.2.2. Resuscitation fluid volume in surgical patients

eTable 9.11 Resuscitation fluid volume in league table in surgical patients

| **Balanced crystalloids** |  |  |  |  |  |  |
| --- | --- | --- | --- | --- | --- | --- |
| 492.91  (-464.97,1450.79) | **Saline** |  |  |  |  |  |
| **2611.98**  **(1415.91,3808.05)** | **2119.07**  **(732.57,3505.56)** | **Iso-oncotic**  **albumin** |  |  |  |  |
| **2852.41**  **(742.41,4962.41)** | **2359.50**  **(99.73,4619.26)** | 240.43  (-2163.26,2644.12) | **Hyperoncotic albumin** |  |  |  |
| **1494.69**  **(345.02,2644.37)** | 1001.78  (-263.45,2267.01) | -1117.29  (-2572.46,337.88) | -1357.72  (-3694.89,979.46) | **L-HES** |  |  |
| **1462.11**  **(418.29,2505.93)** | 969.19  (-256.26,2194.64) | -1149.87  (-2672.91,373.16) | -1390.31  (-3490.12,709.51) | -32.59  (-1381.81,1316.64) | **H-HES** |  |
| **1154.11**  **(67.30,2240.92)** | 661.20  (-566.51,1888.91) | -1457.87  (-2983.58,67.85) | -1698.30  (-3982.37,585.77) | -340.58  (-1592.55,911.39) | -307.99  (-1521.13,905.14) | **Gelatin** |

Abbreviations: L-HES, Low molecular weight hydroxyethyl starch; H-HES, High molecular weight hydroxyethyl starch

*Results of the network meta-analysis are presented in the left lower half. Mean difference and 95% confidence interval were presented and left hand side intervention was reference group. Mean difference less than zero favor the column-defining treatment.

eTable 9.12 Resuscitation fluid volume in relative ranking probability in surgical patients

|  | | | | | | | |
| --- | --- | --- | --- | --- | --- | --- | --- |
| Ranking\Treatment | BC | Saline | Iso-Alb | Hyper-Alb | L-HES | H-HES | Gelatin |
| Best | 0 | 0 | 40.7 | 56.8 | 1.3 | 0.7 | 0.5 |
| 2nd | 0 | 0.1 | 51.1 | 26.7 | 11.9 | 7.1 | 3.1 |
| 3rd | 0 | 1.3 | 5.6 | 7.9 | 33.4 | 36 | 15.8 |
| 4th | 0.1 | 4.9 | 1.7 | 4.1 | 29.3 | 29.8 | 30.1 |
| 5th | 1.2 | 16.1 | 0.9 | 2.3 | 19.5 | 22.1 | 37.9 |
| 6th | 16.5 | 62.6 | 0 | 1.5 | 4.1 | 4.2 | 11.1 |
| Worst | 82.2 | 15 | 0 | 0.7 | 0.5 | 0.1 | 1.5 |
| Mean Rank | 6.8 | 5.9 | 1.8 | 1.7 | 3.7 | 3.7 | 4.5 |
| SUCRA | 3.5 | 18.6 | 87.4 | 88.2 | 55.0 | 55.2 | 42.0 |

Abbreviations: BC, Balanced crystalloids; Iso-Alb, iso-oncotic albumin; Hyper-Alb, hyperoncotic albumin; L-HES, Low molecular weight hydroxyethyl starch; H-HES, High molecular weight hydroxyethyl starch; SUCRA, the surface under the cumulative ranking

### 9.2.3. Acute kidney injury in surgical patients

eTable 9.13 Acute kidney injury in league table in surgical patients

| **Balanced**  **crystalloids** |  |  |  |  |  |
| --- | --- | --- | --- | --- | --- |
| 1.02  (0.73,1.40) | **Saline** |  |  |  |  |
| 1.19  (0.27,5.15) | 1.17  (0.27,5.04) | **Iso-oncotic**  **albumin** |  |  |  |
| 0.81  (0.50,1.32) | 0.80  (0.50,1.28) | 0.69  (0.17,2.74) | **L-HES** |  |  |
| 0.79  (0.42,1.49) | 0.78  (0.43,1.41) | 0.66  (0.14,3.24) | 0.97  (0.45,2.09) | **H-HES** |  |
| 0.25  (0.05,1.31) | 0.24  (0.05,1.27) | 0.21  (0.02,1.83) | 0.30  (0.06,1.62) | 0.31  (0.06,1.69) | **Gelatin** |

Abbreviations: L-HES, Low molecular weight hydroxyethyl starch; H-HES, High molecular weight hydroxyethyl starch

*Results of the network meta-analysis are presented in the left lower half. Odd ratio and 95% confidence interval were presented and left hand side intervention was reference group. Odds ratio less than one favor the column-defining treatment.

eTable 9.14 Acute kidney injury in relative ranking probability in surgical patients

|  | | | | | | |
| --- | --- | --- | --- | --- | --- | --- |
| Ranking\Treatment | BC | Saline | Iso-Alb | L-HES | H-HES | Gelatin |
| Best | 17.3 | 15.4 | 53.7 | 2.7 | 8 | 2.9 |
| 2nd | 29.4 | 39.3 | 6.3 | 11.6 | 10.6 | 2.8 |
| 3rd | 31 | 27.7 | 7.1 | 18.2 | 14.1 | 1.9 |
| 4th | 17.7 | 13.9 | 11.2 | 34.5 | 21.2 | 1.5 |
| 5th | 4.3 | 3.4 | 16.3 | 29.5 | 39.1 | 7.4 |
| Worst | 0.3 | 0.3 | 5.4 | 3.5 | 7 | 83.5 |
| Mean Rank | 2.6 | 2.5 | 2.5 | 3.9 | 3.9 | 5.7 |
| SUCRA | 67.9 | 70.3 | 70.0 | 42.8 | 42.0 | 7.0 |

Abbreviations: BC, Balanced crystalloids; Iso-Alb, iso-oncotic albumin; Hyper-Alb, hyperoncotic albumin; L-HES, Low molecular weight hydroxyethyl starch; H-HES, High molecular weight hydroxyethyl starch; SUCRA, the surface under the cumulative ranking.

### 9.2.4. Blood transfusion volume in surgical patients

eTable 9.15 Blood transfusion volume in league table in sepsis patients

| **Balanced crystalloids** |  |  |  |  |  |  |
| --- | --- | --- | --- | --- | --- | --- |
| -90.48  (-312.62,131.66) | **Saline** |  |  |  |  |  |
| 127.45  (-188.51,443.41) | 217.93  (-137.08,572.95) | **Iso-oncotic**  **albumin** |  |  |  |  |
| 338.28  (-87.04,763.59) | 428.76  (-31.33,888.84) | 210.82  (-315.75,737.39) | **Hyperoncotic albumin** |  |  |  |
| 49.49  (-254.71,353.69) | 139.97 (  -157.31,437.26) | -77.96  (-500.18,344.26) | -288.78  (-793.47,215.91) | **L-HES** |  |  |
| -22.92  (-267.03,221.19) | 67.56  (-198.10,333.21) | -150.37  (-538.98,238.23) | -361.20  (-790.26,67.87) | -72.42  (-406.32,261.49) | **H-HES** |  |
| 97.32  (-174.62,369.26) | 187.80  (-89.79,465.39) | -30.13  (-433.76,373.49) | -240.96  (-721.72,239.80) | 47.82  (-261.96,357.61) | 120.24  (-168.79,409.27) | **Gelatin** |

Abbreviations: L-HES, Low molecular weight hydroxyethyl starch; H-HES, High molecular weight hydroxyethyl starch

*Results of the network meta-analysis are presented in the left lower half. Mean difference and 95% confidence interval were presented and left hand side intervention was reference group. Mean difference less than zero favor the column-defining treatment.

eTable 9.16 Blood transfusion volume in relative ranking probability in surgical patients

|  | | | | | | | |
| --- | --- | --- | --- | --- | --- | --- | --- |
| Ranking\Treatment | BC | Saline | Iso-Alb | Hyper-Alb | L-HES | H-HES | Gelatin |
| Best | 0.7 | 0.1 | 16 | 67.2 | 6.4 | 0.9 | 8.7 |
| 2nd | 3.3 | 0.6 | 32.7 | 16.8 | 16.8 | 4.4 | 25.4 |
| 3rd | 15.1 | 2.8 | 16.3 | 6.1 | 18.7 | 12.3 | 28.7 |
| 4th | 23.8 | 7.3 | 12 | 3.1 | 18.3 | 17.5 | 18 |
| 5th | 28.4 | 13.9 | 9.2 | 2 | 15.1 | 20.8 | 10.6 |
| 6th | 20.2 | 26.5 | 6.7 | 2.1 | 16.4 | 22 | 6.1 |
| Worst | 8.5 | 48.8 | 7.1 | 2.7 | 8.3 | 22.1 | 2.5 |
| Mean Rank | 4.8 | 6.1 | 3.2 | 1.6 | 4.0 | 5.0 | 3.3 |
| SUCRA | 37.3 | 15.4 | 62.6 | 89.4 | 50.7 | 33.1 | 61.5 |

Abbreviations: BC, Balanced crystalloids; Iso-Alb, iso-oncotic albumin; Hyper-Alb, hyperoncotic albumin; L-HES, Low molecular weight hydroxyethyl starch; H-HES, High molecular weight hydroxyethyl starch; SUCRA, the surface under the cumulative ranking.

## 9.3. Relative ranking probability in trauma patients

### 9.3.1. Mortality in trauma patients

eTable 9.17 Mortality in league table in trauma patients

| **Balanced crystalloids** |  |  |  |  |  |  |
| --- | --- | --- | --- | --- | --- | --- |
| 0.95  (0.75,1.20) | **Saline** |  |  |  |  |  |
| 0.89  (0.47,1.71) | 0.94  (0.51,1.72) | **Iso-oncotic**  **albumin** |  |  |  |  |
| 0.67  (0.13,3.43) | 0.70  (0.13,3.66) | 0.75  (0.13,4.33) | **Hyperoncotic albumin** |  |  |  |
| 0.75  (0.41,1.40) | 0.79  (0.45,1.40) | 0.84  (0.37,1.94) | 1.13  (0.20,6.49) | **L-HES** |  |  |
| 1.06  (0.13,8.31) | 1.11  (0.14,8.86) | 1.18  (0.14,10.26) | 1.58  (0.11,22.05) | 1.40  (0.16,12.09) | **H-HES** |  |
| 3.17  (0.07,147.62) | 3.33  (0.07,156.37) | 3.54  (0.07,174.22) | 4.75  (0.07,309.30) | 4.21  (0.09,206.07) | 3.00  (0.12,76.68) | **Gelatin** |

Abbreviations: L-HES, Low molecular weight hydroxyethyl starch; H-HES, High molecular weight hydroxyethyl starch

*Results of the network meta-analysis are presented in the left lower half. Odd ratio and 95% confidence interval were presented and left hand side intervention was reference group. Odds ratio less than one favor the column-defining treatment.

eTable 9.18 Mortality in relative ranking probability in trauma patients

|  | | | | | | | |
| --- | --- | --- | --- | --- | --- | --- | --- |
| Ranking\Treatment | BC | Saline | Iso-Alb | Hyper-Alb | L-HES | H-HES | Gelatin |
| Best | 4.8 | 3 | 6.1 | 10.2 | 2 | 10.7 | 63.2 |
| 2nd | 19.9 | 10.5 | 13.6 | 11.5 | 4.5 | 32.1 | 7.9 |
| 3rd | 27.8 | 24 | 17.3 | 9.1 | 9.5 | 9.2 | 3.1 |
| 4th | 27.5 | 29.7 | 16.1 | 5.2 | 15.7 | 4.3 | 1.5 |
| 5th | 14.6 | 23.2 | 19.4 | 10.7 | 19.5 | 8 | 4.6 |
| 6th | 4.7 | 8.5 | 18.5 | 17.2 | 27.8 | 15.9 | 7.4 |
| Worst | 0.7 | 1.1 | 9 | 36.1 | 21 | 19.8 | 12.3 |
| Mean Rank | 3.4 | 3.9 | 4.3 | 4.9 | 5.2 | 3.8 | 2.5 |
| SUCRA | 60.0 | 51.5 | 44.9 | 34.6 | 30.4 | 53.2 | 75.3 |

Abbreviations: BC, Balanced crystalloids; Iso-Alb, iso-oncotic albumin; Hyper-Alb, hyperoncotic albumin; L-HES, Low molecular weight hydroxyethyl starch; H-HES, High molecular weight hydroxyethyl starch; SUCRA, the surface under the cumulative ranking.

### 9.3.2. Fluid resuscitation volume in trauma patients

eTable 9.18 Fluid resuscitation volume in league table in trauma patients

| **Balanced crystalloids** |  |  |  |  |
| --- | --- | --- | --- | --- |
| 1595  (-5767,8959.46) | **Saline** |  |  |  |
| **7500**  **(6879,8120)** | 5904  (-1485,13293) | **Iso-oncotic**  **albumin** |  |  |
| -500  (-1572,572) | -2095  (-9537,5345) | **-8000**  **(-9238,-6761)** | **Hyperoncotic albumin** |  |
| 2876  (-4563,10317) | **1280**  **(212,2349**) | -4623  (-12089,2843) | 3376  (-4140,10894) | **L-HES** |

Abbreviations: L-HES, Low molecular weight hydroxyethyl starch.

*Results of the network meta-analysis are presented in the left lower half. Odd ratio and 95% confidence interval were presented and left hand side intervention was reference group. Odds ratio less than one favor the column-defining treatment.

eTable 9.19 Fluid resuscitation volume in relative ranking probability in trauma patients

|  | | | | | |
| --- | --- | --- | --- | --- | --- |
| Ranking\Treatment | BC | Saline | Iso-Alb | Hyper-Alb | L-HES |
| Best | 0 | 0 | 88.3 | 0 | 11.7 |
| 2nd | 19.6 | 6.7 | 5.5 | 5.1 | 63.1 |
| 3rd | 13.4 | 58 | 6.2 | 17 | 5.4 |
| 4th | 53 | 5.6 | 0 | 21.9 | 19.5 |
| Worst | 14 | 29.7 | 0 | 56 | 0.3 |
| Mean Rank | 3.6 | 3.5 | 1.2 | 4.4 | 2.3 |
| SUCRA | 34.0 | 36.9 | 95.2 | 15.8 | 68.1 |

Abbreviations: BC, Balanced crystalloids; Iso-Alb, iso-oncotic albumin; Hyper-Alb, hyperoncotic albumin; L-HES, Low molecular weight hydroxyethyl starch; H-HES, High molecular weight hydroxyethyl starch; SUCRA, the surface under the cumulative ranking.

### 9.3.3. Adverse renal events in trauma patients

eTable 9.20 Adverse renal events in league table in trauma patients

| **Balanced crystalloids** |  |  |
| --- | --- | --- |
| 0.56  (0.21,1.53) | **Saline** |  |
| 0.83  (0.13,1.69) | 2.12  (0.59, 7.57) | **Hyperoncotic albumin** |

Abbreviations: L-HES, Low molecular weight hydroxyethyl starch.

*Results of the network meta-analysis are presented in the left lower half. Odd ratio and 95% confidence interval were presented and left hand side intervention was reference group. Odds ratio less than one favor the column-defining treatment.

eTable 9.21 Adverse renal events in relative ranking probability in trauma patients

|  | | | |
| --- | --- | --- | --- |
| Ranking\Treatment | BC | Saline | Hyper-Alb |
| Best | 60.2 | 1.7 | 38.1 |
| 2nd | 29.2 | 22.2 | 48.6 |
| Worst | 10.6 | 76.1 | 13.3 |
| Mean Rank | 1.6 | 2.7 | 1.7 |
| SUCRA | 71.3 | 15.2 | 63.5 |

Abbreviations: BC, Balanced crystalloids; Iso-Alb, iso-oncotic albumin; Hyper-Alb, hyperoncotic albumin; L-HES, Low molecular weight hydroxyethyl starch; H-HES, High molecular weight hydroxyethyl starch; SUCRA, the surface under the cumulative ranking.

### 9.3.4. Blood transfusion volume in trauma patients

eTable 9.21 Blood transfusion volume in league table in trauma patients

| **Balanced crystalloids** |  |  |  |
| --- | --- | --- | --- |
| **-350.00**  **(-540.00,-159.99)** | **Saline** |  |  |
| -115.00  (-369.58,139.58) | 235.00  (-82.67,552.66) | **Hyperoncotic albumin** |  |
| **-963.99**  **(-1527.71,-400.26)** | **-613.99**  **(-1144.73,-83.25)** | **-848.99**  **(-1467.53,-230.44)** | **L-HES** |

Abbreviations: L-HES, Low molecular weight hydroxyethyl starch.

*Results of the network meta-analysis are presented in the left lower half. Odd ratio and 95% confidence interval were presented and left hand side intervention was reference group. Odds ratio less than one favor the column-defining treatment.

eTable 9.22 Blood transfusion volume in relative ranking probability in trauma patients

|  | | | | |
| --- | --- | --- | --- | --- |
| Ranking\Treatment | BC | Saline | Hyper-Alb | L-HES |
| Best | 83.1 | 0 | 16.9 | 0 |
| 2nd | 16.9 | 8.4 | 74.5 | 0.2 |
| 3rd | 0 | 90.3 | 8.5 | 1.2 |
| Worst | 0 | 1.3 | 0.1 | 98.6 |
| Mean Rank | 1.2 | 2.9 | 1.9 | 4.0 |
| SUCRA | 93.4 | 35.5 | 70.6 | 0.5 |

Abbreviations: BC, Balanced crystalloids; Iso-Alb, iso-oncotic albumin; Hyper-Alb, hyperoncotic albumin; L-HES, Low molecular weight hydroxyethyl starch; H-HES, High molecular weight hydroxyethyl starch; SUCRA, the surface under the cumulative ranking.

### 9.3.5. Mortality in traumatic brain injury patients

eTable 9.23 Mortality in league table in traumatic brain injury patients

| **Balanced**  **crystalloids** | 1.10  (0.82,1.47) | NA | NA |
| --- | --- | --- | --- |
| 1.29 (0.33,5.03) | **Saline** | **0.55**  **(0.35,0.86)** | 2.86  (0.28,33.33) |
| 0.71 (0.17,2.98) | **0.55 (0.35,0.87)** | **Iso-oncotic**  **albumin** | NA |
| 3.71 (0.25,55.10) | 2.89 (0.28,29.58) | 5.25 (0.49,56.16) | **L-HES** |

Abbreviations: L-HES, Low molecular weight hydroxyethyl starch

*Results of the network meta-analysis are presented in the left lower half. Odd ratio and 95% confidence interval were presented and left hand side intervention was reference group. Odds ratio less than one favor the column-defining treatment.

eTable 9.24 Mortality in relative ranking probability in traumatic brain injury patients

|  | | | | |
| --- | --- | --- | --- | --- |
| Ranking\Treatment | Balanced  crystalloids | Saline | Iso-oncotic  albumin | L-HES |
| Best | 14.4 | 11.5 | 0 | 74.1 |
| 2nd | 27.3 | 58.9 | 3.9 | 9.9 |
| 3rd | 29.6 | 29.5 | 33 | 7.9 |
| Worst | 28.7 | 0.1 | 63.1 | 8.1 |
| Mean Rank | 2.8 | 2.2 | 3.6 | 1.4 |
| SUCRA | 40.0 | 60.6 | 13.4 | 86.1 |

Abbreviations: L-HES, Low molecular weight hydroxyethyl starch; SUCRA, the surface under the cumulative ranking.

## 9.4. Interval plot for surgical and trauma patients


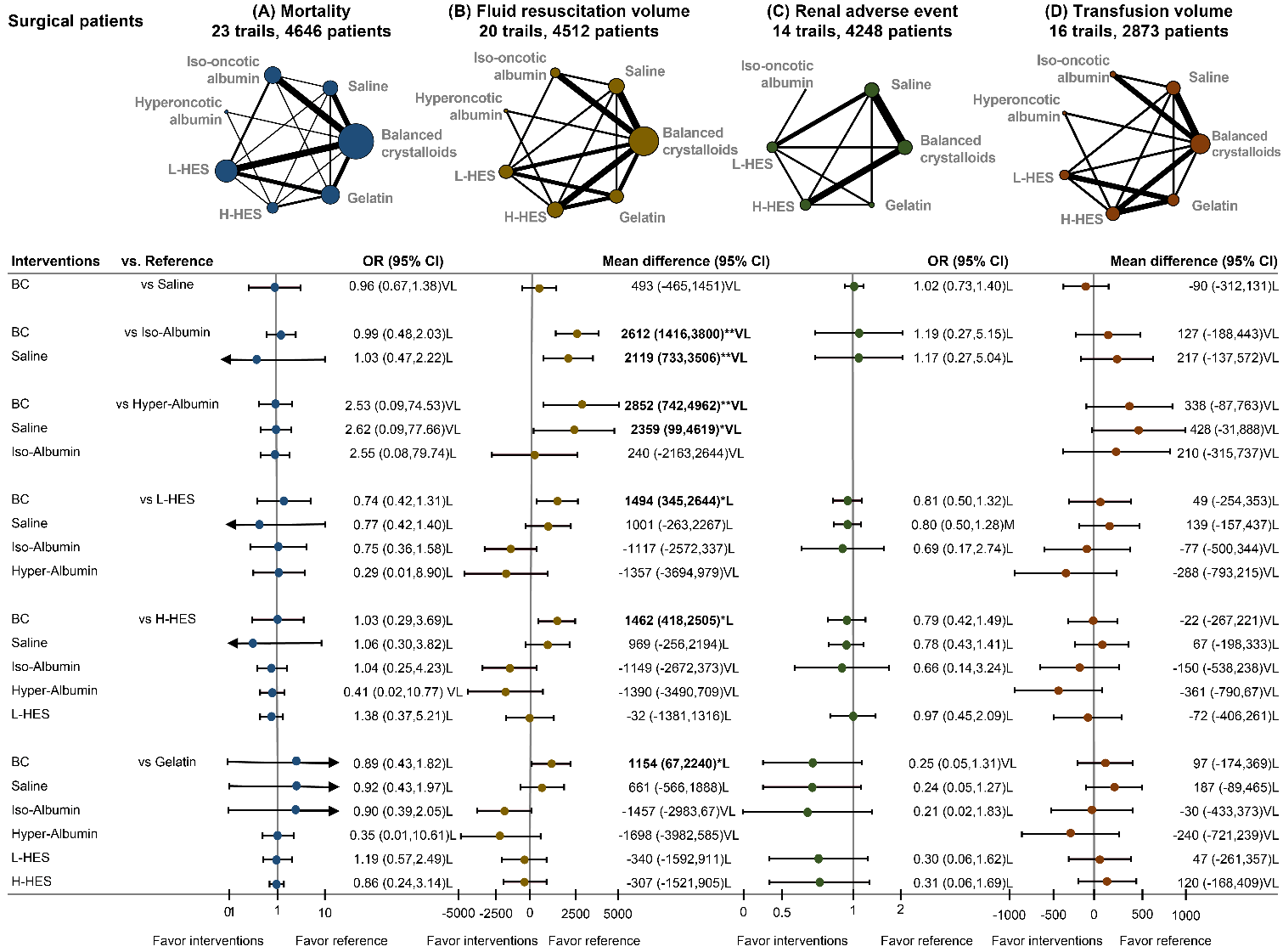


eFigure.9.1. Network geometry and forest plot in surgical patients

(A) Mortality, (B) Fluid resuscitation amount, (C) Acute kidney injury, (D) Transfusion amount. (OR, odds ration; *, p<0.05; **, p<0.01; H, high confidence rating; M, moderate confident rating; L, low confidence rating；VL, very low confidence rating; BC, balanced crystalloids; Iso-albumin, iso-oncotic albumin; Hyper-albumin, hyperoncotic albumin; L-HES, low molecular weight hydroxyethyl starch; H-HES, high molecular weight hydroxyethyl starch)


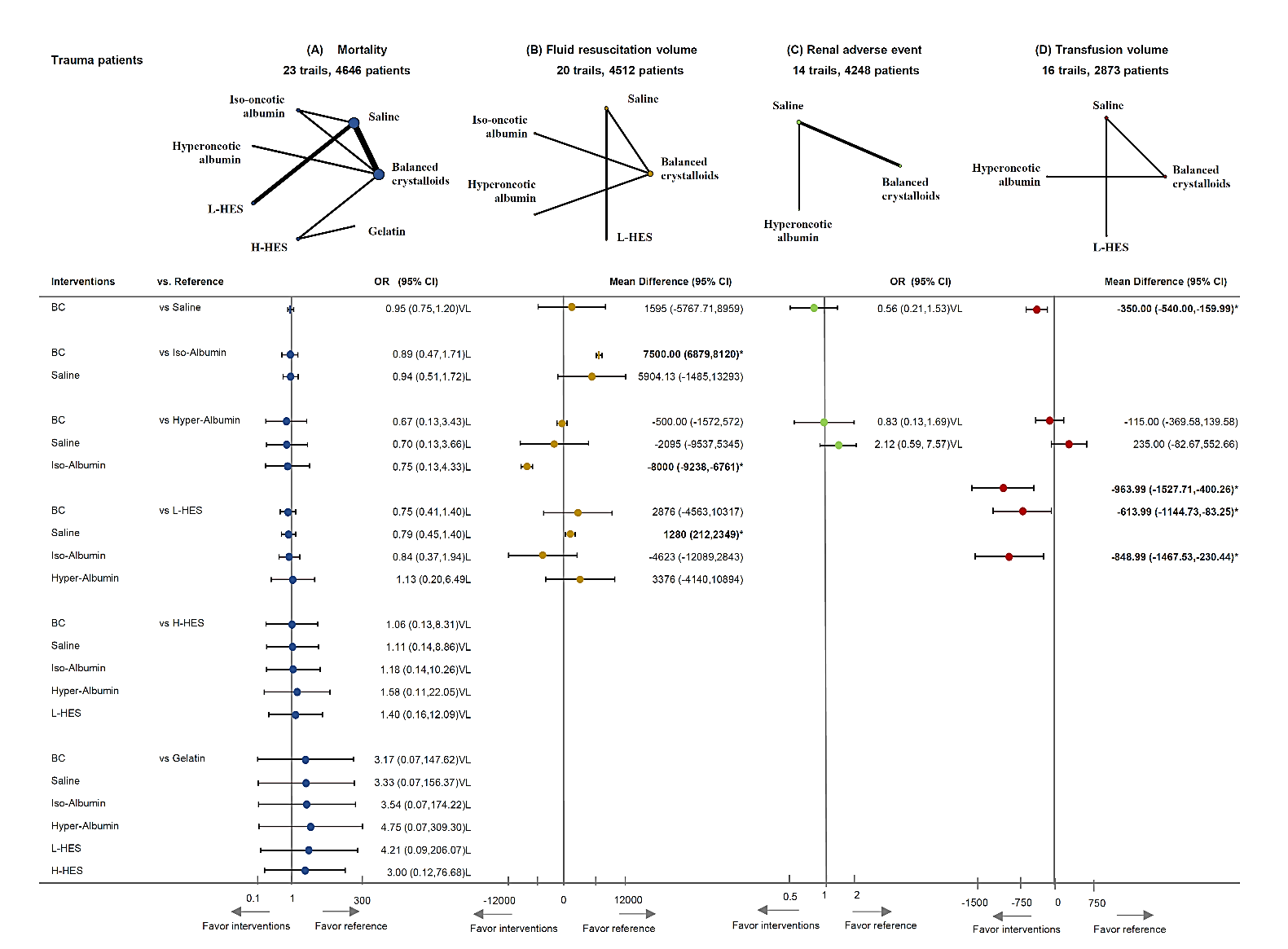


eFigure 9.2. Network geometry and forest plot in trauma patients

(A) Mortality, (B) Fluid resuscitation amount, (C) Acute kidney injury, (D) Transfusion amount. (OR, odds ration; *, p<0.05; **, p<0.01; L, low confidence rating；VL, very low confidence rating; BC, balanced crystalloids; Iso-albumin, iso-oncotic albumin; Hyper-albumin, hyperoncotic albumin; L-HES, low molecular weight hydroxyethyl starch; H-HES, high molecular weight hydroxyethyl starch)


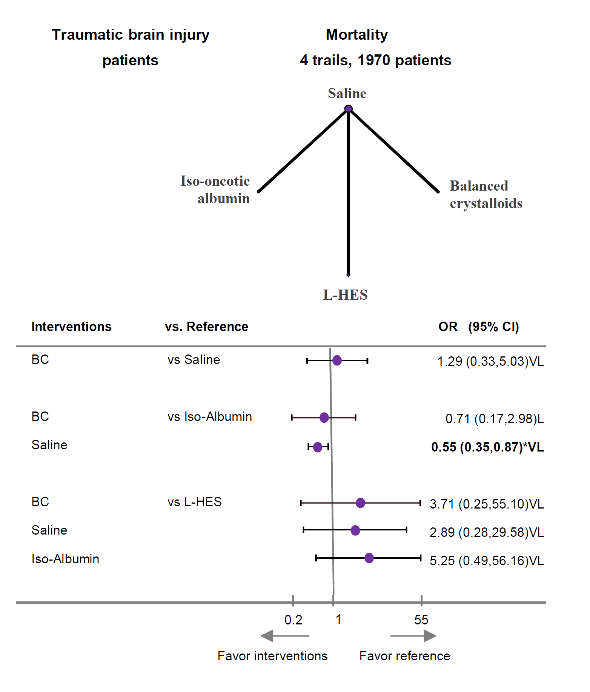


eFigure 9.3. Network geometry and forest plot in traumatic brain injury patients

(OR, odds ration; *, p<0.05; **, p<0.01; L, low confidence rating；VL, very low confidence rating; BC, balanced crystalloids; Iso-albumin, iso-oncotic albumin)

# Appendix 10: Publication bias

## 10.1. Publication bias in sepsis patients

### 10.1.1 Mortality in sepsis patients


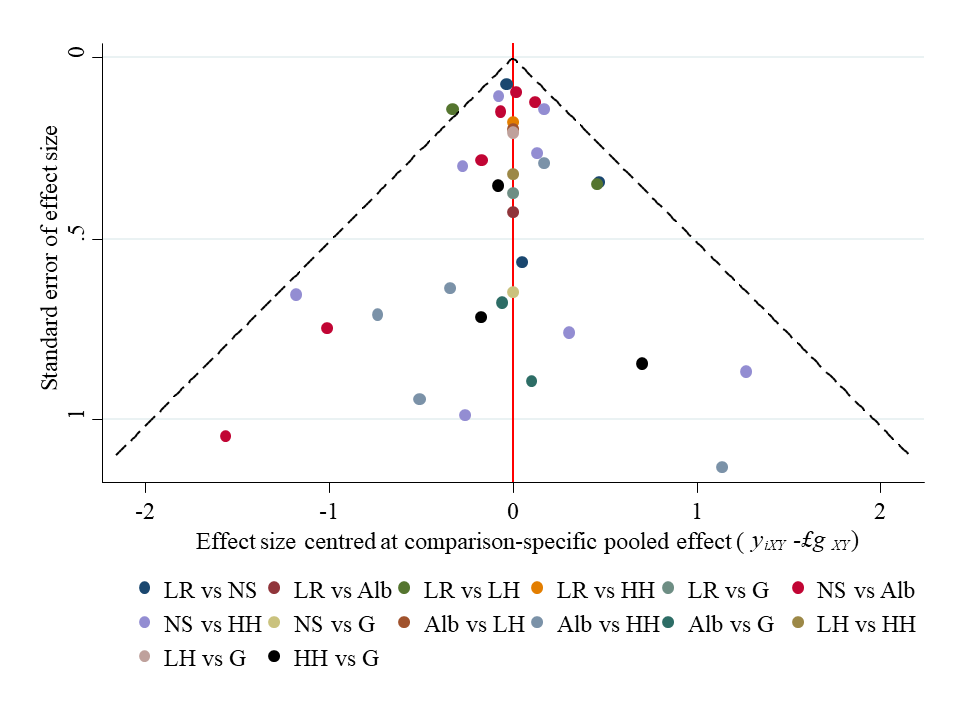


*BC, Balanced crystalloids; Alb. albumin, LH, L-HES, HH, H-HES, G, gelatin.

| Egger's test for small-study effects: | | | | | | |
| --- | --- | --- | --- | --- | --- | --- |
| Std_Eff | Coef. | Std. Err. | t | P>t | [95% Conf. | Interval] |
| slope | -0.00769 | 0.048601 | -0.16 | 0.875 | -0.10646 | 0.0910759 |
| bias | -0.05582 | 0.228931 | -0.24 | 0.809 | -0.52106 | 0.4094294 |
| Test of H0: no small-study effects= P = 0.809 | | | | | | |

### 10.1.2. Resuscitation fluid volume in sepsis patients


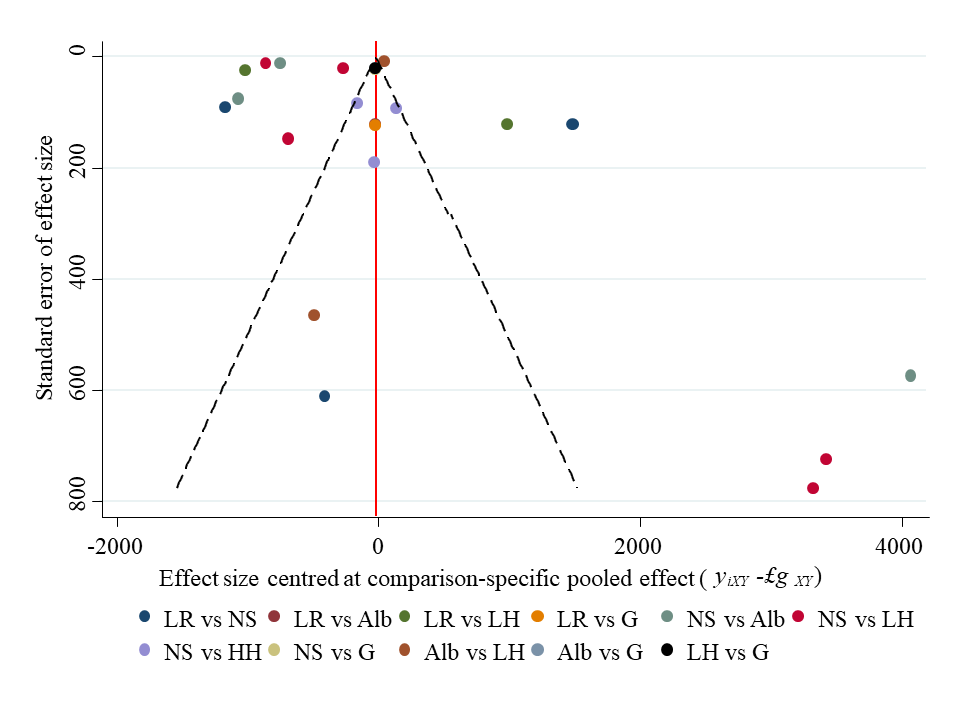


*BC, Balanced crystalloids; Alb. albumin, LH, L-HES, HH, H-HES, G, gelatin.

| Egger's test for small-study effects: | | | | | | |
| --- | --- | --- | --- | --- | --- | --- |
| Std_Eff | Coef. | Std. Err. | t | P>t | [95% Conf. | Interval] |
| slope | -361.821 | 124.0254 | -2.92 | 0.008 | -619.746 | -103.896 |
| bias | 2.059632 | 4.969831 | 0.41 | 0.683 | -8.2757 | 12.39496 |
| Test of H0: no small-study effects P = 0.683 | | | | | | |

### 10.1.3. Acute kidney injury in sepsis patients

**
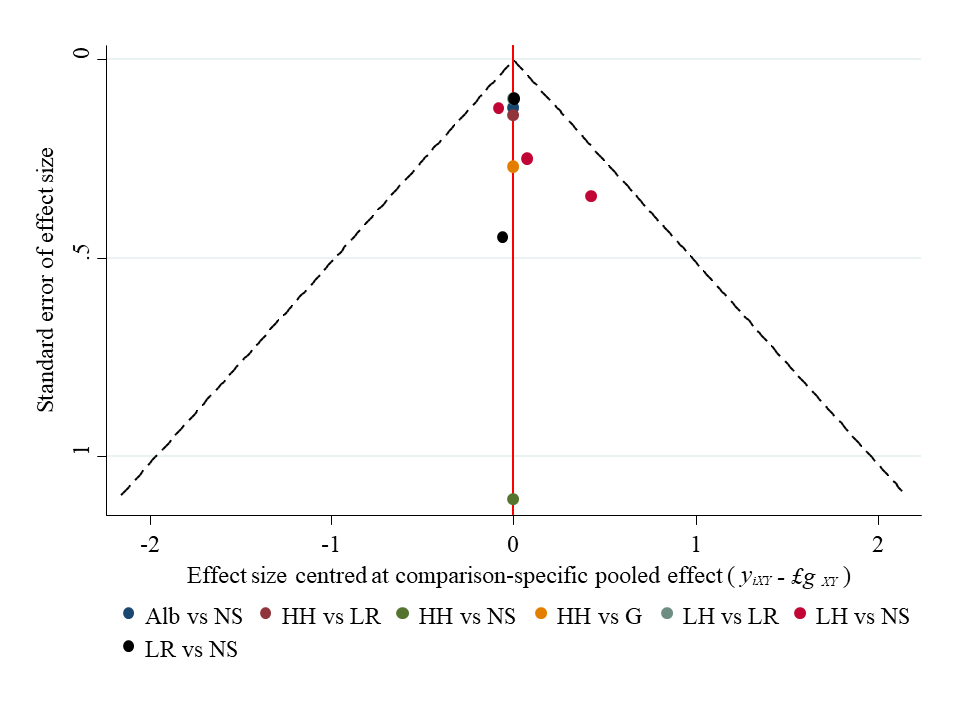
**

*BC, Balanced crystalloids; Alb. albumin, LH, L-HES, HH, H-HES, G, gelatin.

.

| Egger's test for small-study effects: | | | | | | |
| --- | --- | --- | --- | --- | --- | --- |
| Std_Eff | Coef. | Std. Err. | t | P>t | [95% Conf. | Interval] |
| slope | 0.013565 | 0.067042 | 0.2 | 0.844 | -0.13809 | 0.165224 |
| bias | -0.03631 | 0.341617 | -0.11 | 0.918 | -0.8091 | 0.736483 |
| Test of H0: no small-study effects P = 0.918 | | | | | | |

### 10.1.4. Blood transfusion volume in sepsis patients

**
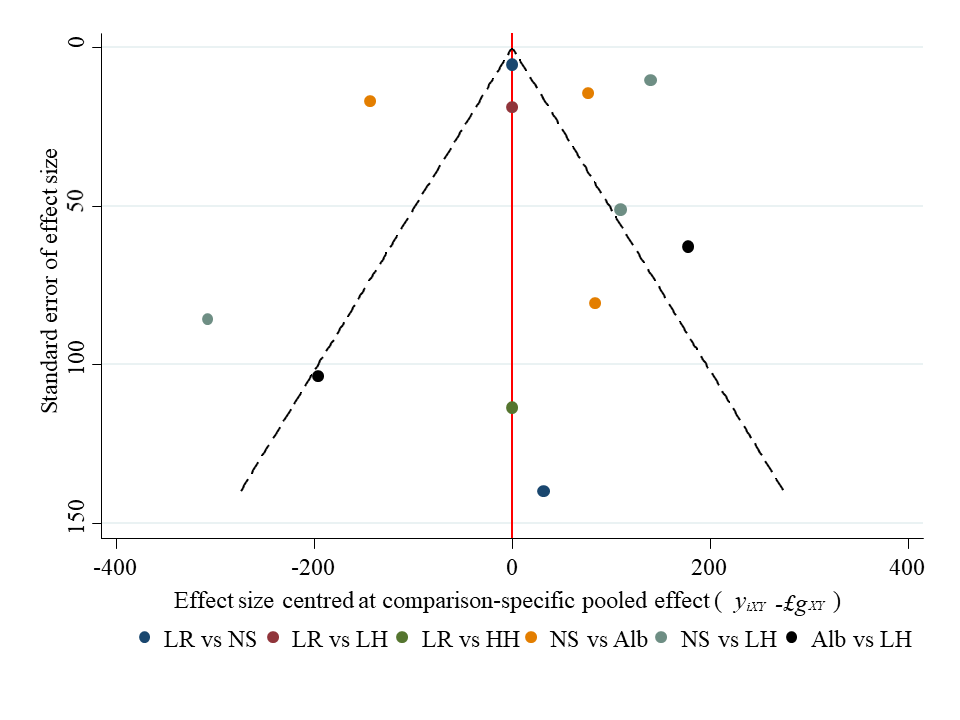
**

*BC, Balanced crystalloids; Alb. albumin, LH, L-HES, HH, H-HES, G, gelatin.

| Egger's test for small-study effects: | | | | | | |
| --- | --- | --- | --- | --- | --- | --- |
| Std_Eff | Coef. | Std. Err. | t | P>t | [95% Conf. | Interval] |
| slope | 24.32321 | 30.79318 | 0.79 | 0.448 | -44.2883 | 92.93468 |
| bias | -0.16245 | 2.110716 | -0.08 | 0.94 | -4.86542 | 4.54052 |
| Test of H0: no small-study effects P = 0.940 | | | | | | |

## 10.2. Publication bias in surgical patients

### 10.2.1. Mortality in surgical patients


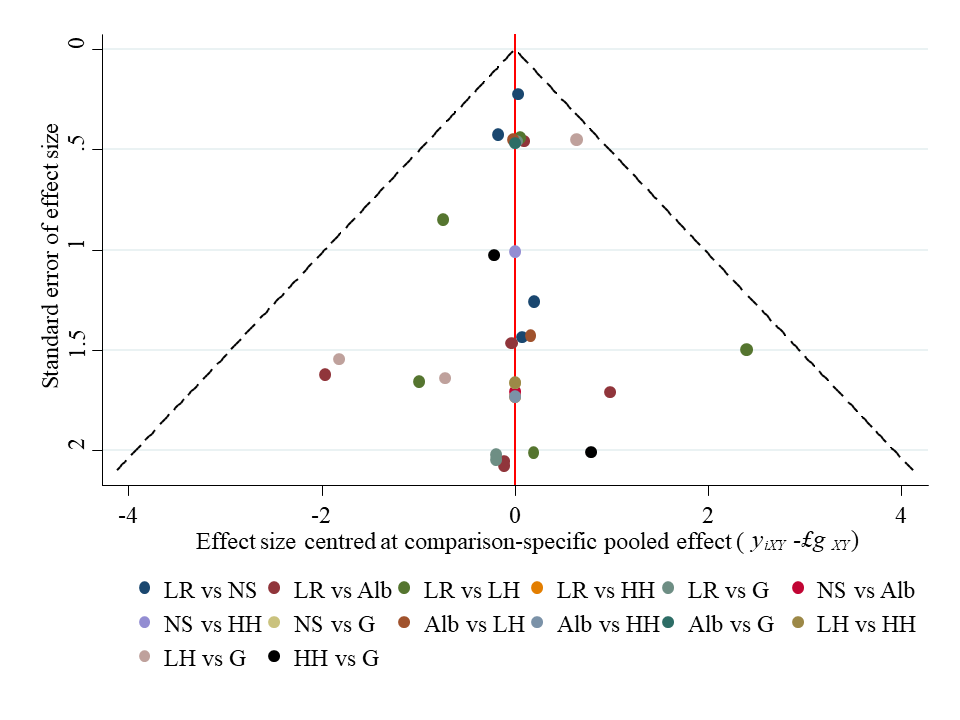


*BC, Balanced crystalloids; Alb. albumin, LH, L-HES, HH, H-HES, G, gelatin.

| Egger's test for small-study effects: | | | | | | |
| --- | --- | --- | --- | --- | --- | --- |
| Std_Eff | Coef. | Std. Err. | t | P>t | [95% Conf. | Interval] |
| slope | 0.093672 | 0.110141 | 0.85 | 0.402 | -0.13127 | 0.318609 |
| bias | -0.11257 | 0.157346 | -0.72 | 0.48 | -0.43392 | 0.208768 |
| Test of H0: no small-study effects P = 0.480 | | | | | | |

### 10.2.2. Resuscitation fluid volume in surgical patients


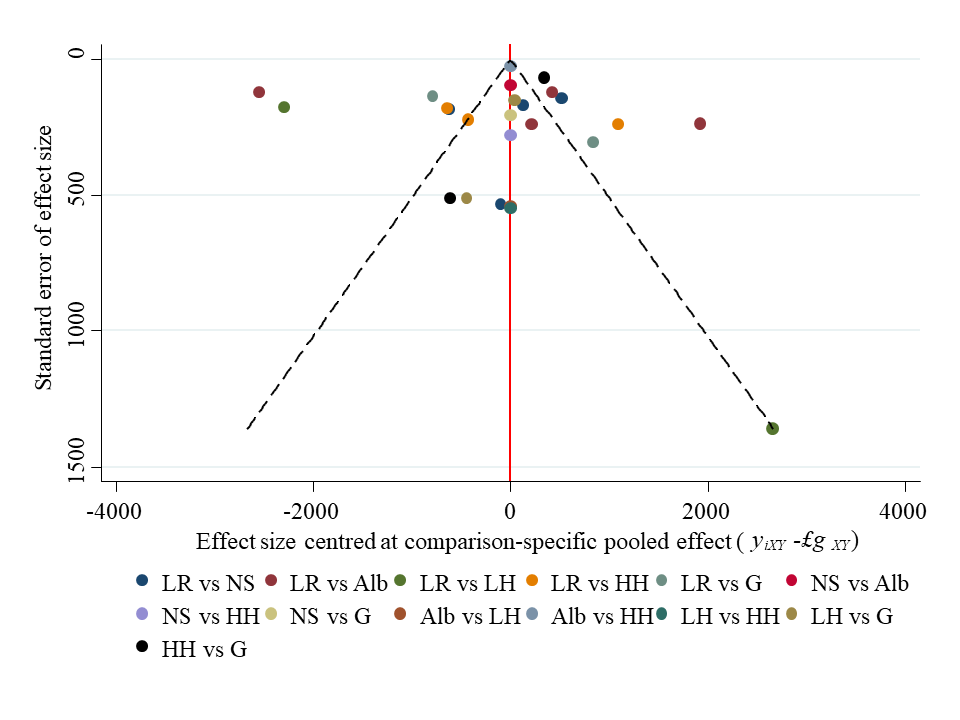


*BC, Balanced crystalloids; Alb. albumin, LH, L-HES, HH, H-HES, G, gelatin.

| Egger's test for small-study effects: | | | | | | |
| --- | --- | --- | --- | --- | --- | --- |
| Std_Eff | Coef. | Std. Err. | t | P>t | [95% Conf. | Interval] |
| slope | -12.8139 | 172.3072 | -0.07 | 0.941 | -369.259 | 343.6308 |
| bias | -0.69707 | 1.626656 | -0.43 | 0.672 | -4.06206 | 2.667927 |
| Test of H0: no small-study effects P = 0.692 | | | | | | |

### 10.2.3. Acute kidney injury in surgical patients


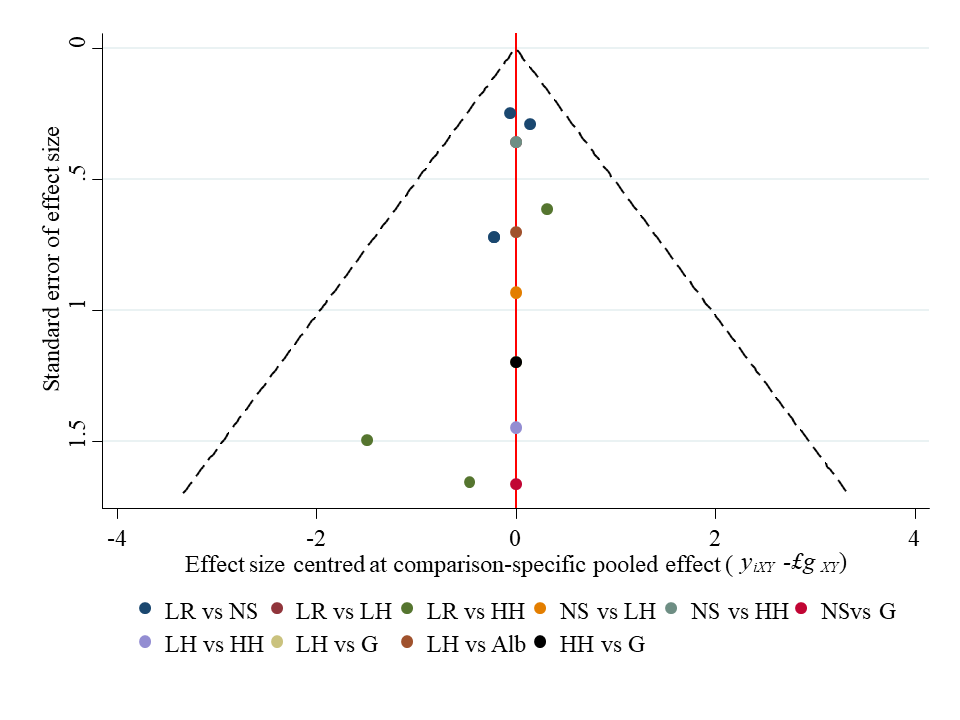


*BC, Balanced crystalloids; Alb. albumin, LH, L-HES, HH, H-HES, G, gelatin.

| Egger's test for small-study effects: | | | | | | |
| --- | --- | --- | --- | --- | --- | --- |
| Std_Eff | Coef. | Std. Err. | t | P>t | [95% Conf. | Interval] |
| slope | 0.099143 | 0.08355 | 1.19 | 0.257 | -0.08136 | 0.279641 |
| bias | -0.23105 | 0.16189 | -1.43 | 0.177 | -0.5808 | 0.118687 |
| Test of H0: no small-study effects P = 0.177 | | | | | | |

### 10.2.4. Blood transfusion among in surgical patients


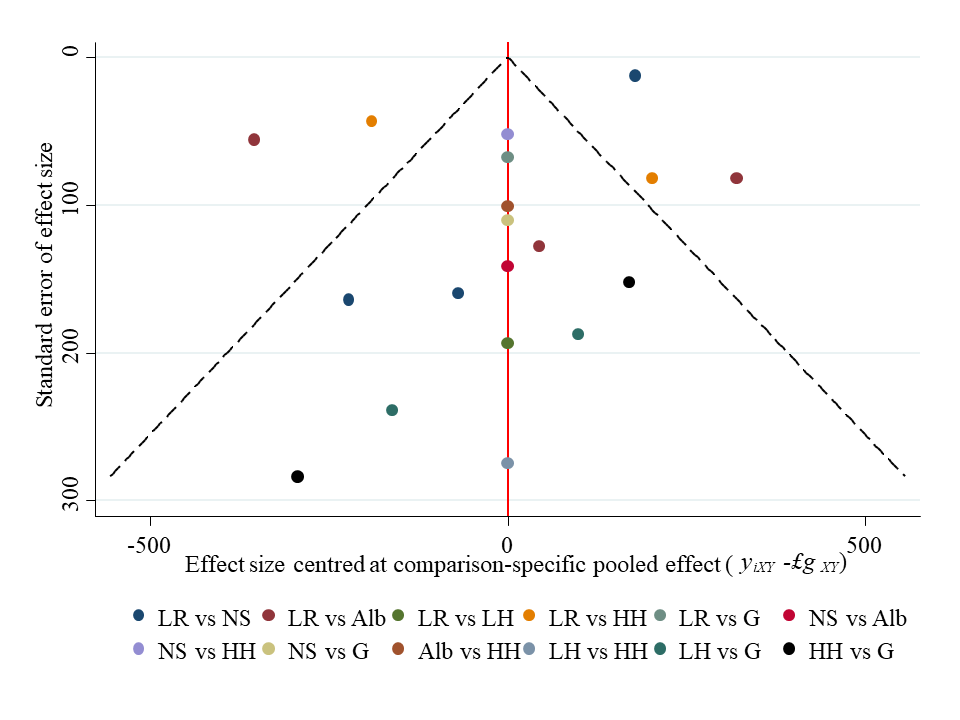


*BC, Balanced crystalloids; Alb. albumin, LH, L-HES, HH, H-HES, G, gelatin.

| Egger's test for small-study effects: | | | | | | |
| --- | --- | --- | --- | --- | --- | --- |
| Std_Eff | Coef. | Std. Err. | t | P>t | [95% Conf. | Interval] |
| slope | 168.3354 | 38.97644 | 4.32 | 0 | 86.10232 | 250.5685 |
| bias | -1.81156 | 0.832122 | -2.18 | 0.044 | -3.56719 | -0.05594 |
| Test of H0: no small-study effects P = 0.044 | | | | | | |

## 10.3. Publication bias in trauma patients

### 10.3.1.Mortality in trauma patients


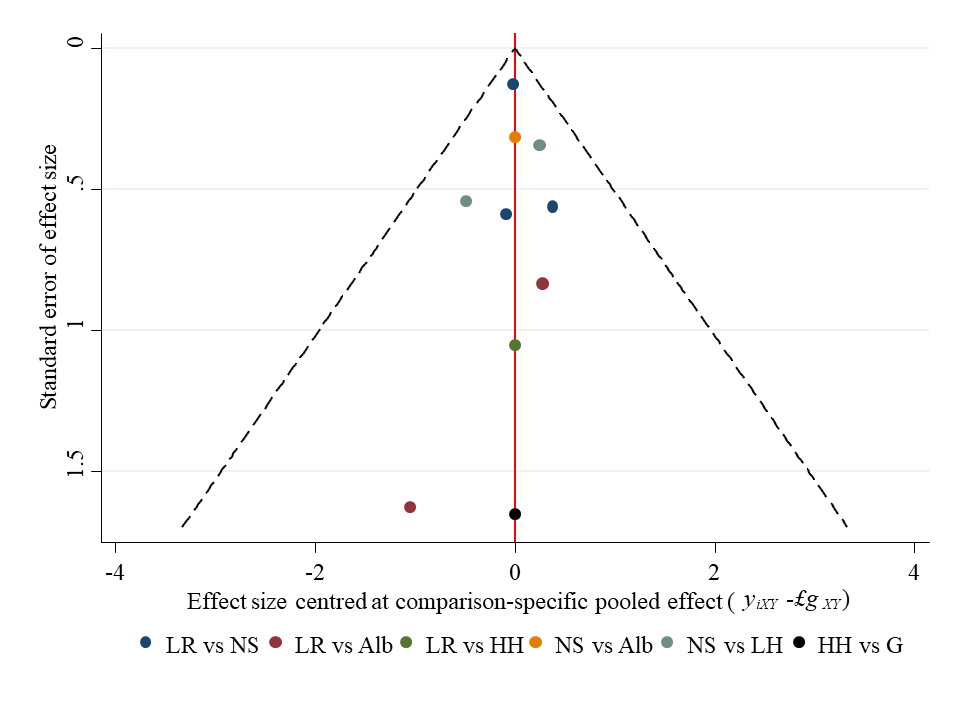


*BC, Balanced crystalloids; Alb. albumin, LH, L-HES, HH, H-HES, G, gelatin.

| Egger's test for small-study effects: | | | | | | |
| --- | --- | --- | --- | --- | --- | --- |
| Std_Eff | Coef. | Std. Err. | t | P>t | [95% Conf. | Interval] |
| slope | 0.015083 | 0.083482 | 0.18 | 0.861 | -0.17743 | 0.207592 |
| bias | -0.04262 | 0.255301 | -0.17 | 0.872 | -0.63135 | 0.546104 |
| Test of H0: no small-study effects P = 0.872 | | | | | | |

### 10.3.2. Mortality in traumatic brain injury patients

**
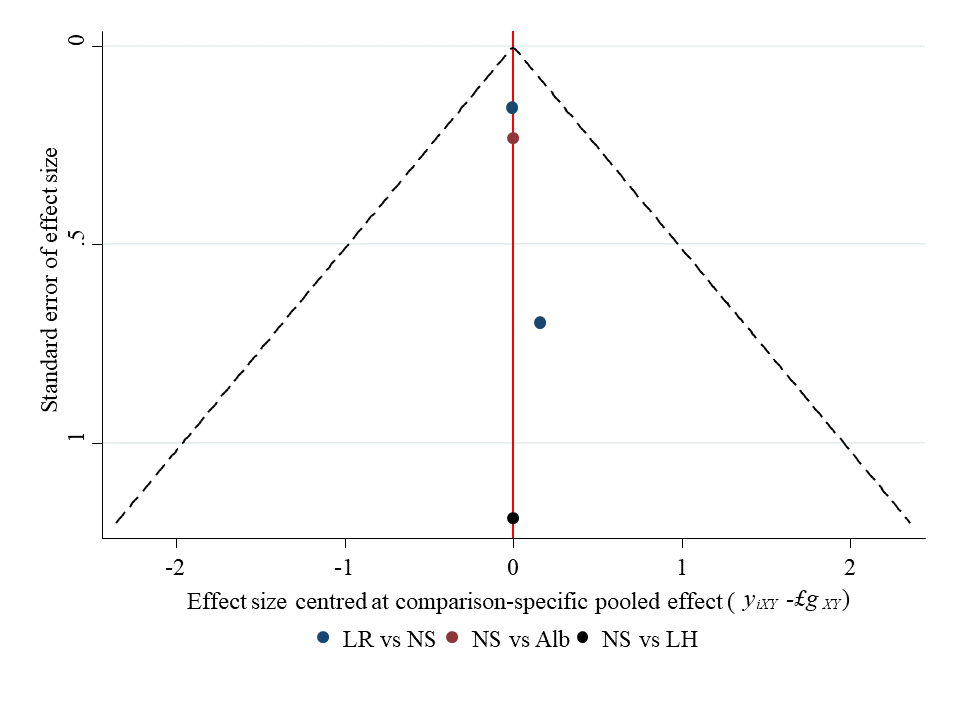
**

*BC, Balanced crystalloids; Alb. albumin, LH, L-HES, G, gelatin.

| Egger's test for small-study effects: | | | | | | |
| --- | --- | --- | --- | --- | --- | --- |
| Std_Eff | Coef. | Std. Err. | t | P>t | [95% Conf. | Interval] |
| slope | -0.02792 | 0.026961 | -1.04 | 0.409 | -0.14392 | 0.088079 |
| bias | 0.135699 | 0.107551 | 1.26 | 0.334 | -0.32706 | 0.598455 |
| Test of H0: no small-study effects P = 0.334 | | | | | | |

# Appendix 11: Inconsistency

.

| **Population** | **Outcome** | **Fit design-by-treatment interaction model** | **Explore Loop inconsistency** |
| --- | --- | --- | --- |
| **Sepsis patients** | **Mortality** | p=0.316 | p=0.843 |
|  | **Resuscitation fluid volume** | **p=0.018** | p=0.592 |
|  | **Acute kidney injury** | p=0.894 | p=0.894 |
|  | **Blood transfusion volume** | **p=0.018** | p=0.874 |
| **Surgical patients** | **Mortality** | p=0.962 | p=0.641 |
|  | **Resuscitation fluid volume** | p=0.311 | p=0.992 |
|  | **Acute kidney injury** | p=0.921 | p=0.921 |
|  | **Blood transfusion volume** | **p=0.019** | p=0.734 |
| **Traumatic patients** | **Mortality** | p=0.433 | p=0.433 |

## 11.1. Inconsistency in sepsis patients

### 11.1.1. Mortality in sepsis patients

eTable 11.1 Inconsistency between direct and indirect evidence

| Side | Direct | | Indirect | | Difference | | |
| --- | --- | --- | --- | --- | --- | --- | --- |
|  | Coef. | Std. Err. | Coef. | Std. Err. | Coef. | Std. Err. | P>z |
| A B | 0.17 | 0.06 | 0.20 | 0.14 | -0.02 | 0.16 | 0.865 |
| A C | -0.08 | 0.41 | 0.08 | 0.09 | -0.17 | 0.42 | 0.683 |
| A D | 0.15 | 0.12 | 0.26 | 0.11 | -0.11 | 0.17 | 0.52 |
| A E | 0.30 | 0.17 | 0.11 | 0.27 | 0.19 | 0.32 | 0.563 |
| A F | -0.44 | 0.34 | 0.43 | 0.26 | -0.88 | 0.50 | 0.08 |
| B C | -0.10 | 0.06 | 0.02 | 0.24 | -0.12 | 0.24 | 0.6 |
| B D | 0.02 | 0.07 | 0.06 | 0.14 | -0.04 | 0.16 | 0.776 |
| B E | 0.2 | 0.64 | 0.05 | 0.16 | 0.19 | 0.66 | 0.771 |
| B F | -0.18 | 0.18 | 0.05 | 0.23 | -0.23 | 0.25 | 0.353 |
| C D | -0.12 | 0.20 | 0.20 | 0.10 | -0.32 | 0.22 | 0.161 |
| C E | 0.10 | 0.53 | 0.17 | 0.17 | -0.07 | 0.56 | 0.901 |
| C F | -0.29 | 0.31 | 0.22 | 0.26 | -0.51 | 0.45 | 0.251 |
| D F | 0.01 | 0.20 | -0.35 | 0.24 | 0.37 | 0.27 | 0.181 |
| E F | 0.01 | 0.29 | -0.29 | 0.25 | 0.30 | 0.39 | 0.439 |

*A: lactate Ringer’s, B: Saline; C: Albumin, D: L-HES, E: H-HES, F: Gelatin.

eTable 11.2 Design inconsistency

*A: lactate Ringer’s, B: Saline; C: Albumin, D: L-HES, E: H-HES, F: Gelatin.

### 11.1.2. Resuscitation fluid volume in sepsis patients

eTable 11.3 Inconsistency between direct and indirect evidence

| Side | Direct | | Indirect | | Difference | | |
| --- | --- | --- | --- | --- | --- | --- | --- |
|  | Coef. | Std. Err. | Coef. | Std. Err. | Coef. | Std. Err. | P>z |
| A B * | -496.985 | 916.1906 | 1576.107 | 1469.408 | -2073.09 | 1730.869 | 0.231 |
| A C * | -2694.13 | 1644.455 | -1625.5 | 1441.46 | -1068.63 | 2187 | 0.625 |
| A D * | -494.898 | 1172.928 | -1207.58 | 1410.626 | 712.683 | 1834.073 | 0.698 |
| A F * | -1500 | 1550.594 | 2032.677 | 2742.498 | -3532.68 | 3155.074 | 0.263 |
| B C * | -2050.97 | 971.1009 | -2943.67 | 2390.965 | 892.7074 | 2594.766 | 0.731 |
| B D * | -1154.78 | 745.5403 | 683.0732 | 1720.299 | -1837.85 | 1875.713 | 0.327 |
| B E * | -213.919 | 917.698 | -169.566 | 9306.854 | -44.3528 | 9351.878 | 0.996 |
| B F * | 500 | 1504.685 | -3492.22 | 2272.243 | 3992.218 | 2725.176 | 0.143 |
| C D * | 1519.782 | 1193.53 | 865.7822 | 1701.767 | 653.9998 | 2082.954 | 0.754 |
| C F * | 1200 | 1660.153 | 2325.746 | 3137.669 | -1125.75 | 3549.698 | 0.751 |
| D F * | 0.000032 | 1668.057 | 508.6917 | 2665.411 | -508.692 | 3144.219 | 0.871 |

*A: lactate Ringer’s, B: Saline; C: Albumin, D: L-HES, E: H-HES, F: Gelatin.

eTable 11.4 Design inconsistency

*A: lactate Ringer’s, B: Saline; C: Albumin, D: L-HES, E: H-HES, F: Gelatin.

11.1.3. Acute kidney injury in sepsis patients

eTable 11.5 Inconsistency between direct and indirect evidence

| Side | Direct | | Indirect | | Difference | | |
| --- | --- | --- | --- | --- | --- | --- | --- |
|  | Coef. | Std. Err. | Coef. | Std. Err. | Coef. | Std. Err. | P>z |
| A B | 0.010596 | 0.103512 | 0.04026 | 0.190128 | -0.02966 | 0.21648 | 0.891 |
| A D | 0.241036 | 0.149311 | 0.194085 | 0.159364 | 0.046952 | 0.218382 | 0.83 |
| A E | 0.595034 | 0.194325 | 1.11861 | 1.205069 | -0.52358 | 1.220636 | 0.668 |
| B C * | 0.035074 | 0.147672 | -0.03451 | 162.3595 | 0.069587 | 162.3596 | 1 |
| B D | 0.187179 | 0.121488 | 0.23413 | 0.181471 | -0.04695 | 0.218382 | 0.83 |
| B E | 1.098599 | 1.201846 | 0.574732 | 0.214647 | 0.523867 | 1.220864 | 0.668 |
| E F * | -0.84202 | 0.38785 | -1.28565 | 134.9733 | 0.443633 | 134.9737 | 0.997 |

*A: lactate Ringer’s, B: Saline; C: Albumin, D: L-HES, E: H-HES, F: Gelatin.

eTable 11.6 Design inconsistency

*A: lactate Ringer’s, B: Saline; C: Albumin, D: L-HES, E: H-HES, F: Gelatin.

### 11.1.4. Blood transfusion among in sepsis patients

eTable 11.7 Inconsistency between direct and indirect evidence,

| Side | Direct | | Indirect | | Difference | | |
| --- | --- | --- | --- | --- | --- | --- | --- |
|  | Coef. | Std. Err. | Coef. | Std. Err. | Coef. | Std. Err. | P>z |
| A B | -6.79281 | 131.565 | 108.5036 | 189.6027 | -115.296 | 230.6788 | 0.617 |
| A D | 285 | 166.5297 | 168.485 | 159.8738 | 116.515 | 230.8501 | 0.614 |
| B C | 160.9539 | 94.91085 | -22.2159 | 190.5038 | 183.1698 | 212.1683 | 0.388 |
| B D | 154.6161 | 98.73383 | 275.856 | 144.2363 | -121.24 | 174.2392 | 0.487 |
| C D | 182.0919 | 115.5564 | -100.08 | 133.1702 | 282.1722 | 176.3485 | 0.11 |

*A: lactate Ringer’s, B: Saline; C: Albumin, D: L-HES, E: H-HES, F: Gelatin.

eTable 11.8 Design inconsistency

*A: lactate Ringer’s, B: Saline; C: Albumin, D: L-HES, E: H-HES, F: Gelatin.

## 11.2. Inconsistency in surgical patients

### 11.2.1. Mortality in surgical patients

eTable 11.9 Inconsistency between direct and indirect evidence

| Side | Direct | | Indirect | | Difference | | |
| --- | --- | --- | --- | --- | --- | --- | --- |
|  | Coef. | Std. Err. | Coef. | Std. Err. | Coef. | Std. Err. | P>z |
| A B | 0.078527 | 0.194263 | 0.62111 | 1.014568 | -0.54258 | 1.033029 | 0.599 |
| A C | -0.11042 | 0.393106 | -0.08307 | 0.964274 | -0.02736 | 1.02665 | 0.979 |
| A D | 0.151687 | 0.363061 | -0.31448 | 0.971552 | 0.466167 | 1.037923 | 0.653 |
| A E | 1.407301 | 1.693124 | -0.22047 | 0.694804 | 1.627774 | 1.854839 | 0.38 |
| A F | -0.18254 | 0.435489 | 0.654201 | 0.726597 | -0.83674 | 0.843829 | 0.321 |
| B C | -0.70291 | 1.621393 | -0.17541 | 0.418392 | -0.5275 | 1.660857 | 0.751 |
| B E | -0.00866 | 1.008621 | -0.10812 | 0.824614 | 0.099457 | 1.302806 | 0.939 |
| B F | -1.14862 | 1.663343 | 0.009681 | 0.422969 | -1.1583 | 1.716277 | 0.5 |
| C D | 0.291896 | 0.431356 | -0.19777 | 0.899437 | 0.489666 | 0.994983 | 0.623 |
| C E | 1.437253 | 1.692333 | -0.13567 | 0.754137 | 1.572918 | 1.873056 | 0.401 |
| C F | 8.18E-13 | 0.468293 | 0.70658 | 0.926138 | -0.70658 | 1.0378 | 0.496 |
| D E | 0.1302 | 1.554796 | -0.12301 | 0.791673 | 0.25321 | 1.819957 | 0.889 |
| D F | 0.008169 | 0.416709 | -0.47304 | 1.03799 | 0.481206 | 1.114116 | 0.666 |
| E F | 0.890268 | 0.911038 | -0.82239 | 0.884038 | 1.71266 | 1.255361 | 0.172 |

*A: lactate Ringer’s, B: Saline; C: Albumin, D: L-HES, E: H-HES, F: Gelatin.

eTable 11.10 Design inconsistency

*A: lactate Ringer’s, B: Saline; C: Albumin, D: L-HES, E: H-HES, F: Gelatin.

### 11.2.2. Resuscitation fluid volume in surgical patients

eTable 11.11 Inconsistency between direct and indirect evidence

| Side | Direct |  | Indirect |  | Difference | | |
| --- | --- | --- | --- | --- | --- | --- | --- |
|  | Coef. | Std. Err. | Coef. | Std. Err. | Coef. | Std. Err. | P>z |
| A B | 44.11102 | 599.181 | -1358.67 | 909.9829 | 1402.78 | 1089.758 | 0.198 |
| A C | -3156.5 | 533.7959 | -835.595 | 1053.819 | -2320.9 | 1183.33 | 0.05 |
| A D | -1803.65 | 1049.341 | -1641.8 | 833.7913 | -161.853 | 1338.935 | 0.904 |
| A E | -1402.58 | 718.3888 | -1522.22 | 810.835 | 119.6419 | 1084.701 | 0.912 |
| A F | -1042.6 | 882.052 | -1292.87 | 746.6267 | 250.2709 | 1155.345 | 0.829 |
| B C | -134.92 | 983.0974 | -3317.56 | 678.4799 | 3182.637 | 1194.538 | 0.008 |
| B E | -655.999 | 1251.616 | -1227.22 | 761.6242 | 571.2206 | 1465.133 | 0.697 |
| B F | -994.992 | 1240.635 | -728.319 | 800.3531 | -266.673 | 1476.395 | 0.857 |
| C D | 221.9924 | 1307.645 | 1340.81 | 884.2438 | -1118.82 | 1578.55 | 0.478 |
| C E | 894.9769 | 1221.275 | 1389.107 | 829.7572 | -494.13 | 1476.485 | 0.738 |
| D E | -468.367 | 1332.766 | 550.6505 | 872.7967 | -1019.02 | 1593.083 | 0.522 |
| D F | 325.6288 | 905.1955 | 775.5157 | 1056.061 | -449.887 | 1389.625 | 0.746 |
| E F | 528.5161 | 898.2159 | 17.6384 | 876.4219 | 510.8777 | 1254.919 | 0.684 |

*A: lactate Ringer’s, B: Saline; C: Albumin, D: L-HES, E: H-HES, F: Gelatin.

eTable 11.12 Design inconsistency

*A: lactate Ringer’s, B: Saline; C: Albumin, D: L-HES, E: H-HES, F: Gelatin.

### 11.2.3. Acute kidney injury in surgical patients

eTable 11.13 Inconsistency between direct and indirect evidence

| Side | Direct | | Indirect | | Difference | | |
| --- | --- | --- | --- | --- | --- | --- | --- |
|  | Coef. | Std. Err. | Coef. | Std. Err. | Coef. | Std. Err. | P>z |
| AB | 0.047502 | 0.176843 | 0.445654 | 0.525081 | -0.39815 | 0.554061 | 0.472 |
| AD | -0.25888 | 0.360621 | -0.19804 | 0.788251 | -0.06084 | 0.866826 | 0.944 |
| AE | 0.683028 | 0.537002 | 0.081865 | 0.384555 | 0.601164 | 0.660494 | 0.363 |
| BD | -0.48243 | 0.93373 | -0.31168 | 0.384293 | -0.17075 | 1.009719 | 0.866 |
| BE | 0.054067 | 0.358314 | 0.507969 | 0.526937 | -0.4539 | 0.637222 | 0.476 |
| BF | 1.148598 | 1.663343 | 1.192382 | 0.988552 | -0.04378 | 1.934935 | 0.982 |
| CD | 0.377294 | 0.703006 | -0.49021 | 221.0472 | 0.867508 | 221.0481 | 0.997 |
| DE | -5.93E-13 | 1.449138 | 0.587603 | 0.459142 | -0.5876 | 1.520135 | 0.699 |
| DF | 1.329247 | 1.18043 | 1.790509 | 1.453231 | -0.46126 | 1.974224 | 0.815 |
| EF | 1.199587 | 1.180726 | 0.677357 | 1.433401 | 0.52223 | 1.960021 | 0.79 |

*A: lactate Ringer’s, B: Saline; C: Albumin, D: L-HES, E: H-HES, F: Gelatin.

eTable 11.14 Design inconsistency

*A: lactate Ringer’s, B: Saline; C: Albumin, D: L-HES, E: H-HES, F: Gelatin.

### 11.2.4. Blood transfusion among in surgical patients

eTable 11.15 Inconsistency between direct and indirect evidence

| Side | Direct | | Indirect | | Difference | | |
| --- | --- | --- | --- | --- | --- | --- | --- |
|  | Coef. | Std. Err. | Coef. | Std. Err. | Coef. | Std. Err. | P>z |
| A B | 157.9727 | 155.5423 | 49.03422 | 211.4104 | 108.9384 | 261.6928 | 0.677 |
| A C * | -206.649 | 147.4266 | -132.526 | 420.1876 | -74.1235 | 446.0371 | 0.868 |
| A D | 1.29E-07 | 302.391 | -223.592 | 248.9501 | 223.5915 | 391.6841 | 0.568 |
| A E | -168.617 | 141.6973 | 300.8304 | 162.316 | -469.447 | 214.7756 | 0.029 |
| A F | -40 | 246.8124 | -145.71 | 184.8458 | 105.71 | 308.3575 | 0.732 |
| B C | -612.957 | 253.3508 | -140.967 | 190.1957 | -471.99 | 317.5631 | 0.137 |
| B E | 43.00018 | 240.6701 | -148.619 | 181.9549 | 191.6196 | 301.7112 | 0.525 |
| B F | -150 | 262.2123 | -273.787 | 202.3238 | 123.7869 | 331.1957 | 0.709 |
| C E | 150 | 258.8474 | 305.2647 | 223.6025 | -155.265 | 342.0527 | 0.65 |
| D E | 275.174 | 358.3434 | 124.328 | 247.4314 | 150.8459 | 438.161 | 0.731 |
| D F | 104.8682 | 222.4281 | -147.413 | 327.3566 | 252.2814 | 396.556 | 0.525 |
| E F | -288.968 | 218.1554 | -23.2232 | 204.9668 | -265.745 | 298.5469 | 0.373 |

*A: lactate Ringer’s, B: Saline; C: Albumin, D: L-HES, E: H-HES, F: Gelatin.

eTable 11.16 Design inconsistency

*A: lactate Ringer’s, B: Saline; C: Albumin, D: L-HES, E: H-HES, F: Gelatin.

## 11.3. Inconsistency in traumatic patients

### 11.3.1. Mortality in traumatic patients

eTable 11.17 Inconsistency between direct and indirect evidence

| Side | Direct |  | Indirect |  | Difference |  |  |
| --- | --- | --- | --- | --- | --- | --- | --- |
|  | Coef. | Std. Err. | Coef. | Std. Err. | Coef. | Std. Err. | P>z |
| A B | 0.041396 | 0.120748 | 0.681782 | 0.807057 | -0.64039 | 0.81604 | 0.433 |
| A C | 0.682338 | 0.74312 | 0.041825 | 0.337344 | 0.640514 | 0.816106 | 0.433 |
| A E * | -0.05407 | 1.052705 | 0.405466 | 177.6164 | -0.45953 | 177.6195 | 0.998 |
| B C | 0.000429 | 0.314994 | 0.640937 | 0.752864 | -0.64051 | 0.816104 | 0.433 |
| B D * | 0.233859 | 0.291366 | -0.09058 | 140.7204 | 0.324435 | 140.7208 | 0.998 |
| E F * | -1.09861 | 1.653621 | -0.17955 | 355.0909 | -0.91906 | 355.091 | 0.998 |

*A: lactate Ringer’s, B: Saline; C: Albumin, D: L-HES, E: H-HES, F: Gelatin.

eTable 11.18 Design inconsistency

*A: lactate Ringer’s, B: Saline; C: Albumin, D: L-HES, E: H-HES, F: Gelatin.

# Appendix 12: Meta-regression

## 12.1. SUCRA and mean ranks changes before and after model adjust in sepsis trials

| Covariate/ SUCRA | BC | Saline | Albumin | L-HES | H-HES | Gelatin |
| --- | --- | --- | --- | --- | --- | --- |
| Unadjusted model | 88.6 | 34.1 | 67.9 | 24.3 | 22.7 | 62.5 |
| Age | 72.4 | 59.3 | 45.6 | 38 | 40.3 | 44.4 |
| Male percentage | 40.6 | 38.2 | 50.5 | 83.2 | 28.1 | 59.4 |
| Mean arterial pressure | 18.6 | 52.1 | 32.3 | 85.3 | 35.1 | 76.6 |
| Vasopressor percentage | 60.5 | 51.9 | 49.4 | 41 | 50.9 | 46.3 |
| Lactate level | 5.5 | 61 | 60.1 | 87.6 | 63.4 | 22.4 |

| Covariate/ Mean rank | BC | Saline | Albumin | L-HES | H-HES | Gelatin |
| --- | --- | --- | --- | --- | --- | --- |
| Unadjusted model | 1.6 | 4.3 | 2.6 | 4.8 | 4.9 | 2.9 |
| Age | 2.4 | 3 | 3.7 | 4.1 | 4 | 3.8 |
| Male percentage | 4 | 4.1 | 3.5 | 1.8 | 4.6 | 3 |
| Mean arterial pressure | 5.1 | 3.4 | 4.4 | 1.7 | 4.2 | 2.2 |
| Vasopressor percentage | 3 | 3.4 | 3.5 | 4 | 3.5 | 3.7 |
| Lactate level | 5.7 | 3 | 3 | 1.6 | 2.8 | 4.9 |

## 12.2. Significance for meta-regression model in sepsis trials

| Covariate | BC | Saline | Albumin | L-HES | H-HES | Gelatin |
| --- | --- | --- | --- | --- | --- | --- |
| Age | reference | 0.892 | 0.445 | 0.489 | 0.752 | 0.788 |
| Male percentage | reference | 0.461 | 0.678 | 0.251 | 0.447 | 0.115 |
| Mean arterial pressure | reference | 0.461 | 0.678 | 0.251 | 0.446 | 0.115 |
| Vasopressor percentage | reference | 0.898 | 0.994 | 0.834 | 0.996 | 0.985 |
| Lactate level | reference | 0.097 | 0.100 | 0.072 | 0.098 | 0.17 |

# Appendix 13: Grading the evidence using CINeMA web application

## 13.1. Sepsis patients

### 13.1.1 Confidence rating in sepsis trails for mortality

eTable 13.1. Grading the evidence in sepsis patients for mortality

| **Comparison** | **Studies** | **Within-study bias** | **Reporting bias** | **Indirectness** | **Imprecision** | **Heterogeneity** | **Incoherence** | **Confidence rating** |
| --- | --- | --- | --- | --- | --- | --- | --- | --- |
| **BC:Saline** | 3 | Some concerns | Undetected | No concerns | No concerns | No concerns | No concerns | **Moderate** |
| **BC:Iso-Alb** | 0 | No concerns | Undetected | No concerns | Major concerns | No concerns | No concerns | **Low** |
| **Saline:Iso-Alb** | 2 | No concerns | Undetected | No concerns | Some concerns | No concerns | No concerns | **Moderate** |
| **BC:Hyper-Alb** | 1 | Some concerns | Undetected | No concerns | Some concerns | No concerns | No concerns | **Low** |
| **Saline:Hyper-Alb** | 4 | No concerns | Undetected | No concerns | No concerns | Some concerns | No concerns | **Moderate** |
| **Iso-Alb:Hyper-Alb** | 0 | No concerns | Undetected | No concerns | Some concerns | No concerns | No concerns | **Moderate** |
| **BC:L-HES** | 2 | No concerns | Undetected | No concerns | No concerns | No concerns | No concerns | **High** |
| **Saline:L-HES** | 8 | No concerns | Undetected | No concerns | No concerns | No concerns | No concerns | **High** |
| **Iso-Alb:L-HES** | 1 | No concerns | Undetected | No concerns | Some concerns | No concerns | No concerns | **Moderate** |
| **Hyper-Alb:L-HES** | 4 | No concerns | Undetected | No concerns | Some concerns | No concerns | No concerns | **Moderate** |
| **BC:H-HES** | 1 | Some concerns | Undetected | No concerns | Some concerns | No concerns | No concerns | **Low** |
| **Saline:H-HES** | 1 | Some concerns | Undetected | No concerns | Major concerns | No concerns | No concerns | **Very low** |
| **Iso-Alb:H-HES** | 2 | Some concerns | Undetected | No concerns | Some concerns | No concerns | No concerns | **Low** |
| **Hyper-Alb:H-HES** | 0 | Some concerns | Undetected | No concerns | Some concerns | Some concerns | No concerns | **Very low** |
| **L-HES:H-HES** | 0 | Some concerns | Undetected | No concerns | Major concerns | No concerns | No concerns | **Very low** |
| **BC:Gelatin** | 1 | Some concerns | Undetected | No concerns | Major concerns | No concerns | Major concerns | **Very low** |
| **Saline:Gelatin** | 1 | Some concerns | Undetected | No concerns | Major concerns | No concerns | No concerns | **Very low** |
| **Iso-Alb:Gelatin** | 0 | Some concerns | Undetected | No concerns | Major concerns | No concerns | No concerns | **Very low** |
| **Hyper-Alb:Gelatin** | 1 | Some concerns | Undetected | No concerns | Major concerns | No concerns | No concerns | **Very low** |
| **L-HES:Gelatin** | 1 | Some concerns | Undetected | No concerns | Some concerns | Some concerns | No concerns | **Very low** |
| **H-HES:Gelatin** | 3 | Some concerns | Undetected | No concerns | Some concerns | Some concerns | No concerns | **Very low** |

Abbreviations: BC, balanced crystalloids; Iso-Alb, iso-oncotic albumin; Hyper-Alb, hyperoncotic albumin; L-HES, low molecular weight hydroxyethyl starch; H-HES, high molecular weight hydroxyethyl starch.

### 13.1.2 Confidence rating in sepsis trails for fluid resuscitation volume

eTable 13.2. Grading the evidence in sepsis patients for fluid resuscitation volume

| **Comparison** | **Studies** | **Within-study bias** | **Reporting bias** | **Indirectness** | **Imprecision** | **Heterogeneity** | **Incoherence** | **Confidence rating** |
| --- | --- | --- | --- | --- | --- | --- | --- | --- |
| **BC:Saline** | 3 | Some concerns | Undetected | No concerns | Major concerns | No concerns | No concerns | **Very low** |
| **BC:Iso-Alb** | 0 | Some concerns | Undetected | No concerns | No concerns | No concerns | Major concerns | **Very low** |
| **Saline:Iso-Alb** | 2 | No concerns | Undetected | No concerns | No concerns | No concerns | Major concerns | **Low** |
| **BC:Hyper-Alb** | 1 | Some concerns | Undetected | No concerns | No concerns | No concerns | Major concerns | **Very low** |
| **Saline:Hyper-Alb** | 1 | Some concerns | Undetected | No concerns | No concerns | No concerns | Major concerns | **Very low** |
| **Iso-Alb:Hyper-Alb** | 0 | Some concerns | Undetected | No concerns | Major concerns | No concerns | Major concerns | **Very low** |
| **BC:L-HES** | 2 | Some concerns | Undetected | No concerns | Major concerns | No concerns | No concerns | **Very low** |
| **Saline:L-HES** | 6 | Some concerns | Undetected | No concerns | Major concerns | No concerns | Some concerns | **Very low** |
| **Iso-Alb:L-HES** | 1 | Some concerns | Undetected | No concerns | No concerns | Major concerns | No concerns | **Very low** |
| **Hyper-Alb:L-HES** | 1 | Some concerns | Undetected | No concerns | No concerns | Major concerns | No concerns | **Very low** |
| **BC:H-HES** | 0 | Some concerns | Undetected | No concerns | No concerns | Major concerns | Major concerns | **Very low** |
| **Saline:H-HES** | 1 | No concerns | Undetected | No concerns | No concerns | Major concerns | Major concerns | **Very low** |
| **Iso-Alb:H-HES** | 1 | Some concerns | Undetected | No concerns | Major concerns | No concerns | Major concerns | **Very low** |
| **Hyper-Alb:H-HES** | 0 | Some concerns | Undetected | No concerns | Major concerns | No concerns | Major concerns | **Very low** |
| **L-HES:H-HES** | 0 | No concerns | Undetected | No concerns | Major concerns | No concerns | Major concerns | **Very low** |
| **BC:Gelatin** | 1 | Some concerns | Undetected | No concerns | Major concerns | No concerns | Major concerns | **Very low** |
| **Saline:Gelatin** | 1 | Some concerns | Undetected | No concerns | Major concerns | No concerns | Major concerns | **Very low** |
| **Iso-Alb:Gelatin** | 0 | Some concerns | Undetected | No concerns | No concerns | Major concerns | Major concerns | **Very low** |
| **Hyper-Alb:Gelatin** | 1 | Some concerns | Undetected | No concerns | No concerns | Major concerns | Major concerns | **Very low** |
| **L-HES:Gelatin** | 1 | Some concerns | Undetected | No concerns | Major concerns | No concerns | No concerns | **Very low** |
| **H-HES:Gelatin** | 0 | Some concerns | Undetected | No concerns | Major concerns | No concerns | Major concerns | **Very low** |

Abbreviations: BC, balanced crystalloids; Iso-Alb, iso-oncotic albumin; Hyper-Alb, hyperoncotic albumin; L-HES, low molecular weight hydroxyethyl starch; H-HES, high molecular weight hydroxyethyl starch.

### 13.1.3 Confidence rating in sepsis trails for acute kidney injury

eTable 13.3. Grading the evidence in sepsis patients for acute kidney injury

| **Comparison** | **Studies** | **Within-study bias** | **Reporting bias** | **Indirectness** | **Imprecision** | **Heterogeneity** | **Incoherence** | **Confidence rating** |
| --- | --- | --- | --- | --- | --- | --- | --- | --- |
| **BC:Saline** | 2 | Some concerns | Undetected | No concerns | No concerns | Some concerns | No concerns | **Low** |
| **BC:Iso-Alb** | 0 | No concerns | Undetected | No concerns | Major concerns | No concerns | No concerns | **Very low** |
| **Saline:Iso-Alb** | 1 | No concerns | Undetected | No concerns | Major concerns | No concerns | No concerns | **Low** |
| **BC:L-HES** | 1 | No concerns | Undetected | No concerns | No concerns | Some concerns | No concerns | **Moderate** |
| **Saline:L-HES** | 4 | No concerns | Undetected | No concerns | No concerns | Some concerns | No concerns | **Moderate** |
| **Iso-Alb:L-HES** | 0 | No concerns | Undetected | No concerns | Some concerns | Some concerns | No concerns | **Low** |
| **BC:H-HES** | 1 | Some concerns | Undetected | No concerns | No concerns | No concerns | No concerns | **Moderate** |
| **Saline H-HES** | 1 | Some concerns | Undetected | No concerns | No concerns | No concerns | No concerns | **Moderate** |
| **Iso-Alb H-HES** | 0 | Some concerns | Undetected | No concerns | No concerns | Some concerns | No concerns | **Low** |
| **L-HES H-HES** | 0 | Some concerns | Undetected | No concerns | Some concerns | No concerns | No concerns | **Low** |
| **BC:Gelatin** | 0 | Some concerns | Undetected | No concerns | Major concerns | No concerns | No concerns | **Very low** |
| **Gelatin:Saline** | 0 | Some concerns | Undetected | No concerns | Major concerns | No concerns | No concerns | **Very low** |
| **Gelatin:Iso-Alb** | 0 | Some concerns | Undetected | No concerns | Major concerns | No concerns | No concerns | **Very low** |
| **Gelatin:L-HES** | 0 | Some concerns | Undetected | No concerns | Major concerns | No concerns | No concerns | **Very low** |
| **Gelatin:H-HES** | 1 | Major concerns | Undetected | No concerns | No concerns | Some concerns | No concerns | **Very low** |

Abbreviations: BC, balanced crystalloids; Iso-Alb, iso-oncotic albumin; Hyper-Alb, hyperoncotic albumin; L-HES, low molecular weight hydroxyethyl starch; H-HES, high molecular weight hydroxyethyl starch.

### 13.1.4 Confidence rating in sepsis trails for blood transfusion volume

eTable 13.4. Grading the evidence in sepsis patients for blood transfusion volume

| **Comparison** | **Studies** | **Within-study bias** | **Reporting bias** | **Indirectness** | **Imprecision** | **Heterogeneity** | **Incoherence** | **Confidence rating** |
| --- | --- | --- | --- | --- | --- | --- | --- | --- |
| **BC:H-HES** | 1 | Some concerns | Undetected | No concerns | No concerns | Major concerns | Major concerns | **Very low** |
| **BC:L-HES** | 1 | No concerns | Undetected | No concerns | No concerns | Major concerns | No concerns | **Low** |
| **BC:Saline** | 2 | Some concerns | Undetected | No concerns | Major concerns | No concerns | No concerns | **Very low** |
| **Hyper-Alb:L-HES** | 1 | Some concerns | Undetected | No concerns | Major concerns | No concerns | No concerns | **Very low** |
| **Hyper-Alb:Saline** | 1 | No concerns | Undetected | No concerns | No concerns | Major concerns | No concerns | **Low** |
| **Iso-Alb:L-HES** | 1 | Some concerns | Undetected | No concerns | Major concerns | No concerns | No concerns | **Very low** |
| **Iso-Alb:Saline** | 2 | No concerns | Undetected | No concerns | Major concerns | No concerns | No concerns | **Low** |
| **L-HES:Saline** | 3 | No concerns | Undetected | No concerns | No concerns | Major concerns | No concerns | **Low** |
| **BC:Hyper-Alb** | 0 | No concerns | Undetected | No concerns | Some concerns | Some concerns | Major concerns | **Very low** |
| **BC:Iso-Alb** | 0 | Some concerns | Undetected | No concerns | Major concerns | No concerns | Major concerns | **Very low** |
| **H-HES:Hyper-Alb** | 0 | Some concerns | Undetected | No concerns | Major concerns | No concerns | Major concerns | **Very low** |
| **H-HES:Iso-Alb** | 0 | Some concerns | Undetected | No concerns | Some concerns | Some concerns | Major concerns | **Very low** |
| **H-HES:L-HES** | 0 | Some concerns | Undetected | No concerns | Major concerns | No concerns | Major concerns | **Very low** |
| **H-HES:Saline** | 0 | Some concerns | Undetected | No concerns | No concerns | Major concerns | Major concerns | **Very low** |
| **Hyper-Alb:Iso-Alb** | 0 | No concerns | Undetected | No concerns | Major concerns | No concerns | Major concerns | **Very low** |

Abbreviations: BC, balanced crystalloids; Iso-Alb, iso-oncotic albumin; Hyper-Alb, hyperoncotic albumin; L-HES, low molecular weight hydroxyethyl starch; H-HES, high molecular weight hydroxyethyl starch.

## 13.2. Surgical patients

### 13.2.1 Confidence rating in surgical trails for mortality

eTable 13.5. Grading the evidence in surgical trials patients for mortality

| **Comparison** | **Studies** | **Within-study bias** | **Reporting bias** | **Indirectness** | **Imprecision** | **Heterogeneity** | **Incoherence** | **Confidence rating** |
| --- | --- | --- | --- | --- | --- | --- | --- | --- |
| **BC:Gelatin** | 3 | No concerns | Undetected | No concerns | Major concerns | No concerns | No concerns | **Low** |
| **BC:H-HES** | 1 | No concerns | Undetected | No concerns | Major concerns | No concerns | No concerns | **Low** |
| **BC:Hyper-Alb** | 1 | Some concerns | Undetected | No concerns | Major concerns | No concerns | No concerns | **Very low** |
| **BC:Iso-Alb** | 5 | No concerns | Undetected | No concerns | Major concerns | No concerns | No concerns | **Low** |
| **BC:L-HES** | 5 | No concerns | Undetected | No concerns | Major concerns | No concerns | No concerns | **Low** |
| **BC:Saline** | 4 | Some concerns | Undetected | No concerns | Major concerns | No concerns | No concerns | **Very low** |
| **Gelatin:H-HES** | 2 | No concerns | Undetected | No concerns | Major concerns | No concerns | No concerns | **Low** |
| **Gelatin:Iso-Alb** | 1 | No concerns | Undetected | No concerns | Major concerns | No concerns | No concerns | **Low** |
| **Gelatin:L-HES** | 3 | No concerns | Undetected | No concerns | Major concerns | No concerns | No concerns | **Low** |
| **Gelatin:Saline** | 1 | No concerns | Undetected | No concerns | Major concerns | No concerns | No concerns | **Low** |
| **H-HES:Hyper-Alb** | 1 | Some concerns | Undetected | No concerns | Major concerns | No concerns | No concerns | **Very low** |
| **H-HES:L-HES** | 1 | No concerns | Undetected | No concerns | Major concerns | No concerns | No concerns | **Low** |
| **H-HES:Saline** | 1 | No concerns | Undetected | No concerns | Major concerns | No concerns | No concerns | **Low** |
| **Iso-Alb:L-HES** | 2 | No concerns | Undetected | No concerns | Major concerns | No concerns | No concerns | **Low** |
| **Iso-Alb:Saline** | 1 | No concerns | Undetected | No concerns | Major concerns | No concerns | No concerns | **Low** |
| **L-HES:Saline** | 1 | No concerns | Undetected | No concerns | Major concerns | No concerns | No concerns | **Low** |
| **Gelatin:Hyper-Alb** | 0 | No concerns | Undetected | No concerns | Major concerns | No concerns | No concerns | **Low** |
| **H-HES:Iso-Alb** | 0 | No concerns | Undetected | No concerns | Major concerns | No concerns | No concerns | **Low** |
| **Hyper-Alb:Iso-Alb** | 0 | No concerns | Undetected | No concerns | Major concerns | No concerns | No concerns | **Low** |
| **Hyper-Alb:L-HES** | 0 | No concerns | Undetected | No concerns | Major concerns | No concerns | No concerns | **Low** |
| **Hyper-Alb:Saline** | 0 | Some concerns | Undetected | No concerns | Major concerns | No concerns | No concerns | **Very low** |

Abbreviations: BC, balanced crystalloids; Iso-Alb, iso-oncotic albumin; Hyper-Alb, hyperoncotic albumin; L-HES, low molecular weight hydroxyethyl starch; H-HES, high molecular weight hydroxyethyl starch.

### 13.2.2 Confidence rating in surgical trails for fluid resuscitation volume

eTable 13.6. Grading the evidence in surgical trials patients for fluid resuscitation volume

| **Comparison** | **Studies** | **Within-study bias** | **Reporting bias** | **Indirectness** | **Imprecision** | **Heterogeneity** | **Incoherence** | **Confidence rating** |
| --- | --- | --- | --- | --- | --- | --- | --- | --- |
| **BC:Gelatin** | 2 | No concerns | Undetected | No concerns | No concerns | Major concerns | No concerns | **Low** |
| **BC:H-HES** | 2 | No concerns | Undetected | No concerns | No concerns | Major concerns | No concerns | **Low** |
| **BC:Hyper-Alb** | 1 | Some concerns | Undetected | No concerns | No concerns | Major concerns | No concerns | **Very low** |
| **BC:Iso-Alb** | 3 | Some concerns | Undetected | No concerns | No concerns | Major concerns | No concerns | **Very low** |
| **BC:L-HES** | 3 | No concerns | Undetected | No concerns | No concerns | Major concerns | No concerns | **Low** |
| **BC:Saline** | 4 | Some concerns | Undetected | No concerns | Major concerns | No concerns | No concerns | **Very low** |
| **Gelatin:H-HES** | 2 | No concerns | Undetected | No concerns | Major concerns | No concerns | No concerns | **Low** |
| **Gelatin:L-HES** | 2 | No concerns | Undetected | No concerns | Major concerns | No concerns | No concerns | **Low** |
| **Gelatin:Saline** | 1 | No concerns | Undetected | No concerns | Major concerns | No concerns | No concerns | **Low** |
| **H-HES:Hyper-Alb** | 1 | Some concerns | Undetected | No concerns | Major concerns | No concerns | No concerns | **Very low** |
| **H-HES:L-HES** | 1 | No concerns | Undetected | No concerns | Major concerns | No concerns | No concerns | **Low** |
| **H-HES:Saline** | 1 | No concerns | Undetected | No concerns | Major concerns | No concerns | No concerns | **Low** |
| **Iso-Alb:L-HES** | 1 | No concerns | Undetected | No concerns | Major concerns | No concerns | No concerns | **Low** |
| **Iso-Alb:Saline** | 1 | Some concerns | Undetected | No concerns | No concerns | Major concerns | Major concerns | **Very low** |
| **L-HES:Saline** | 1 | No concerns | Undetected | No concerns | Major concerns | No concerns | No concerns | **Low** |
| **Gelatin:Hyper-Alb** | 0 | Some concerns | Undetected | No concerns | Major concerns | No concerns | Major concerns | **Very low** |
| **Gelatin:Iso-Alb** | 0 | Some concerns | Undetected | No concerns | Major concerns | No concerns | Major concerns | **Very low** |
| **H-HES:Iso-Alb** | 0 | Some concerns | Undetected | No concerns | Major concerns | No concerns | Major concerns | **Very low** |
| **Hyper-Alb:Iso-Alb** | 0 | Some concerns | Undetected | No concerns | Major concerns | No concerns | Major concerns | **Very low** |
| **Hyper-Alb:L-HES** | 0 | No concerns | Undetected | No concerns | Major concerns | No concerns | Major concerns | **Very low** |
| **Hyper-Alb:Saline** | 0 | Some concerns | Undetected | No concerns | No concerns | Major concerns | Major concerns | **Very low** |

Abbreviations: BC, balanced crystalloids; Iso-Alb, iso-oncotic albumin; Hyper-Alb, hyperoncotic albumin; L-HES, low molecular weight hydroxyethyl starch; H-HES, high molecular weight hydroxyethyl starch.

### 13.2.3 Confidence rating in surgical trails for adverse renal events

eTable 13.7. Grading the evidence in surgical trials patients for adverse renal events

| **Comparison** | **Studies** | **Within-study bias** | **Reporting bias** | **Indirectness** | **Imprecision** | **Heterogeneity** | **Incoherence** | **Confidence rating** |
| --- | --- | --- | --- | --- | --- | --- | --- | --- |
| **BC:Gelatin** | 1 | No concerns | Undetected | Some concerns | Major concerns | No concerns | No concerns | **Very low** |
| **BC:H-HES** | 1 | No concerns | Undetected | No concerns | Major concerns | No concerns | No concerns | **Low** |
| **BC:L-HES** | 4 | No concerns | Undetected | No concerns | Some concerns | Some concerns | No concerns | **Low** |
| **BC:Saline** | 3 | No concerns | Undetected | No concerns | Major concerns | No concerns | No concerns | **Low** |
| **Gelatin:H-HES** | 1 | No concerns | Undetected | No concerns | Major concerns | No concerns | No concerns | **Low** |
| **Gelatin:L-HES** | 1 | No concerns | Undetected | No concerns | Major concerns | No concerns | No concerns | **Low** |
| **Gelatin:Saline** | 1 | No concerns | Undetected | No concerns | Major concerns | No concerns | No concerns | **Low** |
| **H-HES:L-HES** | 1 | No concerns | Undetected | No concerns | Major concerns | No concerns | No concerns | **Low** |
| **H-HES:Saline** | 1 | No concerns | Undetected | No concerns | Major concerns | No concerns | No concerns | **Low** |
| **Iso-Alb:L-HES** | 1 | No concerns | Undetected | No concerns | Major concerns | No concerns | No concerns | **Low** |
| **L-HES:Saline** | 1 | No concerns | Undetected | No concerns | Some concerns | No concerns | No concerns | **Moderate** |
| **BC:Iso-Alb** | 0 | No concerns | Undetected | No concerns | Major concerns | No concerns | No concerns | **Low** |
| **Gelatin:Iso-Alb** | 0 | No concerns | Undetected | No concerns | Major concerns | No concerns | No concerns | **Low** |
| **H-HES:Iso-Alb** | 0 | No concerns | Undetected | No concerns | Major concerns | No concerns | No concerns | **Low** |
| **Iso-Alb:Saline** | 0 | No concerns | Undetected | No concerns | Major concerns | No concerns | No concerns | **Low** |

Abbreviations: BC, balanced crystalloids; Iso-Alb, iso-oncotic albumin; Hyper-Alb, hyperoncotic albumin; L-HES, low molecular weight hydroxyethyl starch; H-HES, high molecular weight hydroxyethyl starch.

### 13.2.4 Confidence rating in surgical trails for blood transfusion volume

eTable 13.8. Grading the evidence in surgical trials patients for blood transfusion volume

| **Comparison** | **Studies** | **Within-study bias** | **Reporting bias** | **Indirectness** | **Imprecision** | **Heterogeneity** | **Incoherence** | **Confidence rating** |
| --- | --- | --- | --- | --- | --- | --- | --- | --- |
| **BC:Gelatin** | 1 | No concerns | Undetected | No concerns | Major concerns | No concerns | No concerns | **Low** |
| **BC:H-HES** | 2 | No concerns | Undetected | No concerns | Major concerns | No concerns | Major concerns | **Very low** |
| **BC:Hyper-Alb** | 1 | Some concerns | Undetected | No concerns | No concerns | Major concerns | Major concerns | **Very low** |
| **BC:Iso-Alb** | 2 | Some concerns | Undetected | No concerns | Major concerns | No concerns | Major concerns | **Very low** |
| **BC:L-HES** | 1 | No concerns | Undetected | No concerns | Major concerns | No concerns | No concerns | **Low** |
| **BC:Saline** | 3 | No concerns | Undetected | No concerns | Major concerns | No concerns | No concerns | **Low** |
| **Gelatin:H-HES** | 2 | No concerns | Undetected | No concerns | Major concerns | No concerns | Some concerns | **Very low** |
| **Gelatin:L-HES** | 2 | No concerns | Undetected | No concerns | Major concerns | No concerns | No concerns | **Low** |
| **Gelatin:Saline** | 1 | No concerns | Undetected | No concerns | Some concerns | Some concerns | No concerns | **Low** |
| **H-HES:Hyper-Alb** | 1 | Some concerns | Undetected | No concerns | No concerns | Major concerns | Major concerns | **Very low** |
| **H-HES:L-HES** | 1 | No concerns | Undetected | No concerns | Major concerns | No concerns | No concerns | **Low** |
| **H-HES:Saline** | 1 | No concerns | Undetected | No concerns | Major concerns | No concerns | No concerns | **Low** |
| **Iso-Alb:Saline** | 1 | Some concerns | Undetected | No concerns | Major concerns | No concerns | Major concerns | **Very low** |
| **L-HES:Saline** | 1 | No concerns | Undetected | No concerns | Major concerns | No concerns | No concerns | **Low** |
| **Gelatin:Hyper-Alb** | 0 | Some concerns | Undetected | No concerns | Major concerns | No concerns | Major concerns | **Very low** |
| **Gelatin:Iso-Alb** | 0 | Some concerns | Undetected | No concerns | Major concerns | No concerns | Major concerns | **Very low** |
| **H-HES:Iso-Alb** | 0 | Some concerns | Undetected | No concerns | Major concerns | No concerns | Major concerns | **Very low** |
| **Hyper-Alb:Iso-Alb** | 0 | Some concerns | Undetected | No concerns | Major concerns | No concerns | Major concerns | **Very low** |
| **Hyper-Alb:L-HES** | 0 | No concerns | Undetected | No concerns | Some concerns | Some concerns | Major concerns | **Very low** |
| **Hyper-Alb:Saline** | 0 | Some concerns | Undetected | No concerns | No concerns | Major concerns | Major concerns | **Very low** |
| **Iso-Alb:L-HES** | 0 | No concerns | Undetected | No concerns | Major concerns | No concerns | Major concerns | **Very low** |

Abbreviations: BC, balanced crystalloids; Iso-Alb, iso-oncotic albumin; Hyper-Alb, hyperoncotic albumin; L-HES, low molecular weight hydroxyethyl starch; H-HES, high molecular weight hydroxyethyl starch.

## 13.3. Trauma patients

13.3.1 Confidence rating in trauma trails for mortality

eTable 13.9. Grading the evidence in traumatic patients for mortality

| **Comparison** | **Studies** | **Within-study bias** | **Reporting bias** | **Indirectness** | **Imprecision** | **Heterogeneity** | **Incoherence** | **Confidence rating** |
| --- | --- | --- | --- | --- | --- | --- | --- | --- |
| **BC:H-HES** | 1 | Some concerns | Undetected | No concerns | Major concerns | No concerns | No concerns | **Very low** |
| **BC:Hyper-Alb** | 1 | No concerns | Undetected | No concerns | Major concerns | No concerns | No concerns | **Low** |
| **BC:Iso-Alb** | 1 | No concerns | Undetected | No concerns | Major concerns | No concerns | No concerns | **Low** |
| **BC:Saline** | 3 | Some concerns | Undetected | No concerns | Major concerns | No concerns | No concerns | **Very low** |
| **Gelatin:H-HES** | 1 | No concerns | Undetected | No concerns | Major concerns | No concerns | No concerns | **Low** |
| **Iso-Alb:Saline** | 1 | No concerns | Undetected | No concerns | Major concerns | No concerns | No concerns | **Low** |
| **L-HES:Saline** | 2 | No concerns | Undetected | No concerns | Major concerns | No concerns | No concerns | **Low** |
| **BC:Gelatin** | 0 | Some concerns | Undetected | No concerns | Major concerns | No concerns | No concerns | **Very low** |
| **BC:L-HES** | 0 | No concerns | Undetected | No concerns | Major concerns | No concerns | No concerns | **Low** |
| **Gelatin:Hyper-Alb** | 0 | No concerns | Undetected | No concerns | Major concerns | No concerns | No concerns | **Low** |
| **Gelatin:Iso-Alb** | 0 | No concerns | Undetected | No concerns | Major concerns | No concerns | No concerns | **Low** |
| **Gelatin:L-HES** | 0 | No concerns | Undetected | No concerns | Major concerns | No concerns | No concerns | **Low** |
| **Gelatin:Saline** | 0 | Some concerns | Undetected | No concerns | Major concerns | No concerns | No concerns | **Very low** |
| **H-HES:Hyper-Alb** | 0 | Some concerns | Undetected | No concerns | Major concerns | No concerns | No concerns | **Very low** |
| **H-HES:Iso-Alb** | 0 | Some concerns | Undetected | No concerns | Major concerns | No concerns | No concerns | **Very low** |
| **H-HES:L-HES** | 0 | Some concerns | Undetected | No concerns | Major concerns | No concerns | No concerns | **Very low** |
| **H-HES:Saline** | 0 | Some concerns | Undetected | No concerns | Major concerns | No concerns | No concerns | **Very low** |
| **Hyper-Alb:Iso-Alb** | 0 | No concerns | Undetected | No concerns | Major concerns | No concerns | No concerns | **Low** |
| **Hyper-Alb:L-HES** | 0 | No concerns | Undetected | No concerns | Major concerns | No concerns | No concerns | **Low** |
| **Hyper-Alb:Saline** | 0 | No concerns | Undetected | No concerns | Major concerns | No concerns | No concerns | **Low** |
| **Iso-Alb:L-HES** | 0 | No concerns | Undetected | No concerns | Major concerns | No concerns | No concerns | **Low** |

Abbreviations: BC, balanced crystalloids; Iso-Alb, iso-oncotic albumin; Hyper-Alb, hyperoncotic albumin; L-HES, low molecular weight hydroxyethyl starch; H-HES, high molecular weight hydroxyethyl starch.

13.3.2 Confidence rating in trauma trails for acute kidney injury

eTable 13.10. Grading the evidence in traumatic patients for acute kidney injury

| **Comparison** | **Studies** | **Within-study bias** | **Reporting bias** | **Indirectness** | **Imprecision** | **Heterogeneity** | **Incoherence** | **Confidence rating** |
| --- | --- | --- | --- | --- | --- | --- | --- | --- |
| **BC:Saline** | 2 | No concerns | Undetected | No concerns | Major concerns | No concerns | Major concerns | **Very low** |
| **L-HES:Saline** | 1 | No concerns | Undetected | No concerns | Major concerns | No concerns | Major concerns | **Very low** |
| **BC:L-HES** | 0 | No concerns | Undetected | No concerns | Major concerns | No concerns | Major concerns | **Very low** |

Abbreviations: BC, balanced crystalloids; Iso-Alb, iso-oncotic albumin; Hyper-Alb, hyperoncotic albumin; L-HES, low molecular weight hydroxyethyl starch; H-HES, high molecular weight hydroxyethyl starch.

## 13.4. Traumatic brain injury patients

eTable 13.11. Grading the evidence in traumatic brain injury patients for mortality

| **Comparison** | **Studies** | **Within-study bias** | **Across-studies bias** | **Indirectness** | **Imprecision** | **Heterogeneity** | **Incoherence** | **Confidence rating** |
| --- | --- | --- | --- | --- | --- | --- | --- | --- |
| **BC:Saline** | 2 | Some concerns | Undetected | No concerns | Some concerns | Some concerns | Major concerns | **Very low** |
| **BC:Iso-Alb** | 0 | No concerns | Undetected | No concerns | Some concerns | Some concerns | Major concerns | **Low** |
| **BC:L-HES** | 0 | No concerns | Undetected | No concerns | Major concerns | No concerns | Major concerns | **Very low** |
| **Saline: Iso-Alb** | 1 | No concerns | Undetected | No concerns | No concerns | Major concerns | Major concerns | **Very low** |
| **Saline:L-HES** | 1 | No concerns | Undetected | No concerns | Major concerns | No concerns | Major concerns | **Very low** |
| **Alb:L-HES** | 0 | No concerns | Undetected | No concerns | Major concerns | No concerns | Major concerns | **Very low** |

Abbreviations: BC, balanced crystalloids; Iso-Alb, iso-oncotic albumin; Hyper-Alb, hyperoncotic albumin; L-HES, low molecular weight hydroxyethyl starch; H-HES, high molecular weight hydroxyethyl starch.

# Appendix 14: Sensitivity analysis

# 14.1. Exclusion with largest trial (The SMART randomized trial)

## 14.1.1. Mortality

eTable 14.1. Mortality in league table in sepsis patients, exclusion of SMART trial

| **Balanced crystalloids** | 0.91  (0.63,1.32) | 0.78  (0.58,1.06) | 0.80  (0.63,1.01) | 0.88  (0.66,1.18) | 0.98  (0.70,1.38) | 0.82  (0.63,1.06) |
| --- | --- | --- | --- | --- | --- | --- |
| **0.84  (0.74,0.95)** | **Saline** | 1.19  (0.95,1.51) | 1.07  (0.92,1.24) | 0.97  (0.84,1.12) | 0.95  (0.67,1.35) | 1.11  (0.79,1.55) |
| 1.00  (0.77,1.30) | 0.84 (0.66,1.06) | **Iso-oncotic**  **albumin** | 0.90  (0.68,1.18) | 0.81  (0.62,1.07) | 0.80  (0.53,1.19) | 0.93  (0.62,1.39) |
| 0.90  (0.71,1.09) | 0.93  (0.81,1.08) | 1.12  0.85,1.47) | **Hyperoncotic albumin** | 0.91  (0.74,1.11) | 0.89  (0.61,1.29) | 1.04  (0.72,1.48) |
| **0.81 (0.69,0.95)** | 1.03  (0.91,1.18) | 0.23  (0.95,1.61) | 1.11  (0.91,1.34) | **L-HES** | 0.98  (0.70,1.38) | 1.14  (0.82,1.60) |
| 0.79  (0.56,1.06) | 1.06  (0.78,1.45) | 1.27 (0.87,1.84) | 1.14  (0.81,1.60) | 1.03  (0.74,1.42) | **H-HES** | 1.16  (0.79,1.72) |
| 0.92  (0.66,1.29) | 0.91  (0.65,1.26) | 1.08  (0.73,1.61) | 0.97  (0.68,1.38) | 0.88  (0.63,1.23) | 0.85  (0.58, 1.25) | **Gelatin** |

Abbreviations: L-HES, Low molecular weight hydroxyethyl starch; H-HES, High molecular weight hydroxyethyl starch

*Results of the network meta-analysis are presented in the lower left triangle. **Sensitivity analysis with exclusion of the SMART randomized trial are presented in the upper right triangle.**

Odd ratio and 95% confidence interval were presented and left hand side intervention was reference group. Odds ratio less than one favor the column-defining treatment.

## 14.1.2. Fluid resuscitation volume

eTable 14.2. Fluid resuscitation volume in league table in sepsis patients, exclusion of SMART trial

| **Balanced crystalloids** | 673.51  (-2317.14,3664.17) | 1830.75  (-773.55,4435.04) | 644.59  (-1339.31,2628.48) | 1873.51  (-1116.91,4863.94) | 2323.67  (-337.73,4985.08) | 384.24  (-1604.30,2372.78) |
| --- | --- | --- | --- | --- | --- | --- |
| 119.71  (-1528.21,1767.62) | **Saline** | 1939.44  (-18.02,3896.89) | 1489.28  (-1416.56,4395.11) | 260.35  (-1315.11,1835.80) | 1446.51  (-331.91,3224.92) | 289.28  (-2616.80,3195.35) |
| 2075.92  (-330.42,4482.27) | **1956.22**  **(71.96,3840.47)** | **Iso-oncotic**  **albumin** | -450.16  (-3865.76,2965.44) | -1679.09  (-3957.11,598.93) | -492.93  (-2734.47,1748.62) | -1650.16  (-5065.96,1765.64) |
| 1721.98  (-1102.55,4546.52) | 1602.28  (-1160.78,4365.33) | -353.94  (-3624.40,2916.52) | **Hyperoncotic albumin** | -1228.93  (-4133.60,1675.74) | -42.77  (-3416.30,3330.76) | -1200.00  (-4542.90,2142.90) |
| 456.83  (-1333.69,2247.35) | 337.12  (-1148.43,1822.67) | -1619.10  (-3802.63,564.44) | -1265.16  (-4057.29,1526.97) | **L-HES** | 1186.16  (-1116.39,3488.71) | 28.93  (-2875.98,2933.83) |
| 1570.82  (-760.32,3901.96) | 1451.11  (-263.60,3165.82) | -505.11  (-2663.23,1653.02) | -151.17  (-3376.47,3074.14) | 1113.99  (-1089.08,3317.07) | **H-HES** | -1157.23  (-4530.96,2216.50) |
| 521.98  (-2302.80,3346.76) | 402.28  (-2361.03,3165.58) | -1553.94  (-4824.61,1716.73) | -1200.00  (-4415.80,2015.80) | 65.16  (-2727.22,2857.53) | -1048.83  (-4274.35,2176.69) | **Gelatin** |

Abbreviations: L-HES, Low molecular weight hydroxyethyl starch; H-HES, High molecular weight hydroxyethyl starch

* Results of the network meta-analysis are presented in the lower left triangle. **Sensitivity analysis with exclusion of the SMART randomized trial are presented in the upper right triangle.**

Odd ratio and 95% confidence interval were presented and left hand side intervention was reference group. Odds ratio less than one favor the column-defining treatment.

## 14.1.3. Acute kidney injury

eTable 14.3. Acute kidney injury in league table in sepsis patients, exclusion of SMART trial

| **Balanced crystalloids** | 1.26  (0.54,2.95) | **0.54**  **(0.37,0.79)** | 0.79  (0.60,1.05) | 0.92  (0.58,1.46) | 0.96  (0.67,1.36) |
| --- | --- | --- | --- | --- | --- |
| 0.98  (0.82,1.17) | **Saline** | 0.97  (0.72,1.29) | 0.83  (0.66,1.04) | **0.57**  **(0.34,0.95)** | 1.32  (0.53,3.30) |
| 0.95  (0.68,1.33) | 0.97  (0.72,1.29) | **Iso-oncotic**  **albumin** | 0.86  (0.59,1.24) | 0.59  (0.33,1.06) | 1.37  (0.52,3.57) |
| **0.80**  **(0.65,0.99)** | 0.82  (0.67,1.00) | 0.85  (0.60,1.20) | **L-HES** | 0.69  (0.43,1.10) | 1.60  (0.66,3.90) |
| **0.54**  **(0.37,0.79)** | **0.55**  **(0.37,0.84)** | **0.57**  **(0.35,0.95)** | 0.68  (0.44,1.04) | **H-HES** | 2.32  (1.09,4.96) |
| 1.26  (0.54,2.95) | 1.29  (0.54,3.05) | 1.33  (0.53,3.32) | 1.57  (0.66,3.77) | **2.32**  **(1.09,4.96)** | **Gelatin** |

Abbreviations: L-HES, Low molecular weight hydroxyethyl starch; H-HES, High molecular weight hydroxyethyl starch

* Results of the network meta-analysis are presented in the lower left triangle. **Sensitivity analysis with exclusion of the SMART randomized trial are presented in the upper right triangle.**

Odd ratio and 95% confidence interval were presented and left hand side intervention was reference group. Odds ratio less than one favor the column-defining treatment.

## 14.1.4. Blood cell transfusion volume

eTable 14.4. Blood cell transfusion volume in league table in sepsis patients, exclusion of SMART trial

| **Balanced crystalloids** | 42.31  (-229.81,314.42) | 61.08  (-282.22,404.39) | 287.44  (-52.64,627.53) | 244.91  (-11.65,501.47) | **500.00**  **(117.57,882.43)** |
| --- | --- | --- | --- | --- | --- |
| -28.47  (-215.20,158.26) | **Saline** | 18.77  (-205.57,243.12) | **245.13**  **(4.38,485.89)** | **202.60**  **(37.00,368.19)** | 457.69  (-11.67,927.05) |
| -50.54  (-322.44,221.36) | -22.07  (-226.89,182.75) | **Iso-oncotic**  **albumin** | 226.36  (-94.59,547.31) | 183.82  (-74.60,442.25) | 438.92  (-75.00,952.84) |
| **-274.86**  **(-547.82,-1.89)** | **-246.38**  **(-463.94,-28.83)** | -224.31  (-516.63,68.00) | **Hyperoncotic albumin** | -42.54  (-287.58,202.51) | 212.56  (-299.22,724.33) |
| **-231.93**  **(-429.57,-34.29)** | **-203.46**  **(-349.29,-57.63)** | -181.39  (-416.57,53.79) | 42.92  (-179.10,264.94) | **L-HES** | 255.09  (-205.43,715.61) |
| **-500.00**  **(-857.79,-142.21)** | **-471.53**  **(-875.12,-67.94)** | **-449.46**  **(-898.85,-0.07)** | -225.14  (-675.18,224.89) | -268.07  (-676.82,140.68) | **H-HES** |

Abbreviations: L-HES, Low molecular weight hydroxyethyl starch; H-HES, High molecular weight hydroxyethyl starch

* Results of the network meta-analysis are presented in the lower left triangle. **Sensitivity analysis with exclusion of the SMART randomized trial are presented in the upper right triangle.**

Odd ratio and 95% confidence interval were presented and left hand side intervention was reference group. Odds ratio less than one favor the column-defining treatment.

# 14.2. Inclusion with pilot study (The SALT Randomized Trial)

## 14.2.1. Mortality

eTable 14.5. Mortality in league table in sepsis patients with pilot study (The SALT Randomized Trial)

| **Balanced crystalloids** | **0.84**  **(0.75,0.95)** | 1.01  (0.78,1.30) | 0.90  (0.75,1.09) | **0.81**  **(0.70,0.95)** | 0.79  (0.59,1.06) | 0.93  (0.66,1.30) |
| --- | --- | --- | --- | --- | --- | --- |
| **0.84  (0.74,0.95)** | **Saline** | 0.84  (0.66,1.06) | 0.93  (0.81,1.08) | 1.04  (0.91,1.18) | 1.07  (0.79,1.45) | 0.91  (0.66,1.26) |
| 1.00  (0.77,1.30) | 0.84 (0.66,1.06) | **Iso-oncotic**  **albumin** | 1.12  (0.85,1.47) | 1.24  (0.95,1.61) | 1.28  (0.88,1.85) | 1.08  (0.73,1.61) |
| 0.90  (0.71,1.09) | 0.93  (0.81,1.08) | 1.12  0.85,1.47) | **Hyperoncotic albumin** | 1.11  (0.91,1.35) | 1.15  (0.82,1.61) | 0.97  (0.68,1.39) |
| **0.81 (0.69,0.95)** | 1.03  (0.91,1.18) | 0.23  (0.95,1.61) | 1.11  (0.91,1.34) | **L-HES** | 1.03  (0.75,1.42) | 0.88  (0.63,1.23) |
| 0.79  (0.56,1.06) | 1.06  (0.78,1.45) | 1.27 (0.87,1.84) | 1.14  (0.81,1.60) | 1.03  (0.74,1.42) | **H-HES** | 0.85  (0.58,1.24) |
| 0.92  (0.66,1.29) | 0.91  (0.65,1.26) | 1.08  (0.73,1.61) | 0.97  (0.68,1.38) | 0.88  (0.63,1.23) | 0.85  (0.58, 1.25) | **Gelatin** |

Abbreviations: L-HES, Low molecular weight hydroxyethyl starch; H-HES, High molecular weight hydroxyethyl starch

*Results of the network meta-analysis are presented in the lower left triangle. **Sensitivity analysis with the SALT randomized trial are presented in the upper right triangle.**

Odd ratio and 95% confidence interval were presented and left hand side intervention was reference group. Odds ratio less than one favor the column-defining treatment.

## 14.2.2. Acute kidney injury

eTable 14.6. Acute kidney injury in league table in sepsis patients with pilot study (The SALT Randomized Trial)

| **Balanced crystalloids** | 1.26  (0.54,2.95) | **0.54**  **(0.37,0.79)** | **0.79**  **(0.64,0.97)** | 0.92  (0.66,1.28) | 0.95  (0.82,1.11) |
| --- | --- | --- | --- | --- | --- |
| 0.98  (0.82,1.17) | **Saline** | 0.97  (0.72,1.29) | **0.83**  **(0.68,1.00)** | **0.57**  **(0.38,0.85)** | 1.32  (0.56,3.13) |
| 0.95  (0.68,1.33) | 0.97  (0.72,1.29) | **Iso-oncotic**  **albumin** | 0.86 (0.60,1.21) | **0.59**  **(0.36,0.97)** | 1.37  (0.55,3.40) |
| **0.80**  **(0.65,0.99)** | 0.82  (0.67,1.00) | 0.85  (0.60,1.20) | **L-HES** | 0.69  (0.45,1.06) | 1.60  (0.67,3.83) |
| **0.54**  **(0.37,0.79)** | **0.55**  **(0.37,0.84)** | **0.57**  **(0.35,0.95)** | 0.68  (0.44,1.04) | **H-HES** | 2.32  (1.09,4.96) |
| 1.26  (0.54,2.95) | 1.29  (0.54,3.05) | 1.33  (0.53,3.32) | 1.57  (0.66,3.77) | **2.32**  **(1.09,4.96)** | **Gelatin** |

Abbreviations: L-HES, Low molecular weight hydroxyethyl starch; H-HES, High molecular weight hydroxyethyl starch

*Results of the network meta-analysis are presented in the lower left triangle. **Sensitivity analysis with the SALT randomized trial are presented in the upper right triangle.**

Odd ratio and 95% confidence interval were presented and left hand side intervention was reference group. Odds ratio less than one favor the column-defining treatment.
